# Histone modification analysis reveals common regulators of gene expression in liver and blood stage merozoites of *Plasmodium* parasites

**DOI:** 10.1186/s13072-023-00500-y

**Published:** 2023-06-15

**Authors:** Ashley B. Reers, Rodriel Bautista, James McLellan, Beatriz Morales, Rolando Garza, Sebastiaan Bol, Kirsten K. Hanson, Evelien M. Bunnik

**Affiliations:** 1 Department of Microbiology, Immunology, and Molecular Genetics, Long School of Medicine, University of Texas Health Science Center, San Antonio, TX USA; 2 Department of Molecular Microbiology and Immunology and South Texas Center for Emerging Infectious Diseases, University of Texas San Antonio, San Antonio, TX USA

**Keywords:** Malaria, Epigenetics, Gene regulation, Transcriptomics, Chromatin immunoprecipitation

## Abstract

Gene expression in malaria parasites is subject to various layers of regulation, including histone post-translational modifications (PTMs). Gene regulatory mechanisms have been extensively studied during the main developmental stages of *Plasmodium* parasites inside erythrocytes, from the ring stage following invasion to the schizont stage leading up to egress. However, gene regulation in merozoites that mediate the transition from one host cell to the next is an understudied area of parasite biology. Here, we sought to characterize gene expression and the corresponding histone PTM landscape during this stage of the parasite lifecycle through RNA-seq and ChIP-seq on *P. falciparum* blood stage schizonts, merozoites, and rings, as well as *P. berghei* liver stage merozoites. In both hepatic and erythrocytic merozoites, we identified a subset of genes with a unique histone PTM profile characterized by a region of H3K4me3 depletion in their promoter. These genes were upregulated in hepatic and erythrocytic merozoites and rings, had roles in protein export, translation, and host cell remodeling, and shared a DNA motif. These results indicate that similar regulatory mechanisms may underlie merozoite formation in the liver and blood stages. We also observed that H3K4me2 was deposited in gene bodies of gene families encoding variant surface antigens in erythrocytic merozoites, which may facilitate switching of gene expression between different members of these families. Finally, H3K18me and H2K27me were uncoupled from gene expression and were enriched around the centromeres in erythrocytic schizonts and merozoites, suggesting potential roles in the maintenance of chromosomal organization during schizogony. Together, our results demonstrate that extensive changes in gene expression and histone landscape occur during the schizont-to-ring transition to facilitate productive erythrocyte infection. The dynamic remodeling of the transcriptional program in hepatic and erythrocytic merozoites makes this stage attractive as a target for novel anti-malarial drugs that may have activity against both the liver and blood stages.

**Supplementary Information:**

The online version contains supplementary material available at 10.1186/s13072-023-00500-y.

## Introduction

Malaria remains a significant source of morbidity and mortality, particularly in children under 5 years of age in Africa [1]. Increasing resistance of *Plasmodium falciparum* to currently available anti-malarial drugs highlights the need for novel ant-imalarial drugs [1]. As such, improved understanding of parasite biology will facilitate the design of novel treatments for malaria. The processes that regulate transcription in *Plasmodium* parasites are considered attractive candidates for such new therapies [2–4]. Much effort has been put into understanding these mechanisms during the main developmental stages (ring, trophozoite, and schizont) of the intra-erythrocytic developmental cycle (IDC). However, the development of mature schizonts prior to egress into young rings following re-invasion has thus far mostly been overlooked. This transition is facilitated by the merozoite stage, a specialized parasite form that functions to move between host cells. This happens at two points in the parasite lifecycle. During the transition from the liver stage to the IDC, merozoites facilitate transmission from hepatocytes to erythrocytes. During the IDC, they enable the transition from one erythrocyte to the next. These transitions are crucial phases of the parasite life cycle as they facilitate the establishment and continuation of blood stage infection [5]. Similar regulatory mechanism may underlie the development of hepatic and erythrocytic merozoites. However, erythrocytic merozoites are highly transient in culture and have lower RNA content than most other stages, making them technically challenging to study [6]. For this reason, merozoites have been understudied in comparison to other IDC stages. Understanding how parasites mediate changes in their transcriptional program during these transitional phases may reveal novel aspects of parasite biology. As such, it may present novel directions for anti-malarial approaches that target both the liver and asexual blood stage of *Plasmodium*.

Gene expression is tightly regulated during all stages of *Plasmodium* development [7–9]. The correct temporal expression of genes is crucial to the survival of parasites, particularly during stage transitions when parasites must undergo dramatic transcriptional and translational changes to establish themselves in a new environment [10, 11]. To allow for mosquito-to-human transmission, *Plasmodium* parasites must form infectious sporozoites. This process is dependent on reprograming of the parasite transcriptome and proteome which occurs as sporozoites travel from the mosquito midgut to the salivary glands [12, 13]. Infectious sporozoites upregulate the expression of genes involved in sporozoite maturation, motility, parasite-host interactions, metabolism, and liver-stage development [14–16]. Similarly, following uptake by blood-feeding mosquitos, gametocytes undergo changes in transcriptional program with activation of roughly 20% of stage-specific genes during this transition [7, 17–19]. Transitioning from one erythrocyte to the next during the IDC also requires a dramatic developmental change. The later stages of schizont development are characterized by the production of proteins required for invasion of a new host cell. Before rupturing from the current erythrocyte in the form of merozoites, expression of these genes is downregulated. After invading a new erythrocyte, the parasite requires a different gene expression program with a focus on metabolism, translation, and host cell remodeling to successfully establish itself as a ring in the new erythrocyte and continue progressing through the IDC [7, 8, 20, 21]. This transcriptional shift is crucial for correct development, but how the parasite controls gene expression during egress and reinvasion is largely unknown.

Compared to other eukaryotic organisms, *Plasmodium* parasites have a relatively small number of sequence-specific transcription factors, meaning the parasite must rely on other mechanisms to orchestrate its transcriptional program [22, 23]. Epigenetic regulation of gene expression through the dynamic distribution of histone post-translational modifications (PTMs) is an important process in the control of stage-specific gene expression during the IDC. Temporal changes in histone PTM distribution in intergenic regions have been shown to regulate *Plasmodium* gene transcription [24, 25]. H3K9ac, H3K27ac, and H3K4me3 are well-known histone PTMs associated with active gene expression [26–29]. Changes in the levels of H3K4me3 and the related mark H3K4me2 between schizonts and rings suggest a major epigenetic shift during egress and reinvasion [26, 30]. Histone marks associated with gene repression are also present in *Plasmodium*, but, apart from H3K9me3 that is found in subtelomeric and internal virulence gene clusters and involved in heterochromatin formation and maintenance, their distribution and precise function are not well understood. Mass spectrometry analysis identified H3K27me deposition exclusively in late schizonts and early rings, indicating that this mark may have roles in regulating gene expression in merozoites [31]. H3K18me is deposited in the later stages of *Plasmodium* IDC development, but its function has not been studied [31]. However, in the closely related *Theileria* parasites, H3K18me is known to repress gene expression during the schizont stage and is removed in merozoites, suggesting a potential role in regulating gene expression during the schizont-to-ring transition [32]. Combined, these studies demonstrate gene regulation by dynamic distribution of histone PTMs during parasite development. However, exclusion of merozoites from these analyses in *P. falciparum* limits insight into changes in the distribution of histone PTMs during egress and re-invasion.

Here, we sought to understand how changes in gene expression are orchestrated during the schizont-to-ring transition by characterizing the histone PTM landscape and transcriptional program in *P. falciparum* schizonts, merozoites, and rings. We performed chromatin immunoprecipitation and sequencing (ChIP-seq) along with RNA-seq on populations of schizonts, merozoites, and rings to assess differences in activating and repressive histone PTMs between the stages and corresponding changes in gene expression. To gain insight into potential similarities in gene regulation between erythrocytic and hepatic merozoite development, we also assessed the association between gene expression and histone PTMs in *P. berghei* hepatic merozoites. Collectively, our results show that merozoites undergo dynamic remodeling of the histone PTM landscape. In particular, a subset of genes that is necessary to establish a productive erythrocyte infection is marked by a specific histone PTM pattern in both erythrocytic and hepatic merozoites, suggesting that common regulatory mechanisms underlie merozoite development in both the liver and blood stage.

## Results

### RNA-seq identifies rapid changes in gene expression during the schizont-to-ring transition

While gene expression in the main phases of the IDC has been studied in detail, limited insight into gene expression in merozoites and how it relates to the other stages is available [7, 20]. Given that the transition from one erythrocyte to the next requires numerous changes in parasite biology, we were interested in characterizing transcriptional changes that occur in parasites during merozoite formation. To this end, we performed RNA-seq using three biological replicates of synchronized early schizonts (40 h post infection or hpi) and merozoites collected as outlined in Additional file ﻿1: Fig. S1A. The 40 hpi schizont time point was selected because parasites collected at a later time point (44–48 hpi) may already include fully segmented schizonts, due to the challenges of fully synchronizing *P. falciparum* cultures. Merozoites were prepared by treating early schizonts (collected at 40 hpi) with the egress inhibitor E64. E64-treated parasites progress through schizogony normally, but are blocked at the final step of egress, just prior to rupturing of the erythrocyte membrane. Fully segmented schizonts were then mechanically ruptured to release merozoites.

**Fig. 1:**
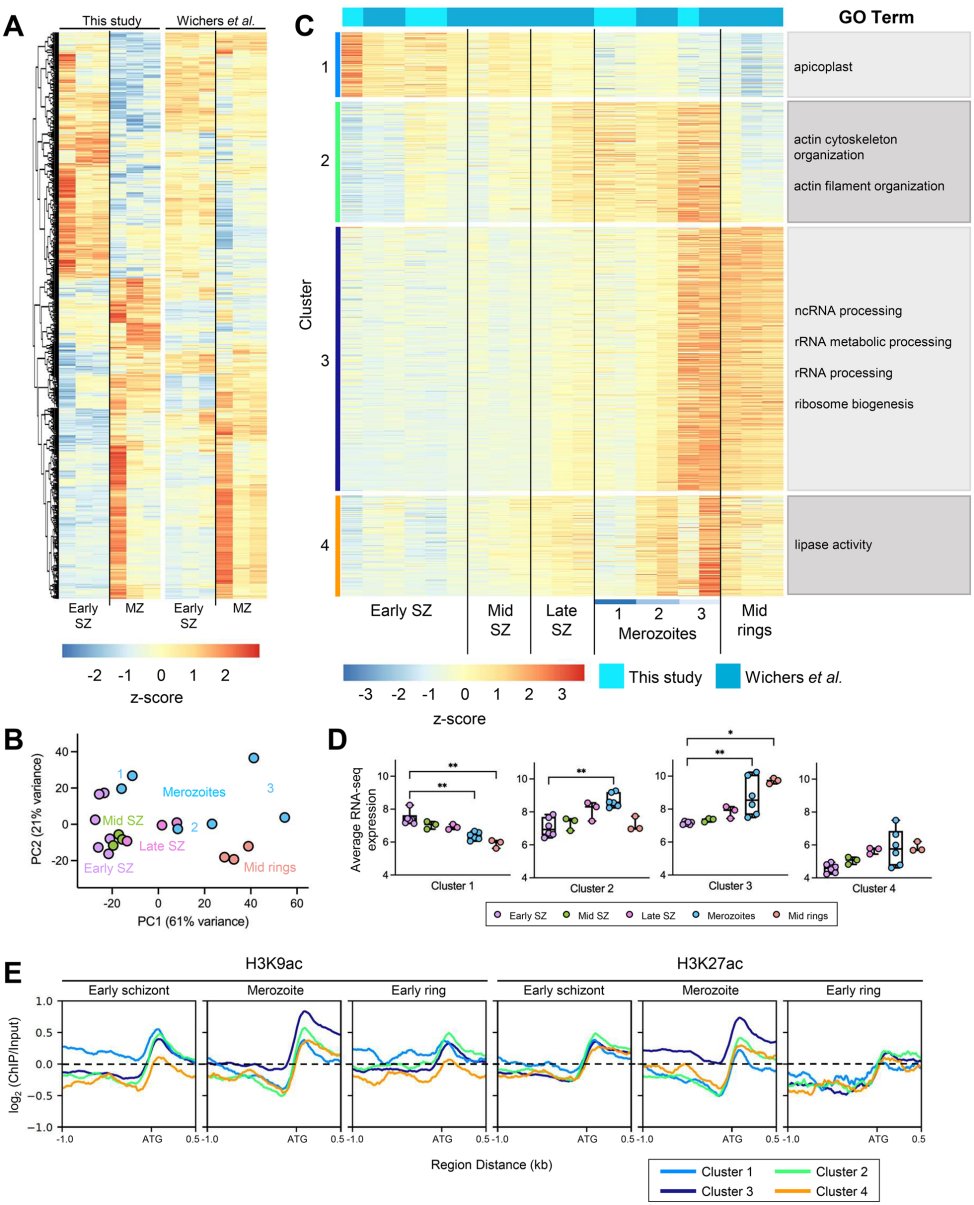
Gene expression changes rapidly during transitions between schizont, merozoite, and ring stage parasites.** A** Heatmap depicting differentially expressed genes (log_2_(fold change) > 1.5 or < -1.5 and adjusted p-value < 0.1) between early schizont samples (40 hpi, n = 3) and merozoites (MZ, n = 3), all collected by us. Expression of these genes in early schizont (40 hpi, n = 3) and merozoite (MZ, n = 3) samples generated by Wichers et al*.* are shown on the right. **B** Principal component analysis of all RNA-seq samples. Merozoite subpopulations defined in panel C are indicated by blue numbers. Amount of variance between samples accounted for by each component is shown on the x- and y- axes. **C** Heatmap depicting differentially expressed genes (log_2_(fold change) > 1.5 or < -1.5 and adjusted p-value < 0.1) between early schizont samples (40 hpi, n = 6) and merozoites (n = 6). Genes are divided into 4 clusters based on expression pattern by k-means clustering as indicated on the left of the heatmap. Merozoite subpopulations are indicated by the labels on the bottom of the heatmap. Enriched gene ontology (GO) terms in each cluster are displayed to the right of the heatmap. **D** Boxplots of average gene expression for each group as shown in panel C. Gene expression differed between groups (Kruskal–Wallis test). *P* values indicated in the graph are from Dunn’s post hoc tests. *, *P* < 0.05; **, *P* < 0.01. For all boxplots, box edges indicate the first and third quartiles, mid-line indicates the median, and whiskers indicate the minimum and maximum data values. **E** H3K9ac and H3K27ac expression shown as log_2_(ChIP/input) around the translation start site (ATG) for genes from each group as indicated in panel C in early schizonts (40 hpi), merozoites, and early rings (4 hpi). Graphs were generated using computeMatrix from the deepTools package

To interrogate differences in gene expression between early schizonts (40 hpi) and merozoites, we performed differential gene expression analysis with these two parasite populations. We observed two sets of differentially expressed genes between these populations, demonstrating that as parasites transition from schizonts to merozoites, the transcriptional landscape is altered (Fig. 1A, Additional file 2: Tables S1, S2). Notably, a group of genes appear to be turned on as parasites transition to merozoites, countering the idea that this stage is transcriptionally silent. Unexpectedly, we observed prominent heterogeneity in our merozoite samples. To probe whether this was the result of technical issues in our sample preparation and sequencing or reflective of true biological differences between replicates, we took advantage of data available from a recent study by Wichers et al*.* [33]. This study generated RNA-seq data at several time points across the IDC, including early schizonts (40 hpi) and merozoites. For the genes that were upregulated in merozoites as compared to schizonts in our data set, the merozoite samples from Wichers et al*.* also displayed prominent variability (Fig. 1A, Additional file 2: Table S2). Importantly, the trends in expression observed in our data, including upregulation of a subset of genes, were also present in the Wichers et al*.* data set, demonstrating that despite heterogeneity within timepoints, both data sets captured similar transcriptional changes between early schizonts and merozoites.

Given the heterogeneity in merozoite samples and the similarities between the two data sets, we chose to combine our data with that from Wichers et al*.* for further analysis. In addition to the early schizont and merozoite samples, Wichers et al*.* also performed RNA-seq on mid schizonts (44 hpi, n = 3), late schizonts (48 hpi, n = 3) and mid rings (8 hpi, n = 3). By including these time points in our analysis, we were able to interrogate changes in transcription at higher resolution across the schizont-to-merozoite transition as well as characterize alterations in gene expression between merozoites and mid rings.

We first performed principal component analysis (PCA) to understand global differences between parasite stages. This analysis reaffirmed the heterogeneity we initially observed in merozoites (Fig. 1B). The first component of the PCA mainly captured differences between the stages, while the second component captured variability between the two data sets (Additional file 1: Fig. S2). Generally, schizont stages (early schizont, 40 hpi; mid schizont, 44 hpi; late schizont, 48 hpi) clustered together and mid ring stage parasites (8 hpi) clustered separately. The merozoite population had the most spread, with some samples showing similarity to early schizonts (subpopulation 1), some split between late schizonts and mid rings (subpopulation 2), and the final set located near the mid rings (subpopulation 3) (Fig. 1B). This suggests that within the six merozoite samples, three subpopulations were captured.

To characterize the differences in gene expression across the schizont-to-ring transition in more detail, we performed differential gene expression analysis on the combined data set. Since we observed significant differences in expression between early schizonts and merozoites in the initial analysis of only our samples (Fig. 1A) we sought to identify genes that were differentially expressed between early schizonts (40 hpi, n = 6) and merozoites (n = 6). Based on a magnitude log_2_(fold change) > 1.5 and an adjusted p-value < 0.1, we identified 936 differentially expressed genes (Additional file 2: Table S3). We then assessed the expression of these genes in early schizonts (40 hpi), mid schizonts (44 hpi), late schizonts (48 hpi), merozoites, and mid rings (8 hpi) (Fig. 1C, Additional file 2: Table S4). Genes were grouped through k-means clustering based on expression across the stages into 4 clusters (Fig. 1C). Genes expressed from early to late schizonts (n = 110) in our differential gene expression analysis were captured in cluster 1 (Fig. 1C, D). As parasites progressed through the developmental cycle from early schizonts to mid rings, expression of these genes decreased. This cluster was enriched for genes related to the apicoplast as determined by gene ontology (GO) analysis (Additional file 2: Table S5). Genes with increased expression in late schizonts and merozoites (cluster 2, n = 205) were enriched for GO terms relating to actin filament organization and cytoskeleton organization (Additional file 2: Table S5), which are critical processes required for successful invasion (reviewed in [34]). These transcripts are likely rapidly downregulated following invasion, as expression levels in mid rings were comparable to those seen in schizonts (Fig. 1D).

Cluster 3 (n = 450) was comprised of genes expressed at elevated levels in mid rings and enriched for GO terms related to ribosome biogenesis and rRNA processing (Fig. 1D, Additional file 2: Table S5). In merozoites, expression of these genes was heterogeneous. In two merozoite samples (subpopulation 1 in Fig. 1B, C), we observed low levels of expression comparable to that seen in early, mid, and late schizonts. Two merozoite samples (subpopulation 2 in Fig. 1B, C). had expression levels intermediate between that seen in schizonts and that seen in mid rings. In the final two merozoite samples (subpopulation 3 in Fig. 1B, C), we observed high levels of these transcripts, comparable to or higher than the levels observed in mid rings. We hypothesize that heterogeneity in the merozoite samples reflects three different stages along the merozoite developmental trajectory, with expression of genes related to ribosome activity and RNA processing increasing as merozoites mature. This gene expression program appears to reach its highest level in late stage merozoites (subpopulation 3) and continue during ring development. Interestingly, differential gene expression analysis between merozoites and mid rings (8 hpi) revealed that expression of genes involved in DNA replication and mitosis gradually decreased in merozoites (Additional file 1: Fig. S3A, Additional file 2: Tables S3, S6, S7, cluster A, n = 1,253). This analysis also identified a second set of genes enriched for GO terms related to translation and ribosome biogenesis that was upregulated in mid rings (8 hpi) as compared to all other stages (Additional file 1: Fig. S3A, Additional file 2: Tables S3, S6, S7, cluster C, n = 1,057). Combined, our differential gene expression analyses suggests that, prior to invasion, parasites downregulate genes involved in schizogony while upregulating genes required for translation and RNA processing. After invasion, another wave of transcription results in the expression of additional genes involved in translation and metabolism that are likely necessary to further facilitate ring stage development in a new erythrocyte.

Genes in the final cluster of differentially expressed genes between early schizonts and merozoites (cluster 4, n = 171) were elevated in merozoites and mid rings; however, expression of this cluster was more variable than what we observed in the other three clusters (Fig. 1C, D). This cluster was enriched for genes with roles in lipase activity (Additional file 2: Table S7). Interestingly, it appears these genes were consistently elevated in samples from Wichers et al*.* compared to our samples, suggesting variation in this cluster may be due to stochastic differences in parasite populations. Consistent with this possibility, genes known for variable expression within parasite populations, such as *rifins*, were present within this cluster.

Together, these data demonstrate that parasites undergo rapid changes in gene expression during or following segmentation into merozoites, resulting in heterogeneity among merozoite populations.

### Changes in the distribution of H3K9ac and H3K27ac accompany gene expression changes in merozoites

Given the changes in gene expression as parasites transition from early schizonts to mid rings, we were interested in investigating potential mechanisms behind this shift. Epigenetic regulation of gene expression by the distribution of histone PTMs is known to influence stage-specific expression of genes during the IDC [25]. To uncover differences in the histone PTM landscape that could be related to the differences in gene expression we observed in our RNA-seq analysis, we performed ChIP-seq for the histone PTMs known to be associated with active gene expression (H3K9ac, H3K27ac, and H3K4me3) on early schizonts (40 hpi), merozoites, and early rings (4 hpi). Samples for ChIP-seq and RNA-seq were collected separately as outlined in Additional file 1: Fig. S1A, B. Multiple samples from each stage were pooled to control for heterogeneity; however, this means our ChIP-seq data is reflective of the average histone PTM profile across each stage and does not capture differences in histone PTMs that may exist between the three subpopulations of merozoites identified in our RNA-seq analysis.

First, we assessed the similarity of global histone PTM distribution between marks and stages. Generally, within and between stages, the distributions of H3K4me3, H3K9ac, and H3K27ac showed moderate to high correlations (Additional file 1: Fig. S4, blue box). This indicates that within each stage these marks likely colocalize and that, generally, these marks have similar distribution patterns between stages. However, for each mark, the correlation between early schizont and early ring stages was higher than between either of the two stages and merozoites. Additionally, the correlation between H3K9ac and H3K27ac was stronger in merozoites (r = 0.92) than the other two stages (early schizonts, r = 0.56; early rings, r = 0.43). These results suggest that the histone PTM landscape is dynamically remodeled during the schizont-to-ring transition, with a distinct distribution in merozoites.

Given the higher correlation between H3K9ac and H3K27ac in merozoites (compared to the other stages) and their well-known association with active gene expression, we investigated how the distribution of these marks changed during the schizont-to-ring transition. Globally, H3K9ac and H3K27ac followed similar distribution patterns in early schizonts, merozoites, and early rings (Additional file 1: Fig. S5). To probe how differences in gene expression relate to differences in histone PTM distribution, we looked at the enrichment of H3K9ac and H3K27ac for the four clusters of differentially expressed genes identified in our RNA-seq analysis (Fig. 1E). For all groups, both marks had similar distribution patterns with sharp increases at the gene start site and enrichment into the gene body. As expected, genes with higher expression levels in schizonts (cluster 1) had increased H3K9ac in early schizonts. H3K9ac and H3K27ac decreased for this cluster of genes in merozoites, which corresponded with a decrease in transcript levels at this stage. The promoter regions of genes in cluster 3, that were upregulated in merozoites, were enriched in H3K9ac and H3K27ac in merozoites as compared to these marks in the other stages. In rings, the levels of H3K9ac and H3K27ac in these genes reverted back to the levels seen in schizonts, even though the corresponding transcripts were still detected in the mid ring stage. This could be reflective of maintenance of these transcripts when the corresponding genes have already been turned off. Collectively, these results show changes in the distribution of H3K9ac and H3K27ac that are closely related to differences in gene expression as *P. falciparum* transitions from the schizont to ring stage.

### Unique histone PTM profile defined by depletion of H3K4me3 is associated with gene expression in *P. falciparum* merozoites

In addition to the increase in H3K9ac and H3K27ac in merozoites, we observed differences in H3K4me3 between the stages, primarily in the coding regions (Additional file 1: Fig. S5). While all stages had sharp enrichment of this mark at the gene start and end, H3K4me3 was only enriched in the gene body in merozoites. Surprisingly, we observed similar levels of H3K4me3 in schizonts and rings (Additional file 1: Figs. S5 and S6). Previously, this mark was shown to be present at low levels in ring stages, with peak enrichment during the trophozoite stage [26]. Our results suggest that H3K4me3 is relatively constant between early schizonts, merozoites, and early rings with the most prominent difference being enrichment of H3K4me3 in the gene bodies in merozoites (Additional file 1: Fig. S5). In keeping with this previous study, we did not observe changes in H3K4me3 in relation to gene expression (Additional file 1: Fig. S6). Our results suggest that H3K4me3 is present in coding regions throughout the IDC and is uncoupled from gene expression.

**Fig. 2:**
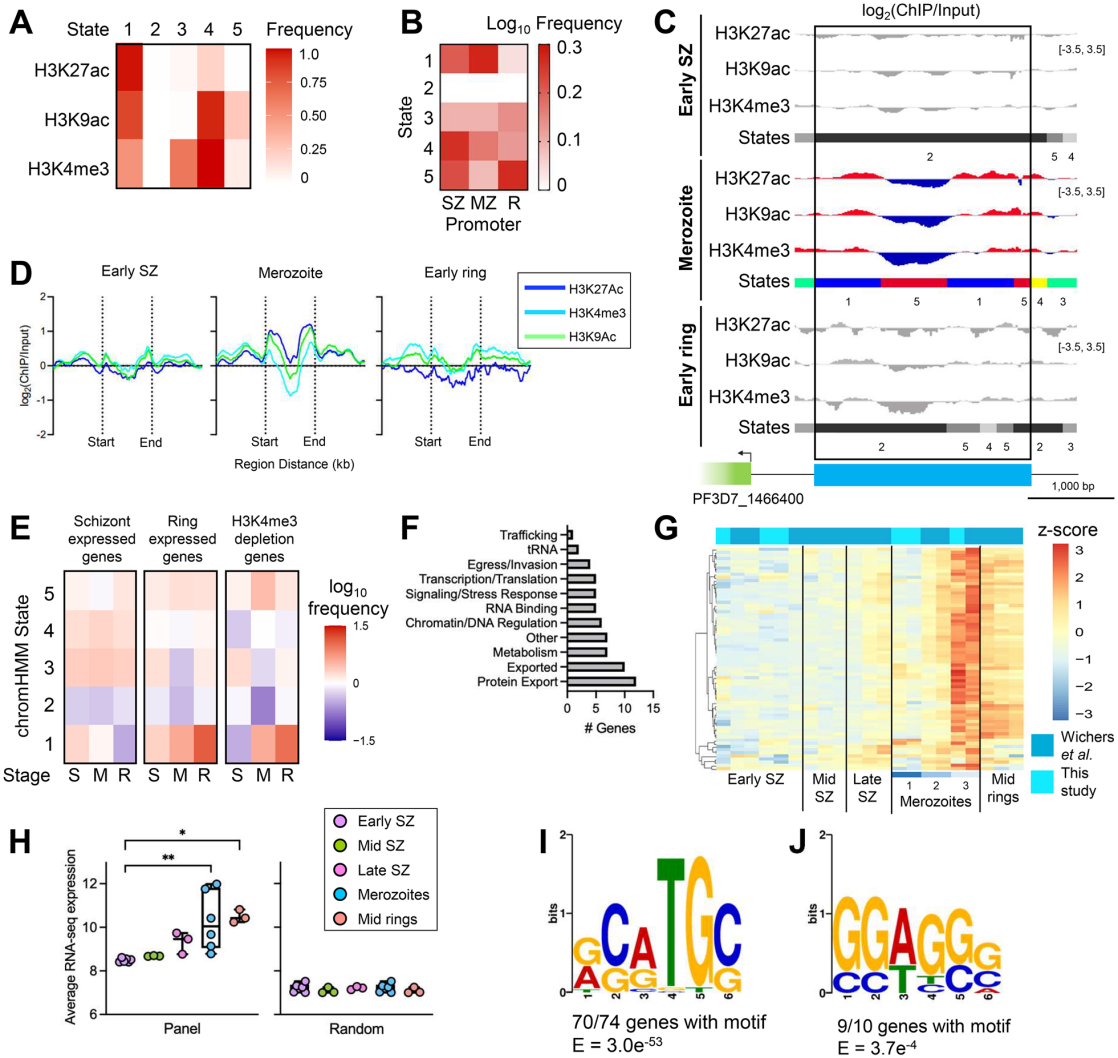
Unique histone remodeling is associated with a subset of merozoite-expressed genes.** A** State emissions profile generated by ChromHMM analysis. **B** Heatmap depicting the frequency of each ChromHMM state in the promoters (1,000 bp region upstream of the gene ATG) of all genes (n = 5,602) in early schizonts (SZ, 40 hpi), merozoites (MZ), and early rings (R, 4 hpi). **C** H3K27ac, H3K9ac, and H3K4me3 ChIP tracks depicting a region of H3K4me3 depletion upstream of AP2-EXP (PF3D7_1466400) and corresponding ChromHMM states. Black rectangle indicates the region where an H3K4me3 depletion is flanked by enrichment of H3K9ac and H3K27ac in merozoites. At the bottom, the AP2-EXP gene body is indicated by a green box, while the region with the 1 – 5 – 1 ChromHMM state pattern is marked by the blue box (coordinates on chromosome 14: 2,719,800–2,722,200 bp). Data range of ChIP tracks is indicated in brackets on the right. Scale bar depicting 1,000 bp is shown. **D** Average ChIP/input of H3K27ac, H3K9ac, and H3K4me3 across 66 regions with H3K4me3 depletion flanked by enrichment of H3K9ac and H3K27ac. Regions were scaled to the same size by computeMatrix, and the enrichment 1.0 kb upstream and downstream of the regions was also graphed. **E** Heatmap depicting the frequency of each ChromHMM state in the promoter region (1.000 bp upstream of the gene ATG) of schizont-expressed genes, ring-expressed genes, and genes with H3K4me3 depletion in the upstream intergenic region in early schizonts (S, 40 hpi), merozoites (M), and early rings (R, 4 hpi). **F** Functions associated with genes with H3K4me3 depletion. Functions were assigned based on information available in PlasmoDB and a review of the literature and can be found in Additional file 2: Table S12. Genes annotated as ‘conserved with unknown function’ (n = 9) in PlasmoDB are not shown in the graph but were included in all analyses. **G** Heatmap depicting normalized gene expression in protein-coding genes with H3K4me3 depletion (n = 70). Four non-coding genes with H3K4me3 depletion are not plotted because these genes were excluded from the RNA-seq differential gene expression analysis. **H** Boxplots depicting average gene expression of the genes with H3K4me3 depletion (panel) and a set of randomly selected genes (n = 99). Gene expression differed between time points for the panel of genes with H3K4me3 depletion (Kruskal–Wallis test). *P* values indicated in the graph are from Dunn’s post hoc tests. **, *P* < 0.01; ***, *P* < 0.001; ****, *P* < 0.0001. **I)** DNA motif in the H3K4me3 depletion regions. **J)** DNA motif in the H3K4me3 depletion regions of genes associated with PTEX

Since H3K9ac and H3K27ac are associated with transcription and H3K4me3 is associated with permissive chromatin, we sought to assess the combinatorial distribution of these marks across the genome. To accomplish this, we performed ChromHMM analysis, a machine learning approach to identifying combinatorial chromatin states [35]. This tool utilizes histone PTM distribution information to develop a model of chromatin states based on the frequency of each mark across the genome in 200 bp segments. Using the ChIP-seq data we generated for H3K9ac, H3K27ac, and H3K4me3 in early schizonts (40 hpi), merozoites, and early rings (4 hpi), we identified 5 unique chromatin states (Fig. 2A, Additional file 2: Tables S8, S9) and annotated the genome with these states to understand the relationship between each chromatin state and specific genomic features (intergenic regions, coding sequences, gene start sites, etc.).

We were interested in identifying differences in histone PTM landscape between parasite stages that could be related to the regulation of gene expression, so we focused our analysis on the intergenic regions upstream of gene start sites, which are likely to have promoter functions. We determined the abundance of each of the states across all promoter regions (1,000 bp upstream of the gene start site) in early schizont, merozoite, and early ring genomes. As parasites matured from early schizonts to merozoites, promoters already marked during the early schizont stage with H3K9ac and H3K4me3 appeared to gain H3K27ac as demonstrated by a reduction in ChromHMM state 4 and an increase in ChromHMM state 1 in merozoites (Fig. 2B, Additional file 2: Table S10). This may reflect a transition to a state of active gene expression in merozoites as H3K27ac is known to mark active promoter and enhancer regions [36, 37]. In keeping with this theory, genes with the highest expression in merozoites also had the highest enrichment of H3K27ac (Additional file 1: Fig. S6A, B). Following re-invasion, H3K9ac and H3K27ac levels appeared to decrease in gene promoters. These regions became mainly characterized by H3K4me3 and low levels of H3K9ac as seen in the increase in ChromHMM states 3 and 5 and decrease in ChromHMM states 1 and 4 in early rings compared to merozoites (Fig. 2B). This may reflect that the histone PTM landscape is altered following invasion to allow for the stage-specific expression of genes.

While visualizing our ChIP-seq data in IGV, we noticed that some intergenic regions contained a strong depletion of H3K4me3 flanked on either side by enrichment of H3K9ac and H3K27ac in merozoites (representative gene shown in Fig. 2C and average enrichment patterns shown in Fig. 2D). To confirm this finding, we generated a biological replicate for H3K4me3 in merozoites. The two H3K4me3 replicates were strongly correlated (Pearson correlation coefficient, r = 0.91). Importantly, the depletions we originally observed in intergenic regions were also present in the biological replicate (representative genes shown in Additional file 2: Fig. S7A–C). This pattern of H3K4me3 depletion flanked by enrichment of H3K9ac and H3K27ac corresponded with the ChromHMM state pattern 1–5–1 (merozoites, Fig. 2C). Those same regions in early schizonts and early rings showed a slight depletion of H3K4me3 that was not flanked by H3K9ac and H3K27ac and were mainly defined by ChromHMM state 2 (Fig. 2C). Using a custom Perl script, we identified all regions of the genome with H3K4me3 depletion marked by the 1–5–1 ChromHMM state pattern. After filtering for regions located in intergenic areas, we identified 66 regions with the H3K4me3 depletion pattern (Fig. 2D, Additional file 2: Table S11). Across these regions, the distribution of H3K9ac, H3K27ac, and H3K4me3 in merozoites differed dramatically from that in early schizonts and early rings where there was little fluctuation in all three marks.

The 66 areas with the H3K4me3 depletion pattern (i.e., ChromHMM 1–5–1 pattern) were located in intergenic regions upstream of a panel of 74 genes (Additional file 2: Table S11). These regions were found at varying distances from the genes and were not restricted to the 1000 bp upstream of gene start sites that we defined as promoter regions in our ChromHMM analysis (Fig. 2B). However, promoter regions and upstream regulatory elements in *Plasmodium* are poorly defined [28, 38, 39], meaning that while outside of the region we defined as promoters, these H3K4me3 depletions may still fall in regulatory regions.

To gain a better understanding of how the chromatin landscape of this gene panel compared to that of genes with known stage-specific expression patterns, we analyzed the frequency of ChromHMM states present in the promoter regions (defined as the 1,000 bp preceding the gene start site) of the H3K4me3 depletion genes (Additional file 2: Table S12) as well as a set of schizont-expressed genes (905 genes, Additional file 3: Table S13) and a set of ring-expressed genes (139 genes, Additional file 2: Table S14). As expected, promoters of schizont-expressed genes were enriched for high H3K9ac, high H3K27ac, and medium H3K4me3 in early schizonts (ChromHMM state 1, Fig. 2E, Additional file 2: Table S15). As early schizonts transitioned to merozoites and then early rings, schizont-expressed gene promoters lost this chromatin state, likely reflecting silencing of these genes following the schizont stage. Ring-expressed genes followed the opposite pattern, with promoters defined by ChromHMM state 1 increasing in frequency as parasites transitioned from early schizonts to early rings (Fig. 2E). This likely reflects the activation of ring-expressed gene promoters in the early ring stage. Interestingly, the promoters of the genes with the H3K4me3 depletion (i.e., ChomHMM 1–5–1 pattern) followed a similar pattern as the ring-expressed genes with the frequency of active promoters (characterized by ChromHMM state 1) increasing as parasites transitioned from early schizonts to early rings (Fig. 2E). Additionally, genes with H3K4me3 depletion were enriched for state 5 in merozoites, which was not observed for ring genes. These differences in the enrichment of state 5 between the promoters of the H3K4me3 depletion genes and ring-expressed genes demonstrates that the promoters of the genes with H3K4me3 depletion are subject to epigenetic remodeling not seen in the ring-expressed gene promoters.

Genes with H3K4me3 depletion (i.e., ChromHMM 1–5–1 pattern) in the upstream intergenic region had roles in numerous cellular processes, including egress and invasion, RNA-binding, and metabolism (based on a review of the literature, Fig. 2F, Additional file 2: Table S12). Exported proteins (n = 10) and proteins involved in export pathways (n = 12) were the two largest groups of genes. Protein export in early ring parasites is crucial to facilitate host cell remodeling following invasion [40–42]. Together, this suggests that the depletion of H3K4me3 in putative regulatory regions in merozoites is associated with a select group of genes that play important roles in establishing a productive infection in a newly invaded erythrocyte.

Given that the chromatin state of the H3K4me3 depletion gene promoters bore similarity to the promoters of ring-expressed genes, we next investigated the timing of expression of these genes. Using our RNA-seq data supplemented with the data from Wichers et al*.*, we assessed the expression of the genes with H3K4me3 depletion in the upstream intergenic regions in early schizonts (40 hpi), mid schizonts (44 hpi), late schizonts (48 hpi), merozoites, and mid rings (8 hpi). The majority of these genes were found cluster 3 from our differential gene expression analysis between early schizonts and merozoites (Fig. 1C). Generally, expression was highest in more mature merozoites (subpopulations 2 and 3, Fig. 2G, H, Additional file 2: Table S16, S17) and mid rings, consistent with roles for these genes in ring stage development following invasion. In keeping with this observation, nearly half of the genes with H3K4me3 depletion in the upstream intergenic region (n = 36) reached peak transcription at 1 hpi based on the data available from Painter et al*.* [20], which is a significant enrichment as compared to all genes (675/5373, *P* < 0.0001, Chi-squared test). Combined, these results indicate that genes with H3K4me3 depletion in the upstream intergenic region undergo active transcription in merozoites or immediately after invasion.

The uniform pattern of expression we observed for genes with H3K4me3 depletion (i.e., ChromHMM 1–5–1 pattern) in the upstream intergenic region suggests they are subjected to common regulatory processes. A protein binding to the DNA would result in depletion of H3K4me3 and lack of enrichment for H3K9ac and H3K27ac at the binding location in our ChIP samples much like the pattern we observed. To probe the possibility that a protein bound the region of H3K4me3 depletion, we performed a motif search in these regions (indicated by ChromHMM state 5, Fig. 2C) using MEME [43]. In 70 out of the 74 genes assessed, we identified a common motif (Fig. 2I). This motif bore similarity to the core DNA motif of AP2-EXP (CATGC) as well as the binding motif of AP2-11a [22, 44, 45]. Using Simple Enrichment Analysis [46], we observed that this motif was enriched in the H3K4me3 depletion regions (*P* = 0.0004) but not in randomly selected intergenic regions (*P* = 0.8), suggesting that these genes may be subjected to regulation by an AP2 transcription factor. Based on these results, we hypothesize that the observed histone PTM pattern in upstream intergenic regions is the result of a protein binding to this region and shielding the histone PTMs from antibody recognition during ChIP.

### H3K4me3 depletion in promoter regions is associated with PTEX and PTEX-related genes

Genes encoding proteins involved in export pathways (n = 12) was the largest group of genes within the set of genes with H3K4me3 depletion in upstream intergenic regions (Fig. 2F). Out of the 12 genes involved in protein export, 7 were part of or known to interact with the *Plasmodium* translocon of exported proteins (PTEX), a protein complex important for export of proteins from the parasitophorous vacuole into the host cell [41]. The promoters of three components of PTEX (PTEX150, HSP101, and PTEX88) had a similar histone PTM profile, with depletion of H3K4me3 flanked by H3K9ac and H3K27ac in the upstream intergenic regions. However, these regions were annotated primarily with a 1 – 5 – 2 – 5 – 1 ChromHMM state pattern and so they were not identified in our original analysis of 1 – 5 – 1 ChromHMM regions (Additional file 1: Fig. S8A–C). Like the other genes with H3K4me3 depletion in the upstream intergenic regions, these three genes were also expressed at higher levels in merozoites and early rings (Additional file 1: Fig. S8D–F, Additional file 2: Table S3, S4). Additionally, 9 out of the 10 PTEX-associated genes (including PTEX150, PTEX88, and HSP101) contained a common motif in the H3K4me3 depletion region (Fig. 2J) suggesting potential regulation by a common transcription factor; however, this motif does not match any known *Plasmodium* transcription factor motifs.

### Stage-specific depletion of H3K4me3 in intergenic regions is seen in both hepatic and erythrocytic merozoites

Liver stage *Plasmodium* development culminates in the formation of hepatic merozoites that will invade erythrocytes to initiate a blood stage infection [5]. The processes of egress and invasion that occur during the parasite transition from the liver to the blood have similarities to those that occur as parasites transition between erythrocytes, thus gene expression in hepatic merozoites and erythrocytic merozoites may be subjected to similar regulatory processes [47, 48]. To probe this possibility, we performed ChIP-seq for H3K4me3 and H3K9ac in *Plasmodium berghei* hepatic merozoites (68–75 hpi). Due to the technical challenges of producing hepatic *P. falciparum* merozoites, we utilized *P. berghei* for this analysis. Merozoites were prepared by collecting merosomes and releasing hepatic merozoites by saponin treatment. Due to limited input material, we only assessed H3K4me3 and H3K9ac.

**Fig. 3:**
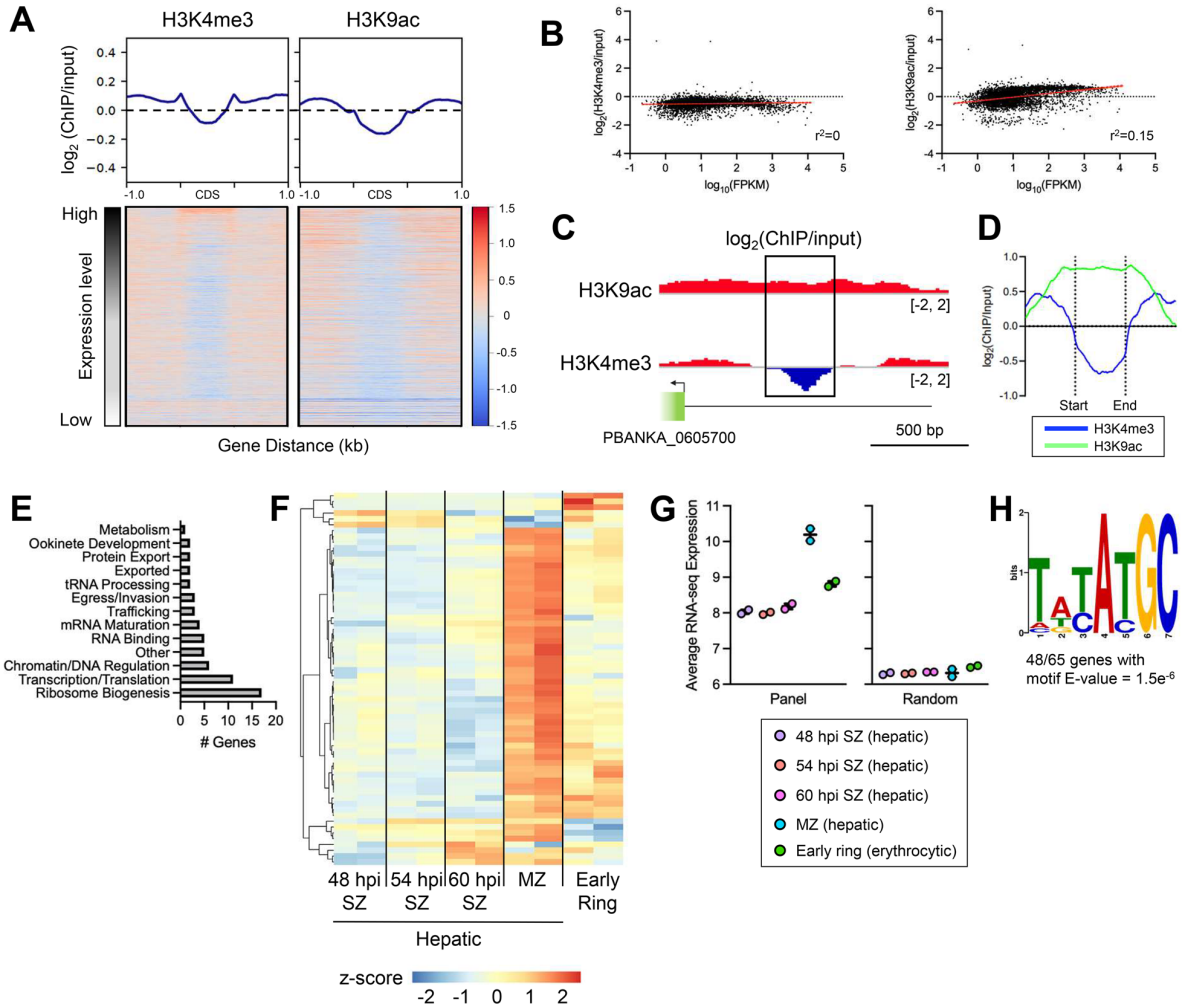
H3K4me3 depletion region upstream of genes in *P. berghei* hepatic merozoites. **A** Distribution of H3K4me3 and H3K9ac in hepatic merozoites (68–75 hpi) shown as log_2_(ChIP/input) across all coding sequences (CDS) in the genome, 1.0 kb upstream, and 0.5 kb downstream of the genes. All genes (n = 5,042) were scaled to the same size by computeMatrix. Enrichment of each mark for individual genes is depicted in the heatmap. Genes are sorted by expression level in hepatic merozoites. **B** Gene expression (log_10_FPKM) plotted against H3K4me3 or H3K9ac enrichment (log_2_(ChIP/input)) in the promoter region (1,000 bp upstream of the gene ATG). Line of best fit as determined by linear regression analysis is depicted by red line. The 95% confidence interval of the line of best fit is indicated by dashed red lines. **C** H3K9ac and H3K4me3 ChIP tracks depicting a region of H3K4me3 depletion upstream of PBANKA_0605700. Gene coding sequence is indicated by the green box. The black rectangle indicates the region of H3K4me3 depletion. Scale bar depicting 500 bp is shown. **D** Average enrichment of H3K9ac and H3K4me3 in hepatic merozoites depicted as log_2_(ChIP/input) across 58 regions with H3K4me3 depletion. These regions were scaled to the same size by computeMatrix, and the enrichment 1.0 kb upstream and downstream of the regions is displayed. **E** Functions associated with genes belonging to the H3K4me3 depletion panel. Functions were assigned based on a literature review and can be found in Additional file 2: Table S19. Genes annotated as ‘conserved with unknown function’ (n = 10) in PlasmoDB are not shown but are included in all analyses. **F** Heatmap depicting DESeq2 normalized gene expression of H3K4me3 depletion panel genes (n = 64) in 48 hpi hepatic schizonts (48 hpi SZ, n = 2), 54 hpi hepatic schizonts (54 hpi SZ; n = 2), 60 hpi hepatic schizonts (60 hpi SZ; n = 2), hepatic merozoites/DCs (MZ; 69 hpi, n = 2), and erythrocytic early rings (4 hpi, n = 2). **G** Boxplots depicting the average gene expression of genes with H3K4me3 depletion (Panel, n = 64) and a set of randomly selected genes (Random, n = 147). Gene expression did not differ between time points for the panel of genes with H3K4me3 depletion (Kruskal–Wallis test). **H)** DNA motif in the H3K4me3 depletion regions

In *P. berghei* hepatic merozoites, H3K4me3 and H3K9ac were enriched in intergenic regions and depleted in gene bodies (Fig. 3A). Similar to our observations in *P. falciparum*, H3K4me3 in hepatic merozoites also had modest peaks associated with the gene start and end sites (Fig. 3A). However, in contrast to what we saw in *P. falciparum,* we also observed enrichment of these marks in the intergenic regions immediately flanking coding regions.

Given the differences in distribution between these histone PTMs in hepatic and erythrocytic stages, we sought to characterize the relationship between these marks and gene expression in hepatic merozoites. A recent study by Caldelari et al*.* analyzed RNA-seq data from hepatic schizonts (48, 54, and 60 hpi), merosomes/detached cells (DCs) containing hepatic merozoites (69 hpi), and erythrocytic early rings (4 hpi) from *P. berghei* [49]*.* Using this data set, we first compared the level of H3K4me3 or H3K9ac to the level of gene expression in hepatic merozoites from the 69 hpi merosomes/DCs. In keeping with observations in *P. falciparum*, H3K4me3 in the promoter region did not appear to be associated with gene expression, while H3K9ac in the promoter had a positive correlation with gene expression, comparable to what we saw in *P. falciparum* (Fig. 3A, B Additional file 1: Fig. S6A, B). These results suggest that the abundance of H3K9ac and H3K4me3 in promoter regions in hepatic merozoites has a similar relationship to gene expression as in erythrocytic merozoites.

Upon further visual inspection of the hepatic merozoite promoter regions, we noticed that some genes harbored a region of H3K4me3 depletion, albeit with enrichment in H3K9ac (Fig. 3C). To probe whether these H3K4me3 depletions in hepatic merozoites were associated with similar gene expression signatures as the depletion regions we observed in erythrocytic merozoites, we set out to further characterize this pattern in hepatic merozoites. Using a custom Perl script, we identified 58 regions of at least 250 bp with at least a threefold depletion of H3K4me3 compared to H3K9ac in intergenic regions (Fig. 3D, Additional file 2: Table S18). These regions corresponded to 65 genes that were largely involved in ribosome biogenesis, transcription, translation, and chromatin regulation (Fig. 3E, Additional file 2: Table S19). Histone 3.3 (PF3D7_0617900, PBANKA_1117100), PV1 (PF3D7_1129100, PBANKA_0919100), multidrug resistance protein 1 (MDR1; PF3D7_0523000, PBANKA_1237800), bax inhibitor 1 (PF3D7_1248500, PBANKA_1461400), and tRNA arginine (PF3D7_0529600, PBANKA_1244051) were present in both the groups of genes from erythrocytic and hepatic merozoites with depletion of H3K4me3. Additionally, polyadenylate-binding protein (PABP) 1 appears in the erythrocytic merozoite gene panel, while PABP2 is part of the hepatic merozoite gene panel. Together, these six genes represented a significant overlap between the two gene lists (*P* < 0.001; hypergeometric distribution test).

Using the RNA-seq data set of *P. berghei* 48 hpi hepatic schizonts, 54 hpi hepatic schizonts, 60 hpi hepatic schizonts, hepatic merozoites/DCs (69 hpi), and erythrocytic early rings (4 hpi) from Caldelari et al. [49], we investigated the expression of genes with H3K4me3 depletion as parasites transition from the liver stage to the IDC. Like the genes we identified in erythrocytic merozoites with H3K4me3 depletion, the hepatic merozoite H3K4me3 depletion genes were most highly expressed in merosomes/DCs (69 hpi) with some genes remaining expressed following invasion in erythrocytic early rings (4 hpi) (Fig. 3F, G Additional file 2: Tables S20, S21). Additionally, 48 out of 65 of these genes contained a common DNA motif in the H3K4me3 depletion region with similarity to the DNA motif we identified in *P. falciparum* erythrocytic merozoites (Fig. 3H). These results suggest that gene expression in hepatic merozoites and erythrocytic merozoites may, in part, be subject to the same regulatory processes.

### H3K4me2 marks virulence genes in erythrocytic merozoites

**Fig. 4:**
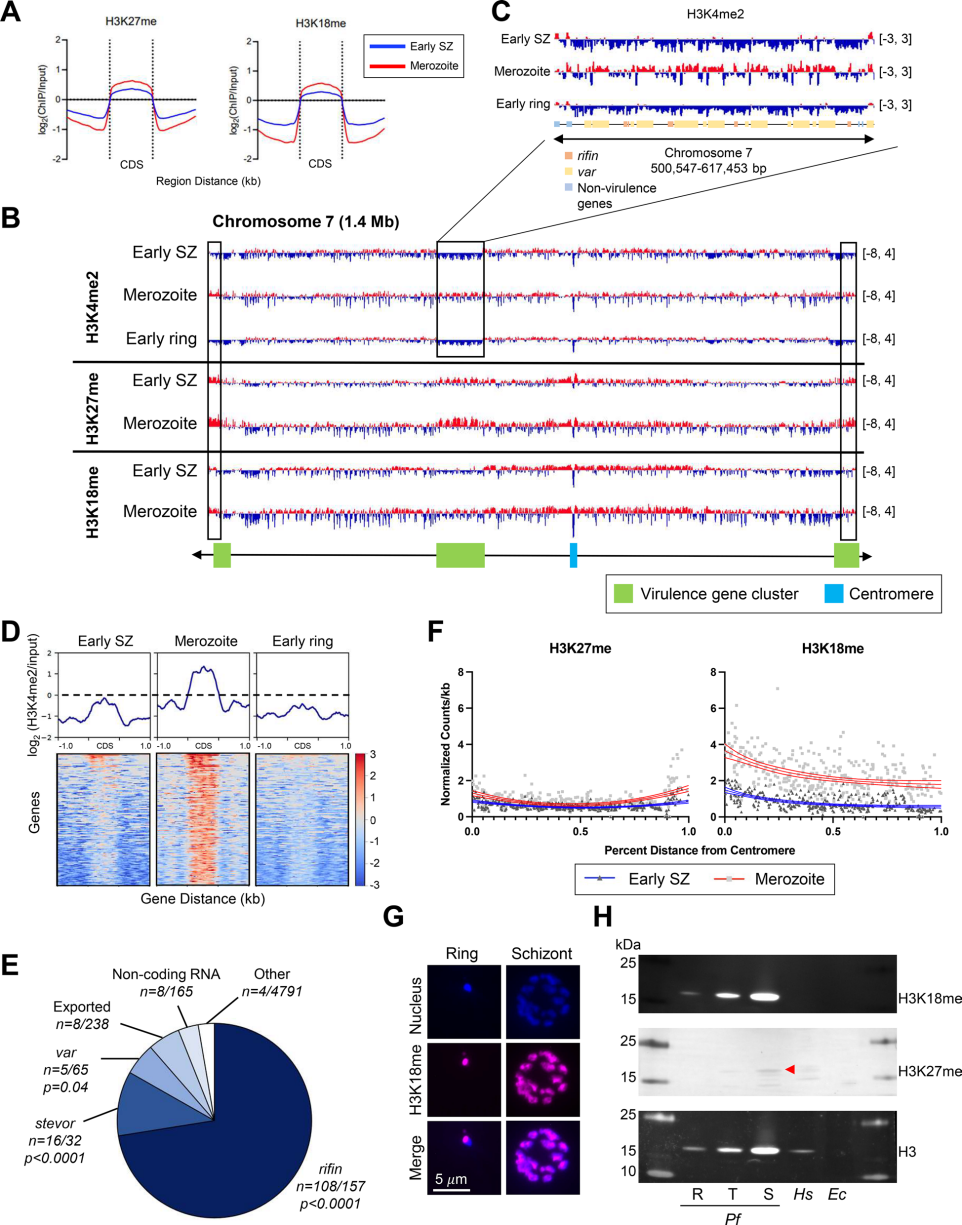
Methylation marks associated with specific genome features.** A** Log_2_(ChIP/input) enrichment of H3K27me and H3K18me across the coding region (CDS) of all genes in the genome (n = 5,602) scaled to the same size by computeMatrix. The 1.0 kb region upstream and downstream of the CDS is also depicted. **B** H3K4me2, H3K27me, and H3K18me ChIP tracks depicting log_2_(ChIP/input) for a representative chromosome. Regions where histone PTMs have higher enrichment in merozoites than other stages are indicated by black rectangles. Data range of ChIP tracks is indicated in brackets. **C** H3K4me2 log_2_(ChIP/input) tracks for early schizonts (40 hpi), merozoites, and early rings (4 hpi) highlighting the internal virulence gene cluster on chromosome 7. Data range for ChIP tracks is indicated in brackets. **D** H3K4me2 enrichment in merozoites compared to early schizonts (n = 178) shown across the coding sequence (CDS) and 1.0 kb up- and downstream of the gene. Genes were scaled to the same size by computeMatrix. **E** Types of genes with H3K4me2 enrichment in merozoites compared to early schizonts. Overrepresentation *of var*, *rifin, stevor,* exported proteins, and non-coding RNA was tested for statistical significance with Chi-squared test. **F)** Distribution of H3K27me and H3K18me across chromosome 7 (a representative chromosome). The level of histone marks is shown as counts per gene length (kb) normalized to input values. The two arms of the chromosome are scaled and depicted as the proportion of the distance from centromere with the centromere located at 0 and both telomeres located at 1. Line of best fit as determined by nonlinear regression analysis is indicated by solid blue (schizonts, 40 hpi) and red (merozoites) lines. The 95% confidence interval of the line of best fit is indicated by the outer red or blue lines. **G)** Immunofluorescence images of H3K18me staining in schizonts and rings. **H)** Western blots of H3K18me and H3K27me in nuclear fractions of *P. falciparum* rings (R), trophozoites (T), and schizonts (S). The red triangle indicates the H3K27me band. Total cell lysates of the human (*Hs*) cell line Expi293F and *E. coli* cells (*Ec*) were included as controls

In addition to characterizing marks associated with transcriptional activation, we investigated four less studied histone PTMs that are known to be dynamically distributed throughout the *Plasmodium* IDC: H3K4me, H3K4me2, H3K27me, and H3K18me [31]. Unlike the histone PTMs associated with active gene expression, these four marks were almost exclusively located inside gene bodies in *P. falciparum* early schizonts (40 hpi), merozoites, and early rings (4 hpi) (Fig. 4A, Additional file 1: Fig. S5). Additionally, these marks were present at the highest levels in merozoites and the lowest levels in early rings (H3K4me and H3K4me2, Additional file 1: Fig. S5) or early schizonts (H3K18me and H3K27me, Fig. 4A).

In general, H3K4me2 is associated with ‘permissive’ chromatin that can be either active or potentially active [50]. Indeed, in *Plasmodium*, H3K4me2 is found enriched in regions flanking the gene start site of active *var* genes and appears to be a general feature of active genes [51]. We observed that H3K4me2 marked the subtelomeric regions and internal virulence gene clusters in merozoites, whereas this PTM was absent in these regions in early schizonts and early rings (Fig. 4B, C Additional file 1: Fig. S9). We identified a set of genes (n = 150) that showed enrichment of H3K4me2 in merozoites compared to early schizonts and early rings (Fig. 4D, Additional file 3: Table S22). This panel of genes was enriched for genes from the *rifin*, *stevor*, and *var* gene families (Fig. 4E) in keeping with the localization of H3K4me2 to subtelomeric regions and internal virulence gene clusters in merozoites. As expected, this set of genes was expressed at a substantially lower level than a set of randomly selected *P. falciparum* genes throughout the IDC (Additional file 1: Fig. S10, Additional file 2: Tables S23–S26). As parasites mature from early schizonts to early rings, this set of genes underwent a slight increase in expression (Additional file 1: Fig. S10). While we observed a clear enrichment of H3K4me2 in merozoites compared to early schizonts and early rings, almost exclusively in virulence genes, further investigation is required to understand the functional impact of this mark in merozoites.

### H3K27me and H3K18me are associated with centromeric and subtelomeric regions

The final two histone PTMs we assessed, H3K27me and H3K18me, have been shown to be present in *P. falciparum* by mass spectrometry [31], but their distribution across the genome is unknown. In other organisms, H3K27me is associated with repressed genes and heterochromatin [52]. In *Theileria* parasites, which are closely related to *Plasmodium*, H3K18me is associated with repressed genes [32].

In *P. falciparum*, the distributions of H3K27me and H3K18me correlated strongly with each other within and between stages (Additional file 1: Fig. S4, r = 0.65–0.97). These marks also had the weakest correlations with the majority of activating histone PTMs (H3K4me3, H3K9ac, and H3K27ac), regardless of stage (Additional file 1: Fig. S4, r = – 0.34–0.45). In early schizonts and merozoites, the abundance of H3K27me increased with proximity to the centromere and subtelomeric regions, with slightly higher levels of this mark in merozoites (Figs. 4B, F, Additional file 1: Figs. S9, S11). H3K18me, while found in the subtelomeric regions, was primarily enriched with proximity to the centromere (Figs. 4B, F, Additional file 1: Figs. S9, S12). Both of these marks also associated with internal virulence gene clusters (Fig. 4B, Additional file 1: Fig. S9).

To probe whether H3K18me and H3K27me were present at the ring stage, we performed immunofluorescence assays (IFA) on ring stage and schizont stage parasites. Staining with the H3K27me antibody was unsuccessful in both schizonts and rings, suggesting that this antibody was incompatible with IFA. H3K18me was detected in both rings and schizonts, and this signal was localized within the nucleus (Fig. 4G, Additional file 1: Fig. S13, 14). For several parasites imaged, the H3K18me staining appeared as a single point localized to the edge of the nucleus, potentially consistent with enrichment of this mark around the centromeres. To further investigate the presence of these marks during the IDC, we performed western blot analysis of H3K18me and H3K27me at the three main developmental stages: rings (12–16 hpi), trophozoites (32–36 hpi) and schizonts (40–44 hpi) (Fig. 4H, Additional file 1: Fig. S15). H3K18me was detected in rings, trophozoites, and schizonts and its signal seemed directly proportional to the amount of histone H3 present in the cell. The signal for H3K27me was weak, but detectable in trophozoites and schizonts. The enrichment of H3K27me and H3K18me in subtelomeric regions and the centromere coupled with the similarity in enrichment across schizonts, merozoites, and rings may suggest that these marks are largely structural with potential roles in regulating chromatin organization during the schizont-to-ring transition.

## Discussion

The transition of *Plasmodium* parasites from one cell to the next within the human host has been understudied as compared to the main intracellular developmental stages. In this study, we aimed to gain insight into the regulatory processes that underlie this crucial phase of the *Plasmodium* life cycle. To this end, we assessed changes in gene expression and epigenetic landscape during the schizont-to-ring transition by integrating RNA-seq and histone PTM ChIP-seq data from populations of *P. falciparum* blood stage schizonts, merozoites, and rings, as well as *P. berghei* hepatic merozoites. By focusing on the changes in gene expression and histone PTM distribution between schizonts and merozoites and between merozoites and rings, we have been able to identify unique histone PTM remodeling related to changes in gene expression as parasites undergo egress and reinvasion.

It has long been known that gene expression during the *P. falciparum* asexual blood stage occurs in a cascade-like pattern [7–9]. Additionally, developmental transitions during the IDC are associated with bursts of transcription and mRNA transcript stabilization [20]. Our data suggest that a similar burst of transcription occurs during the final stages of merozoite development. For a panel of genes, we observed upregulation in merozoites as compared to schizonts. This change in gene expression was captured in both our data set and a previously published, independently generated data set by Wichers et al. [33], adding confidence that these observations are truly reflective of different merozoite developmental stages and not merely the result of differences in sample preparation or stochastic variation in gene expression. It is not immediately clear why upregulation of this gene set was only captured in one out of three merozoite replicates (Fig. 1C, merozoite subpopulation 3), despite identical treatment of parasite cultures. It should be mentioned that the samples that we refer to as merozoites are fully segmented schizonts that are mechanically forced to rupture. E64-arrested merozoites are viable but short-lived, and their invasion capacity is temperature dependent [6]. It is possible that small differences in timing or conditions of sample collection during the final stages of merozoite development are responsible for these striking differences in gene expression. A burst of transcription was previously observed in very young ring stage parasites (1 hpi), resulting in the largest number of nascent transcripts at any point in the IDC [20]. As such, it is also possible that the burst of transcription we observed in E64-arrested merozoites does not happen in merozoites that have been allowed to egress naturally and normally occurs immediately after invasion.

Our analysis uncovered a unique histone PTM profile in *P. falciparum* erythrocytic merozoites characterized by depletion of H3K4me3 with flanking enrichment of H3K9ac and H3K27ac upstream of gene start sites. These genes were all expressed at the highest level in erythrocytic merozoites and primarily have roles in protein export and host cell remodeling (exported proteins). H3K4me3 is generally considered an activating histone PTM, and it is therefore surprising to observe that H3K4me3 depletion correlated with gene expression. However, the combined lack of H3K4me3, H3K9ac, and H3K27ac may point to occlusion of this region from antibody binding and pulldown during ChIP by DNA and chromatin binding proteins, and does not necessarily signify an absence of these marks. It is important to note that our ChIP-seq and RNA-seq samples were not matched samples from the same culture. This limits our ability to directly connect observations in the bulk ChIP-seq data to the different merozoite gene expression profiles. However, all the genes in the H3K4me3 depletion panels (*P. falciparum* erythrocytic and *P. berghei* hepatic) were highly upregulated at the merozoite stage, suggesting that this histone PTM profile is reflective of expression of a select group of genes. Several genes associated with or comprising the *Plasmodium* translocon of exported proteins (PTEX) were represented in the erythrocytic merozoite panel, including four out of the five main components of PTEX (PTEX150, PTEX88, HSP101, EXP2) as well as several accessory proteins known to interact with PTEX (including P113, PV1, EXP3) [53]. Previous work has demonstrated that several main components of PTEX (PTEX150, HSP101, and EXP2) are synthesized in the schizont stage and stored in dense granules until after invasion [54]. Additionally, a large proportion of genes with functions in the ring stage are transcribed in the schizont stage prior to egress to be translated in rings [55]. In line with these results, our data support that rapid transcriptional changes occur as parasites proceed through schizogony to egress. The H3K4me3 depletion histone PTM profile likely represents genes that are transcribed during late schizogony and are essential for the establishment of a productive infection during the earliest parasite stages following invasion.

Analysis of the histone PTM landscape in *P. berghei* hepatic merozoites uncovered a panel of merozoite-expressed genes with depletion of H3K4me3 in the upstream intergenic region and enrichment for a DNA motif that was similar to the one found in the *P. falciparum* erythrocytic merozoite gene panel. However, in contrast to the erythrocytic genes with H3K4me3 depletion, this depletion in hepatic merozoites was not accompanied by a defined H3K9ac pattern. Another difference between the two sets of genes was that the genes in the *P. berghei* hepatic merozoites were largely involved in ribosome biogenesis, transcription, and translation, not protein export. Differences between the genes identified in hepatic merozoites and erythrocytic merozoites may be due to biological differences between the liver and blood stages. Notably, hepatic merozoites are gradually released from hepatocytes inside merosomes in vivo, which subsequently rupture in pulmonary capillaries [56]. Hepatic merozoites therefore exist for hours before free merozoites are released into the blood stream. In contrast, erythrocytic merozoites will invade a new erythrocyte within seconds to minutes after egress [6]. Differences in developmental timing may underly the differences we observed between the genes with unique histone PTM remodeling in hepatic and erythrocytic merozoites. As merozoites develop, the genes marked by this histone PTM profile may shift from ones primarily related to translational processes to those involved in protein export. Interestingly, in hepatic merozoites, EXP2 did have a depletion of H3K4me3 flanked by H3K9ac enrichment upstream of the gene start site, however, the difference between H3K4me3 and H3K9ac was below the cutoff used to identify genes with H3K4me3 depletion. Expression of this gene is also significantly enriched in hepatic merozoites compared to hepatic schizonts, similar to what we observed for other genes with the H3K4me3 depletion. Combined, these results suggest that differences between the genes identified in hepatic merozoites and erythrocytic merozoites are likely reflective of differences between liver and blood stage development, as previously observed between hepatic and erythrocytic merozoites in *P. berghei* [49]. However, the presence of similar histone PTM remodeling and a similar motif in the depletion region suggests that there are similar processes of gene regulation that occur during both liver stage and IDC egress and that may regulate the expression of genes required for early ring stage development following invasion. Further research is needed to confirm whether the differences we observed here are reflective of differences between the liver and blood stage, or between *P. falciparum* and *P. berghei*.

The DNA motifs identified in the H3K4me3 depletion regions in both erythrocytic merozoites and hepatic merozoites resembled the core AP2-EPX motif. In both the erythrocytic samples and the hepatic samples, AP2-EXP (*P. berghei* ortholog AP2-Sp) was upregulated in merozoites, leaving the possibility that AP2-EXP is subject to a positive feedback loop in the late stages of the IDC. Our data thus suggest that AP2-EXP may have roles in late schizogony and early rings. In apparent contrast to this, a recent study by Russell et al*.* demonstrated that AP2-EXP is only required for parasite development from 30–46 hpi, as this was the only timeframe where parasite growth could be arrested by a AP2-EXP-specific inhibitor [4]. This inhibitor was identified by molecular docking into the DNA binding groove of the crystal structure of AP2-EXP and is likely optimized for binding to the AP2-EXP homodimer. However, AP2 transcription factors are known to form heterodimers with other transcription factors [57]. Formation of a heterodimer containing AP2-EXP and another AP2 transcription factor in late stage schizonts could explain why the parasite lost sensitivity to inhibitors after 46 hpi but appears to have functional roles after this point. Differences between our binding motif and the published AP2-EXP motif could also be explained by heterodimer formation. Alternatively, this DNA motif may be bound by a different protein factor in merozoites altogether. A recent study by Shang et al*.* demonstrated that the motif CATG is associated with several AP2 transcription factors [45]. AP2 transcription factors may also recognize different motifs at different stages of parasite development [45], leaving the possibility that another transcription factor binds instead of or in conjunction with AP2-EXP at the motifs identified in our analysis.

We also characterized, to our knowledge for the first time, the distribution of H3K27me and H3K18me during the *P. falciparum* IDC. Both of these marks were enriched in coding sequences and did not correlate with known activating marks, suggesting they are not involved in transcriptional activation. H3K27me is enriched at the centromeres and the telomeres while H3K18me is enriched near centromeres. *Plasmodium* chromosome arrangement is highly ordered with clustering of centromeres near the perimeter of the nucleus [58, 59]. The enrichment of these methylation marks near the centromeres may indicate a role for H3K27me and H3K18me in maintaining chromosome organization during schizogony or possibly throughout the entire cell cycle.

Due to the difficulty of collecting sufficient amounts of parasites for ChIP-seq, the majority of our histone PTM analysis is from a single sample. However, we observed strong correlation between marks with similar deposition patterns as well as strong correlations between marks at different stages. For example, H3K18me and H3K27me deposition were strongly correlated between the schizont and merozoite stages (Pearson's r > 0.95, Additional file 1: Fig. S4). H3K9ac and H3K27ac are known to have similar distributions, and in our dataset, these two marks correlated well with each other in each stage (Additional file 1: Fig. S16). The strong correlations of histone marks with each other and between stages provides confidence in the overall quality of our data.

Overall, in this study we have shown that extensive changes in gene expression and histone landscape occur in *Plasmodium* parasites during the schizont-to-ring transition, highlighting the dynamic nature of this process. During merozoite formation, the promoters of a subset of genes with roles in early ring stage development undergo unique histone PTM remodeling linked to high transcriptional activity driven by a common DNA binding factor. In addition, we identified striking similarities in epigenetic and transcriptional alterations between liver stage and blood stage merozoites. The importance of merozoite formation for continued infection and the dynamic remodeling of the transcriptional program during egress and invasion highlight the potential of this stage as a target for novel anti-malarial approaches that may block the formation of infective merozoites in both the liver and blood stages.

## Methods

### Parasite culture and synchronization

*P. falciparum* 3D7 strain parasites were cultured as described previously [60], with modifications (Additional file 3). In short, parasites were cultured in human AB^+^ erythrocytes (Interstate Blood Bank, Memphis, TN, USA) washed as described in the Additional file 3 at 1–10% parasitemia in complete culture medium (5% hematocrit). Complete culture medium consisted of RPMI 1640 medium (Gibco #32404014) supplemented with gentamicin (45 µg/ml final concentration (f.c.); Gibco #15710064), HEPES (40 mM f.c.; Fisher #BP3101), NaHCO_3_ (1.9 mg/ml f.c.; Sigma #SX03201), NaOH (2.7 mM f.c.; Fisher #SS266-1), hypoxanthine (17 µg/ml f.c.; Alfa Aesar #A11481-06), L-glutamine (2.1 mM f.c.; Corning #25005Cl), D-glucose (2.1 mg/ml f.c.; Fisher #D16-1), and 10% (vol/vol) human AB^+^ serum (Valley Biomedical #HP1022). Parasites were cultured at 37 °C in an atmosphere of 5% O_2_, 5% CO_2_, and 90% N_2_ (Heracell VIOS 160i TriGas CO_2_ incubator, Thermo #51030408).

*P. falciparum* parasites were synchronized to the ring stage by treatment with 5% D-Sorbitol (Fisher #S459-500) as described previously [61], with modifications (Additional file 3). Briefly, cultures containing 3 – 10% ring-stage parasites were centrifuged at 250 × *g* for 5 min. at RT. The supernatant was discarded, and the pellet was resuspended in 10 volumes of 5% D-sorbitol in Milli-Q water and vortexed for 30 s. to disrupt erythrocytes. Vortexed erythrocytes were incubated for 8 min. at 37 °C then centrifuged at 250 × *g* for 5 min. at RT. The resulting erythrocyte pellet was washed with 5 volumes of complete culture medium and then centrifuged at 250 × *g* for 5 min. at RT. Washed erythrocytes were resuspended in complete culture medium to obtain a hematocrit of 5% and cultured as described above. To obtain tightly synchronized parasites, sorbitol treatments were performed twice, 14 h apart.

### Schizont isolation

Infected erythrocytes containing *P. falciparum* parasites in the late-trophozoite and schizont stages (40 hpi) were isolated from culture by magnetic separation as previously described [6, 62], with modifications (Additional file 3). Briefly, late-stage parasites were separated from uninfected and ring-infected erythrocytes with a SuperMACS II Separator (Miltenyi #130-044-104). The magnet was assembled with a D column (Miltenyi #130–041-201) according to manufacturer’s instructions. The column was equilibrated with 200 ml incomplete medium (complete culture medium without human serum). An additional 50 ml incomplete medium was added to the column through the side syringe to remove air bubbles possibly remaining in the column matrix. A 22 G needle (BD #305155) was attached to the stopcock to serve as a flow restrictor. For safety purposes the plastic protective sheath remained on the needle after cutting the end to allow flow of the liquid without exposing the tip of the needle. Approximately 100–200 ml of synchronized parasite culture (5–10% parasitemia, 5% hematocrit) 24–27 h following the second sorbitol treatment (majority of parasites in the early segmented schizont stage, 4–6 nuclei visible by Giemsa staining) were used for isolation. After passing the parasite culture through the column, the column was washed with incomplete medium until the flowthrough was clear. Next, the column was washed with a total of 150 ml incomplete medium. Erythrocytes containing late-stage parasites with high paramagnetic hemozoin levels are retained in the column matrix while attached to the magnet [63] allowing for separation of late-stage parasites from uninfected erythrocytes and early-stage parasites. The column was removed from the magnet and 60 ml incomplete medium was used to elute the erythrocytes containing schizonts from the column matrix. Cells were pelleted by centrifugation at 250 × *g* for 5 min. at RT and then washed with 45 ml cold PBS and pelleted by centrifugation at 250 × *g* for 5 min. at 4 °C. Cells were incubated for 10 min. on ice with 5 times the pellet volume 0.15% saponin (Millipore Sigma #558255100GM) in Milli-Q water to release schizonts by lysing erythrocytes. Released schizonts were pelleted by centrifugation at 4,000 × *g* for 10 min. at 4 °C. The resulting pellet was washed with cold PBS until the supernatant was clear following centrifugation (typically after 2 centrifugations). When collected for ChIP-seq, schizonts were washed once more with 1 ml cold PBS and then crosslinked with 1% paraformaldehyde in nuclease-free water for 10 min. at 37 °C. Glycine (Acros #120072500) was added to a final concentration of 0.125 M to quench the crosslinking reaction, followed by a 5 min. incubation at 37 °C. Crosslinked schizonts were washed 3 times with PBS by resuspending in 1 ml PBS and pelleting by centrifugation at 4,000 × *g* for 5 min. at 4 °C. After the final wash, the supernatant was removed and the crosslinked schizont pellet was stored at − 70 °C until used for ChIP. Schizonts collected for RNA-seq were resuspended in TRI reagent (Zymo #R2050-1-200) and stored at −70 °C until RNA-seq library preparation. An estimated 6 × 10^8^ schizonts were collected per 25 ml of synchronized culture, and 3 × 10^8^ to 6 × 10^8^ schizonts were used to perform ChIP-seq for each antibody assessed or for RNA-seq.

### Merozoite isolation

Following synchronization by sorbitol treatments, infected erythrocytes containing schizonts (40 hpi) were isolated by magnetic separation as described above. After elution from the column, erythrocytes containing schizonts were pelleted by centrifugation at 250 × *g* for 5 min. at RT and were resuspended in 3 ml complete culture medium. Schizont-containing erythrocytes were incubated with 10 µM (f.c.) E64 protease inhibitor (Sigma #324890-1MG) for 8 h at 37 °C to allow for parasites to develop into fully segmented schizonts while preventing egress from the erythrocytes. The cells were then pelleted by centrifugation at 1,900 × *g* for 8 min. at RT and the supernatant containing E64 was removed. A thin smear from the pellet was Giemsa (Sigma #GS1284L) stained and merozoite yield was assessed by counting the number of mature, membrane enclosed merozoites present. The pellet was resuspended in 4 ml incomplete medium. Merozoites were released from the erythrocytes by passing them through a 1.2-µm syringe filter (Pall #4190) and were subsequently pelleted by centrifugation at 4,000 × *g* for 10 min. at RT. On average, 5 × 10^7^ merozoites were collected per 25 ml of synchronized culture, and an estimated 5 × 10^7^ to 9 × 10^7^ merozoites were used to perform ChIP-seq for each antibody assessed or for RNA-seq. Merozoites collected for RNA-seq were resuspended in TRI reagent and stored at −70 °C until RNA-seq library preparation. When collected for ChIP-seq, merozoites were resuspended in PBS and crosslinked as described above. Crosslinked merozoites were stored at −70 °C until used for ChIP. See Additional file 3 for complete isolation protocols.

### Ring isolation

Cultures were synchronized to the ring stage by two sorbitol treatments (as described above) then cultured for 25 h in the presence of heparin (20 U/ml; Sigma Aldrich #H3149-100KU) to prevent reinvasion of erythrocytes. Once the majority of parasites had developed to the late-schizont stage (8–16 nuclei visible as assessed by Giemsa stain), the culture was centrifuged at 250 × *g* for 5 min. at RT to pellet the erythrocytes. The supernatant containing heparin was removed, and the erythrocytes resuspended in complete culture medium (5% hematocrit). The culture was incubated under normal growth conditions (37 °C in an atmosphere of 5% O_2_, 5% CO_2_, and 90% N_2_) for 4 h to allow reinvasion. Following the reinvasion window, mature parasites were removed from the culture by treatment with 5% sorbitol (as described above). To ensure complete removal of mature parasites, remaining trophozoite- and schizont-infected erythrocytes were removed by magnetic separation using a MidiMACS separator (Miltenyi #130–042-501). The magnet was assembled with a MACS LS column (Miltenyi #130–042-401) according to manufacturer’s instructions. The column was equilibrated with 5 ml incomplete medium. Cultures were centrifuged at 250 × *g* for 5 min. at RT to pellet erythrocytes. The pellet was resuspended in complete medium to 25% hematocrit and passed through the column. The flowthrough was collected then passed back through the column to further remove mature parasite infected erythrocytes. The column was washed with 2.75 ml complete medium. Uninfected and ring-stage infected erythrocytes from the magnet flowthrough were pelleted by centrifugation at 250 × *g* for 5 min. at RT. The erythrocytes were then washed with 45 ml cold PBS and pelleted by centrifugation at 250 × *g* for 5 min. at 4 °C. Ring-stage (4 hpi) parasites were released from erythrocytes by saponin treatment and crosslinked for ChIP-seq as described above. An estimated 1.25 × 10^7^ rings were collected per 25 ml of synchronized culture, and roughly 2.5 × 10^7^ rings were used to perform ChIP-seq for each antibody assessed or for RNA-seq. Crosslinked rings were stored at −70 °C until used for ChIP. See Additional file 3 for isolation protocols.

### *Plasmodium berghei* hepatic merozoite isolation

HepG2 human hepatoma cells (authenticated via STR profiling by ATCC) were cultured according to standard practices in complete DMEM medium (Dulbecco’s Modified Eagle Medium (Gibco #10313021) supplemented with 10% (v/v) fetal bovine serum (Gibco #A38402-02), 1X GlutaMAX (Gibco 35050-061), 100 U/ml penicillin (Gibco #15140122), and 100 μg/ml streptomycin (Gibco #15140122)) at 37 °C and 5% CO_2_. The day prior to infection, HepG2 cells were seeded at 75,000 cells per well in a volume of 500 µl of complete DMEM in a 24-well plate. The day of infection, salivary glands containing *P. berghei* sporozoites were dissected from live mosquitos (obtained from the University of Georgia insectary, USA) and ground with a small pestle to release sporozoites. The released sporozoite solution was passed through a 40 µm filter and counted using a hemocytometer. Sporozoites were then diluted to 40,000 sporozoites per 300 µl of infection medium (iDMEM: complete DMEM supplemented with 100 U/ml penicillin, 50 μg/ml streptomycin, and 150 μg/ml neomycin (Gibco 15640-055) and 50 µg/ml kanamycin (Corning #30–006-CF), and 7.5 µg/ml of amphotericin B (Gibco #15290-018)). Culture medium was removed from the wells containing the adherent HepG2 cells and 300 µl of the sporozoite dilution was added to each well. The plate was centrifuged at 2,000 × *g* for 5 min. at RT, then returned to 37 °C and 5% CO_2_. Two hours post infection, all medium was removed and replaced with 1 ml of iDMEM in each well, with a subsequent iDMEM replacement at 48 hpi. Detached HepG2 cells and merosomes were harvested between 68 – 75 hpi. The iDMEM from each well was collected into a 15-ml tube, and the well rinsed with an additional 500 µl of iDMEM, which was then added to the collection tube. Merosomes were pelleted by centrifugation at 4,000 × *g* for 5 min. at RT, and all but 100 µl of the iDMEM supernatant was removed. The merosome pellet was resuspended in the remaining supernatant and transferred to a 1.6-ml tube. PBS was added to the merosomes to a final volume of 1 ml; 10 µl of 1% saponin was then added and incubated for 2 min to release the merozoites from HepG2 membranes. Merozoites then were pelleted by centrifugation at 4000 × *g* for 4 min. at RT. Supernatant was removed as described above, and PBS was added to the resuspended merozoites to a final volume of 1 ml. Merozoites were then crosslinked for ChIP-seq as described above (Schizont isolation section). Crosslinked merozoites were stored at −70 °C until used for ChIP.

### RNA-seq library preparation and sequencing

RNA was isolated from merozoites and schizonts stored in TRI reagent using Zymo’s Direct-zol RNA microprep kit (Zymo #R2061) according to manufacturer’s instructions with DNase treatment. As an extra safe-guard step, isolated RNA was treated with DNase I (Invitrogen #AM1906) using the manufacturer’s protocol for rigorous DNase treatment. RNA quality was determined by Bioanalyzer, and its concentration determined by Qubit. RNA samples with an RNA integrity number > 7 were used for library preparation. High quality, strand-specific libraries for RNA-seq were prepared using the NEBNext Ultra II Directional RNA Library Prep Kit for Illumina (NEB #E7760S) with the NEBNext Poly(A) mRNA Magnetic Isolation Module (NEB #E7490) according to manufacturer’s instructions with the following modifications. Adaptor-ligated DNA was enriched by PCR with i7 index primers and i5 universal primer (NEBNext multiplex oligos for Illumina index primers set 1 and 2, NEB #E7335 and #E7500) using KAPA HiFi hot start ready mix (Roche # 07958919001) with the following program: initial denaturation at 98 °C for 45 s.; 9 cycles of (98 °C for 15 s., 55 °C for 30 s., 62 °C for 30 s.); and final extension at 62 °C for 5 min. Libraries were purified using 0.9 volumes sparQ beads (QuantaBio #95196-060) according to manufacturer’s instructions and sequenced with the Illumina NextSeq500 to generate 75 bp paired end reads.

### Chromatin immunoprecipitation (ChIP)

Crosslinked *P. falciparum* schizonts, merozoites, rings, and *P. berghei* merosomes were chromatin immunoprecipitated as described previously [64]. See the Additional file 3 for our detailed protocol. In short, crosslinked parasites were washed with 1 ml cold PBS containing protease inhibitors (1 tablet per 10 ml, cOmplete protease inhibitor cocktail, Sigma #4693159001) then incubated on ice with 1 ml nuclear extraction buffer [f.c.: 10 mM HEPES, 10 mM KCl (Fisher #BP366-500), 0.1 mM EDTA (Fisher #BP2482100), 0.1 mM EGTA (Boston BioProducts #BM1511), 1 mM DTT (Promega #P1171), 200 mM AEBSF (GoldBio #A-540–500), cOmplete protease inhibitor cocktail (1 tablet per 10 ml), PhosSTOP phosphatase inhibitor (Sigma #4906845001)]. After 35 min., NP-40/IGEPAL (Alfa Aesar #J61055AE) was added to a final concentration of 0.25% and the solution was homogenized by passing the mixture seven times through a 26 G needle. Extracted nuclei were pelleted by centrifugation at 2100 × *g* for 20 min. at 4 °C. Supernatant was removed and the pelleted nuclei were resuspended in 130 µl of cold shearing buffer [f.c.: 0.1% SDS (Fisher #BP1311-200), 1 mM EDTA, 10 mM Tris–HCl pH 7.5 (Fisher #BP1757-500), cOmplete protease inhibitor cocktail (1 tablet per 10 ml), PhosSTOP phosphatase inhibitor (1 tablet per 10 ml)] and transferred to a microTUBE (Covaris #NC9871616). Chromatin was fragmented with an M220 sonicator (Covaris) with the following settings: 5% duty cycle, 75 W peak incident power, 200 cycles per burst at 6 °C for 300 s. Fragmented chromatin was transferred to a pre-lubricated, low binding 1.5 ml tube. The microTUBE was washed with 70 µl of shearing buffer after which the solution was combined with the fragmented chromatin for a total volume of 200 µl. For each developmental stage (schizont, merozoite, ring, and hepatic merozoite) 30 µl of sheared chromatin was collected at this point for use as an input sample and stored at −70 °C until decrosslinking.

Chromatin was diluted 1:1 in ChIP dilution buffer [f.c.: 30 mM Tris–HCl pH 8.0 (Fisher #BP1758-500), 3 mM EDTA, 0.1% SDS, 300 mM NaCl (Fisher #S271-500), Triton X-100 (Fisher #BP151-100), supplemented with cOmplete protease inhibitor cocktail and PhosSTOP phosphatase inhibitor in nuclease-free water] then precleared by incubation with protein A agarose/salmon sperm DNA beads (50 µl beads per 1 ml sheared chromatin, Millipore Sigma #16-157) for 1 h at 4 °C with agitation to reduce non-specific background. Following the incubation, the beads were pelleted by centrifugation at 100 × *g* for 1 min. at RT. The supernatant containing the chromatin was moved to a new low-binding tube and incubated overnight at 4 °C with agitation with 2 µg of antibody (or no antibody for merozoite and schizont no antibody samples) against the protein of interest [H3K4me3 (Thermo #PA527029); H3K4me3 (Abcam #ab8580); H3K4me2 (Thermo #710796); H3K4me (Abcam #ab8895); H3K27me (Millipore Sigma #07448S); H3K18me (Abcam #ab177253); H3K27ac (Abclonal #A7253); H3K9ac (Abclonal #A7255)] (Additional file 2: Table S27). All antibodies were validated for ChIP by the manufacturer, except for anti-H3K18me, which was previously validated for ChIP-seq by Cheeseman et al*.* [32].

Protein A agarose/salmon sperm DNA beads (25 µl beads per 500 µl sonicated chromatin) were washed with 500 µl ChIP dilution buffer and blocked with 1 mg/ml BSA (MP Biomedicals #08810061) for 1 h at 4 °C with agitation to reduce non-specific binding. Agarose beads were washed 3 times with 500 µl ChIP dilution buffer, then added to the chromatin suspension and incubated for 1 h at 4 °C with agitation. Agarose beads were pelleted by centrifugation at 100 × *g* for 1 min. at RT, and the supernatant was discarded. Beads were washed twice at 4ºC for 15 min. with 1 ml high salt wash buffer [f.c.: 1% SDS, 1% Triton X-100, 2 mM EDTA, 20 mM Tris–HCl pH 8.0, 500 nM NaCl in nuclease-free water] at 4 °C with agitation. Next, the beads were washed twice with low salt wash buffer [f.c.: 1% SDS, 1% Triton X-100, 2 mM EDTA, 20 mM Tris–HCl pH 8.0, 150 mM NaCl, in nuclease-free water] for 15 min. at 4 °C with agitation, twice with lithium chloride wash buffer [f.c.: 0.25 M LiCl (Fisher #L121-100), 1% NP-40/IGEPAL, 1% sodium deoxycholate (Alfa Aesar #J6228814), 1 mM EDTA, 10 mM Tris–HCl pH 8.0 in nuclease-free water] for 15 min. at 4 °C with agitation, and finally, twice for 15 min. with TE buffer [f.c.: 10 mM Tris–HCl pH 8.0, 1 mM EDTA in nuclease-free water] at RT with agitation. Chromatin bound to agarose beads was eluted by incubating with 125 µl elution buffer [f.c.: 1% SDS, 0.1 M NaHCO_3_ (Millipore Sigma #SX03201) in nuclease-free water] for 15 min. at RT with agitation. The elution was repeated, and fractions combined for a total of 250 µl. Samples were reverse crosslinked by adding NaCl to a final concentration of 0.5 M and incubating overnight at 45 °C. RNase A (Invitrogen #12,091,021) was then added to a final concentration of 0.6 μg/ml and incubated at 37 °C for 30 min. Samples were then incubated with EDTA (f.c.: 1 mM), Tris–HCl pH 7.5 (f.c.:4 mM), and proteinase K (0.8 U, Zymo #D3001-2-5) for 2 h at 45 °C. DNA was isolated using 2 volumes sparQ beads (QuantaBio #95196-060) according to manufacturer’s instructions. ChIP DNA was stored at −20 °C until library preparation. Input samples were incubated with 0.6 μg RNase A per µl for 30 min. at 37 °C, then incubated with 0.8 U proteinase K overnight at 45 °C. DNA was isolated using 2 volumes sparQ beads according to manufacturer’s instructions and stored at ﻿−20 °C until library preparation.

### ChIP library preparation and sequencing

Libraries from ChIP samples were prepared using the NEBNext DNA Library Prep Master Mix Set for Illumina (NEB #E6040) or the NEBNext Ultra II DNA library prep kit for Illumina (NEB #E7645) according to manufacturer’s instructions with the following modification (see Additional file 2: Table S28 for which kit was used for which samples). Libraries were amplified using i7 index primers and i5 universal primer (NEBNext multiplex oligos for Illumina index primers set 1 and 2, NEB #E7335 and #E7500) with KAPA HiFi hot start ready mix (Roche #KK2601) with the following program: initial denaturation at 98 °C for 45 s.; 15 cycles of (98 °C for 15 s., 55 °C for 30 s., 62 °C for 30 s.); and final extension at 62 °C for 5 min. Libraries were purified using 0.9 volumes sparQ beads according to manufacturer’s instructions. Libraires were sequenced with the Illumina HiSeq 2500 or Illumina NextSeq500 to generate 50 bp single-end reads or 75 bp paired-end reads, respectively.

### Immunofluorescence assay (IFA)

IFA was performed as previously described [65]. All steps of the assay were performed at RT. In brief, thin blood smears were made on a microscopy slide from 1 µl of culture containing higher percentages of late-schizont or ring-stage parasites (determined by Giemsa staining) and allowed to air dry for 30 s. Smears were fixed by covering the slide with 4% paraformaldehyde in Milli-Q water for 30 min. The fixed cells were washed 3 times with 1 ml PBS then permeabilized by incubation with 0.1% Triton X (Fisher #BP151) in PBS for 30 min. Cells were again washed 3 times with 1 ml PBS. Slides were treated with blocking buffer (2% BSA, 0.05% Tween-20, 100 mM Glycine, 3 mM EDTA and 150 mM NaCl in PBS) for 1 h by covering the top of the slide. The slides were coated with primary antibodies against either H3K18me (f.c.: 1:5000 dilution, Abcam #ab177253) or H3K27me (1:50 dilution, Millipore Sigma #07448S) in 1 ml blocking buffer and were incubated for 1 h. Slides were washed with 1 ml PBS for a total of 3 times. Goat anti-rabbit IgG conjugated to AlexaFluor Plus 647 (5 µg/µl, Invitrogen #A32733) in blocking buffer was incubated on the slide for 1 h in the dark. Slides were again washed thrice and allowed to air dry in the dark. Dry slides were mounted with 10 µl ProLong Glass mounting medium containing NucBlue stain (Thermo #P36985) and sealed with a cover slip. Samples were imaged using a Zeiss Axio Imager Z1 with Zen Blue software.

### Western blotting

*P. falciparum* parasites (25 ml culture) were isolated by saponin treatment as described above. The parasite pellet was resuspended in 50 µL cytoplasmic lysis buffer (10 mM (f/c) Tris–HCl pH 7.5, 150 mM (f/c) NaCl, 1 mM (f/c) EDTA, 1 mM (f/c) EGTA, 2 mM (f/c) AEBSF (GoldBio #A-540-500), 0.65% (f/c) IGEPAL CA-630 (Thermo #J61055-AE), 1 × (f/c) protease inhibitor cocktail (Sigma #4693159001)) and passed through a 26 G needle 15 times to break the parasite’s cellular membrane. After centrifugation at 14,500 × g for 15 min at 4˚C the supernatant containing cytoplasmic protein was removed and the pellet of nuclei was washed once with 50 µL cytoplasmic lysis. After centrifuging at 14,500 × g for 15 min at 4 °C, the supernatant was removed and the nuclei were resuspended in 70 µL shearing buffer (10 mM (f/c) Tris–HCl pH 7.5, 1 mM (f/c) EDTA, 0.1% (f/c) SDS (Fisher # BP1311-200), 1 × (f/c) protease inhibitor cocktail, 1 × (f/c) PhosSTOP (Sigma #4906845001)), passed through a 26 G needle seven times to disrupt the nuclear membrane and sonicated using a Covaris M220 sonicator and Covaris tubes (#520045)) to shear the chromatin (5% duty cycle, 75 W peak incident power, 200 cycles per burst, 6 °C, 300 s). After transferring the sample, the Covaris tube was washed with 30 µL shearing buffer to collect any remaining protein. After centrifugation at 14,500 × g for 10 min at 4˚C the supernatant containing the nuclear proteins was collected and stored at −70 °C.

Expi293F (human) cells were collected by centrifugation of the culture at 200 × g for 5 min at RT. The cells were lysed using a protein lysis buffer (100 mM (f/c) Tris–HCl pH 7.5, 150 mM (f/c) NaCl, 0.5 mM (f/c) EDTA, 0.5% (f/c) Triton X-100, 1 × (f/c) protease inhibitor cocktail). *E. coli* lysate was purchased from Molecular Cloning Lab (#ECCL-100).

For all samples, protein quantification was performed using a Bradford coomassie assay (Thermo #23236). The following amounts of protein were loaded on a gel: 12.5 µg, 5 µg, and 5 µg for ring, trophozoite, and schizont stage parasites, respectively, and 2.5 µg for Expi293F and *E. coli* lysates. Samples were mixed 1:1 (v/v) with 2 × Laemmli sample buffer (Bio-Rad #1610737) that was supplemented with 5% (f/c) β-Mercaptoethanol (MP Biomedicals #219483425) and heated at 85 °C for two minutes prior to loading on a 4–12% Bolt Bis–Tris protein gel (Thermo # NW04120BOX). Two identical gels were run at 200 V in MOPS SDS Running Buffer (Thermo #NP0001) and stopped when the 10 kDa marker from the PageRuler Plus Prestained Protein Ladder (Thermo #26,619) was at the bottom of the gel. After rinsing the gel with deionized water, proteins were transferred onto a 0.45 µm PVDF transfer membrane (Thermo #88518) that was activated by incubation in methanol (Fisher Scientific #A452-4) for 30 s. Proteins were transferred to the membrane using a Mini Blot Module (Thermo #B1000) and NuPage Transfer Buffer (Thermo #NP00061) with 20% methanol for 60 min at 25 V. After washing the membrane twice with deionized water, non-specific interactions were blocked using 5% 2 × NAP (Non Animal Protein)-Blocker (G-Biosciences #786190P) in PBST (0.05% Tween-20 (Fisher Scientific #BP337-100)) for 1 h at RT, with gentle agitation. Primary antibodies anti-H3K18me (ABCAM # AB177253) and anti-H3K27me (Sigma # 07-448-S) were diluted 1,000 × in blocking buffer. After overnight incubation (different membrane for each of these two histone markers) at 4 ﻿°C with the primary antibody, the membrane was washed (five times 5 min) with 0.05% PBST. Subsequently, the membrane was incubated for 1 h at RT with secondary goat anti-rabbit StarBright Blue 520 (Bio-Rad #12005870) or goat anti-rabbit HRP (Thermo # A16116) antibody (2,500 × dilution in blocking buffer), respectively. The membranes were washed (five times 5 min) with 0.05% PBST and then with deionized water. The membrane probed for H3K27me was incubated with 10 ml 1-Step Ultra TMB Blotting Solution (Thermo #37574) for 10 min at RT and washed twice with deionized water. Both membranes were imaged on a Bio-Rad ChemiDoc MP imaging system. For detection of H3, the membrane probed for H3K18me was blocked again and incubated as described above. Anti-H3 (1,000 × dilution; BioLegend #819411) and goat anti-mouse StarBright Blue 700 (2,500 × dilution, Bio-Rad #12004159) were used as primary and secondary antibodies, respectively.

### Bioinformatics

#### Read mapping

Paired-end data generated for histone PTM ChIP-seq was treated as single-end data so that all ChIP-seq data could be processed in the same way, and only read one was processed for analysis. RNA-seq data was processed as paired-end. Following quality assessment of the reads using FastQC (v0.11.8) (reads with a quality score ≥ 30 were retained), adaptor sequences were trimmed using Trim-galore (v0.5.0) (https://www.bioinformatics.babraham.ac.uk/projects/fastqc; https://www.bioinformatics.babraham.ac.uk/projects/trim_galore). ChIP-seq data were mapped to the *P. falciparum* 3D7 genome v3.0, release 44 or the *P. berghei* ANKA genome v3.0, release 49 (PlasmoDB, [66]) with Bowtie2 (v2.3.5) [67] while RNA-seq data was mapped using hisat2 (v2.1.0) with a maximum intron length of 2500 bp [68]. For both ChIP-seq and RNA-seq data, reads were filtered with SAMtools (v1.9) to remove sequences that aligned to the genome more than once (option -q 10) and duplicates were removed using MarkDuplicates from Picard (v2.18.29-SNAPSHOT) ([69]; http://broadinstitute.github.io/picard). Bam files were generated using bamCoverage from the deepTools suite (v3.2.0) and normalized using the RPGC option. Using bamCompare from the deepTools suite, log_2_(ChIP/input) bigwig files with reads extended to 150 bp were generated from bam files. These files were viewed using IGV (v2.5.0) [70, 71]. Summary statistics for ChIP-seq mapping are available in Additional file 2: Table S28. For RNA-seq visualization, bam files generated with bamCoverage (normalized with the RPGC option) were converted to bigwig files using bamCompare and viewed with IGV.

For analysis of RNA-seq data from Wichers et al*.* trimmed fastQ files (fq.gz files) were downloaded from the ArrayExpress database (http://www.ebi.ac.uk/arrayexpress, accession number E-MTAB-7731, [33]). Reads were mapped to the *P. falciparum* genome and processed as described above. Summary statistics for RNA-seq mapping of our samples and the samples from Wichers et al*.* are available in Additional file 2: Table S29.

#### Differential gene expression analysis

Raw sequencing counts per gene for each replicate and stage (Additional file 2: Table S30) were calculated using htseq-count from the Python package HTseq (v2.0.2) [72]. Bam files generated after duplicate removal as described above were used as input. Reads were assigned using genes as the feature type based on the PlasmoDB-44 *P. falciparum* GFF file ([72], PlasmoDB). We excluded non-coding RNA (including rRNA) from this analysis.

Differential gene expression analysis was performed using the R package DESeq2 (v1.36.0) with the raw sequencing counts generated with htseq-count [73]. For the identification of differentially expressed genes between the early schizont and merozoite samples generated by us, early schizont samples (40 hpi, n = 3) were compared to merozoites (n = 3). Genes with a log_2_(fold change) less than -1.5 or greater than 1.5 and an adjusted p-value < 0.1 (adjusted for multiple testing by the Benjamini–Hochberg procedure [73]) were considered differentially expressed and were shown in the heatmap in Fig. 1A. Results from this analysis are provided in Additional file 2: Tables S1 and S2.

To identify genes differentially expressed between all early schizonts (40 hpi, n = 6, samples from Wichers et al*.* and us) and merozoites (n = 6, samples from Wichers et al*.* and us), we performed another differential gene expression analysis between early schizonts (n = 6) and merozoites (n = 6). Genes with a log_2_(fold change) less than -1.5 or greater than 1.5 and an adjusted p-value < 0.1 (adjusted for multiple testing by the Benjamini–Hochberg procedure) were considered differentially expressed and used for further analysis (Additional file 2: Table S3, S4). To identify differentially expressed genes between merozoites (n = 6) and rings (8 hpi, n = 3), we performed a separate DESeq2 analysis comparing merozoites to rings (Additional file 2: Tables S3, S6).

Heatmaps depicting differentially expressed genes or specific groups of genes (i.e., H3K4me3 depletion genes, H3K4me2 enrichment genes, etc.) were generated using the R package pheatmap (v1.0.12) (https://CRAN.R-project.org/package=pheatmap). DESeq2 results were log transformed prior to graphing. Genes were clustered using the k-means option in pheatmap (v1.0.12). The number of clusters was determined based on the within cluster sum of squares (elbow method) using the R packages factoextra (v1.0.7) (https://rpkgs.datanovia.com/factoextra/index.html) and NbClust (v3.0.1) [74]. Principal component analysis was performed with the plotPCA function from DESeq2 using log transformed DESeq2 data and graphed with GraphPad Prism (v9.4.1). Average RNA-seq expression for specific groups was calculated by taking the average of the DESeq2 normalized expression values of all genes within a group for each sample. Boxplots of average expression were generated with GraphPad Prism 9.

#### ChIP-seq data analysis

Distributions of histone PTMs across genes or regions of interest were calculated using computeMatrix from the deepTools suite (v3.2.0) with the scale-regions option and log_2_(ChIP/Input) bigwig files as input. Profiles and corresponding heatmaps were generated using plotHeatmap from deepTools. For the graphs shown in Figs. 2D, 3D, F4A, and Additional file 1: Fig. S4 enrichment values across the genes or regions were calculated with computeMatrix and plotProfile from deepTools and graphs were generated using GraphPad Prism 9.

For analysis of the relationship between gene expression and H3K4me3, H3K9ac, and H3K27ac in merozoites, the average log_10_FPKM for each gene from merozoite replicate 1 and 4 (samples with the highest gene expression in merozoites) was compared to H3K4me3, H3K9ac, or H3K27ac enrichment in the promoter region. FPKM was calculated for each sample using count2FPKM from R package RNAAgeCalc (v1.2.0) and the average was taken [75]. To calculate the enrichment of H3K4me3, H3K9ac, and H3K27ac in the promoter region, a file containing coordinates for the 1000 bp upstream of all gene start sites was generated and htseq-count from the Python package HTseq (v2.0.2) [72] along with the ChIP bam files described above were used to determine the read coverage over this region. Read coverage in input files was also determined. H3K4me3, H3K9ac, and H3K27ac coverage is reported as log_2_(ChIP/input). Linear regression analysis was performed with GraphPad Prism 9.

Pearson correlations between all libraries generated for ChIP-seq were calculated with multiBigWigSummary and graphed with GraphPad Prism 9.

#### ChromHMM analysis

A detailed description of ChromHMM and its usage is available in Ernst et al*.* [35]. In brief, we generated the chromatin states model using H3K4me3, H3K27ac, H3K9ac, and input bam files from early schizonts (40 hpi), merozoites, and early rings (4 hpi) by concatenating cell types to generate one model with cell-type specific annotations. Input data was used to adjust the binarization threshold locally. The ChromHMM model was generated based on 5 unique states and enrichment analysis around specific features was performed with OverlapEnrichment. Raw model values can be found in Additional file 2: Tables S8 and S9. Coordinate files used for enrichment analysis of promoter regions (1000 bp upstream of coding sequence start coordinate from PlasmoDB-49 GFF file) were generated for all genes in the genome, schizont-expressed genes (Additional file 2: Table S13), ring-expressed genes (Additional file 2: Table S14), and genes with H3K4me3 depletion in the upstream intergenic region (Additional file 2: Table S12). Schizont and ring expressed genes were obtained from Bunnik et al*.*, and only genes from steady-state mRNA expression cluster S (for schizont-expressed genes) or R (for ring-expressed genes) were included in this analysis [55]. Heatmaps depicting the ChromHMM model and the enrichment around promoter regions were generated in R using ggplot2 (v3.3.6) (https://CRAN.R-project.org/package=ggplot2).

To identify regions of the genome with the 1–5–1 ChromHMM pattern, we generated segmentation files for the early schizont, merozoite, and early ring samples with the MakeSegmentation function. Using this file and a custom Perl script (available in the Additional file 3) we identified all instances of the 1–5–1 ChromHMM state pattern in the genome. We manually determined which instances fell in intergenic regions (n = 66). To associate the 1–5–1 regions with genes, we viewed the regions in IGV and assigned genes to the region that immediately preceded the gene start site. These annotations are provided in Additional file 2: Table S11.

#### Gene ontology analysis

Gene ontology (GO) analysis was performed by Fisher’s exact test with correction by false discovery rate calculation using The Gene Ontology Resource (http://geneontology.org). Terms with an FDR p-value < 0.05 were considered significantly enriched or depleted, but only terms with a fold enrichment are reported in Additional file 2: Tables S5 and S7. Terms relating to biological process, molecular function, and cellular component were assessed.

#### Plasmodium berghei RNA-seq analysis

For analysis of the relationship between gene expression and enrichment of H3K4me3 and H3K9ac, raw sequencing counts per gene from *P. berghei* hepatic late schizonts (60 hpi), hepatic merozoites (69 hpi), and erythrocytic early rings (4 hpi) as reported by Caldelari et al*.* [49] are shown in Additional file 2: Table S31. Raw sequencing counts for hepatic merozoites (69 hpi) from Caldelari et al*.* were converted to FPKM values using count2FPKM from R package RNAAgeCalc (v1.2.0) [49, 75]. The distribution of H3K4me3 and H3K9ac over the coding sequencing was calculated with computeMatrix from deepTools. The distribution profile and heatmap of genes ordered by FPKM value were generated with plotHeatmap from deepTools.

For analysis of the relationship between gene expression, H3K4me3, and H3K9ac in *P. berghei* merozoites, log_10_FPKM for each gene in hepatic merozoites (69 hpi) was compared to H3K9ac enrichment in the promoter region. To calculate the enrichment of H3K9ac and H3K4me3 in the promoter region, a file containing coordinates for the 1000 bp upstream of all gene start sites was generated and htseq-count from the Python package HTseq (v2.0.2) [72] along with the ChIP bam files described above were used to determine the read coverage over this region. Read coverage was also determined for the input file. H3K9ac and H3K4me3 coverage is reported as log_2_(ChIP/input). Linear regression analysis was performed with GraphPad Prism 9.

To assess changes in the expression of genes with H3K4me3 depletion in the promoter region, we performed differential gene expression analysis with DESeq2 using the raw sequencing counts as reported by Caldelari et al*.* [75]. For this analysis, hepatic early schizonts (48 hpi, n = 2) were compared to hepatic merozoites (69 hpi, n = 2). The DESeq2 results for genes with H3K4me3 depletion are shown in Additional file 2: Table S20. DESeq2 values for genes in 48 hpi hepatic schizonts (48 hpi, n = 2), 54 hpi hepatic schizonts (54 hpi, n = 2), 60 hpi hepatic schizonts (60 hpi, n = 2), hepatic merozoites/DCs (69 hpi, n = 2), and erythrocytic early rings (4 hpi, n = 2) are also shown in this table. Average RNA-seq expression was calculated as described above. For comparison, the average expression of 147 random *P. berghei* genes was also calculated (Additional file 2: Tables S32, S33).

#### Plasmodium berghei H3K4me3 depletion analysis

We used a custom Perl script (available as in the Additional file 3) to identify regions of the genome with at least a threefold depletion of H3K4me3 compared to H3K9ac over a stretch of at least 250 bp. Genome coverage files generated using bamCoverage from deepTools for H3K4me3 and H3K9ac (not normalized to input values) were used as input. To associate these regions with genes, we viewed the regions in IGV and annotated each region with the gene(s) that had start sites immediately following the region. These annotations are provided in Additional file 2: Table S18.

#### Motif analysis

Motif analysis was performed using MEME [76]. Motif searches were restricted to the region of H3K4me3 depletion. For the 1–5–1 panel genes, this corresponded to the limits of ChromHMM state 5. Default MEME settings were used and motif searches were restricted to find motifs between 6 and 8 bp long as this is the average length of ApiAP2 transcription factor binding motifs. E-values for all motifs are reported.

Enrichment of the AP2-EXP core motif (CATGC) in the region of H3K4me3 depletion or randomly selected intergenic regions was performed using Simple Enrichment Analysis (SEA) with default settings [46]. Shuffled primary sequences with conserved 3-mer frequencies were used as control sequences. Randomly selected intergenic regions were comprised of a sequence of 201–1401 bp in length directly upstream of the ATG translation start site of 74 randomly selected genes (first 74 genes from Additional file 2: Table S25). Sequence length was selected such that the distribution and average length of the randomly selected sequences was the same as the regions with H3K4me3 depletion.

#### H3K4me2 enrichment analysis

Read counts per gene were calculated for H3K4me2 and input bam files using htseq-count. H3K4me2 read counts per gene were normalized to read counts per gene from input files followed by calculation of the log_2_(fold-change) difference between early schizonts (40 hpi) and merozoites or merozoites and early rings (8 hpi). Genes with a positive log_2_(fold-change) for both merozoites vs. early schizonts and merozoites vs. early rings (corresponding with an enrichment of H3K4me2 in merozoites compared to both early schizonts and early rings) were retained for analysis. Distribution of H3K4me2 as log_2_(ChIP/input) in these genes for early schizonts (40 hpi), merozoites, and early rings (4 hpi) was graphed over the CDS with computeMatrix and plotHeatmap.

Enrichment of gene families was determined by Chi-squared test. The total number of genes in each family (*rifin*, *stevor, var,* and exported proteins) was determined by filtering the PlasmoDB-49 GFF file for entries annotated as “genes”, and then extracting all genes belonging to each family, excluding pseudogenes. All other genes (ones not contained within the *rifin, stevor, var,* or exported proteins) were considered “other.” The total number of non-coding RNAs was determined by filtering the PlasmoDB-49 GFF file for entries annotated as “RNA”.

#### H3K18me and H3K27me analysis

Read counts per gene were calculated for H3K18me, H3K27me, and input files using htseq-count. H3K18me and H3K27me read counts per gene were normalized to read counts per gene calculated from input files. To determine the enrichment of each mark across individual chromosomes, we first divided the read counts for each gene by the gene length (difference between gene end and start as reported in PlasmoDB-49 GFF file) to generate normalized counts per kilobase for each gene. We then determined the proportional distance from the centromere to the start of each gene. To visualize distribution across the chromosome, we graphed the counts per kilobase for each gene against the proportional distance of the gene from the centromere using GraphPad Prism 9. Non-linear regression analysis was performed using GraphPad Prism 9.

## Supplementary Information

**Additional file 1.** Additional figures.

**Additional file 2. **Additional tables.

**Additional file 3.** Additional methods.

## Data Availability

All sequencing files are available from the NCBI Gene Expression Omnibus under accession no. GSE215429.
