# Supplementary material for: Histone modification analysis reveals common regulators of gene expression in liver and blood stage merozoites of Plasmodium parasites
Source: Epigenetics Chromatin. 2023 Jun 15;16:25. doi: 10.1186/s13072-023-00500-y (PMC10268464; doi:10.1186/s13072-023-00500-y)
Supplement: Supplementary file 1 — Additional file 1. Additional figures. [file 13072_2023_500_MOESM1_ESM.pdf]

Supplementary Figures to:

Histone modification analysis reveals common regulators of gene expression in liver and blood stage merozoites of *Plasmodium* parasites

Ashley B. Reers[1], Rodriel Bautista[1], James McLellan[2], Beatriz Morales[2], Rolando Garza[1], Sebastiaan Bol[1], Kirsten K. Hanson[2], Evelien M. Bunnik[1,3]

[1]Department of Microbiology, Immunology, and Molecular Genetics, Long School of Medicine, University of Texas Health Science Center, San Antonio, TX, USA

[2]Department of Molecular Microbiology and Immunology and South Texas Center for Emerging Infectious Diseases, University of Texas San Antonio, San Antonio, TX, USA

[3]Corresponding author

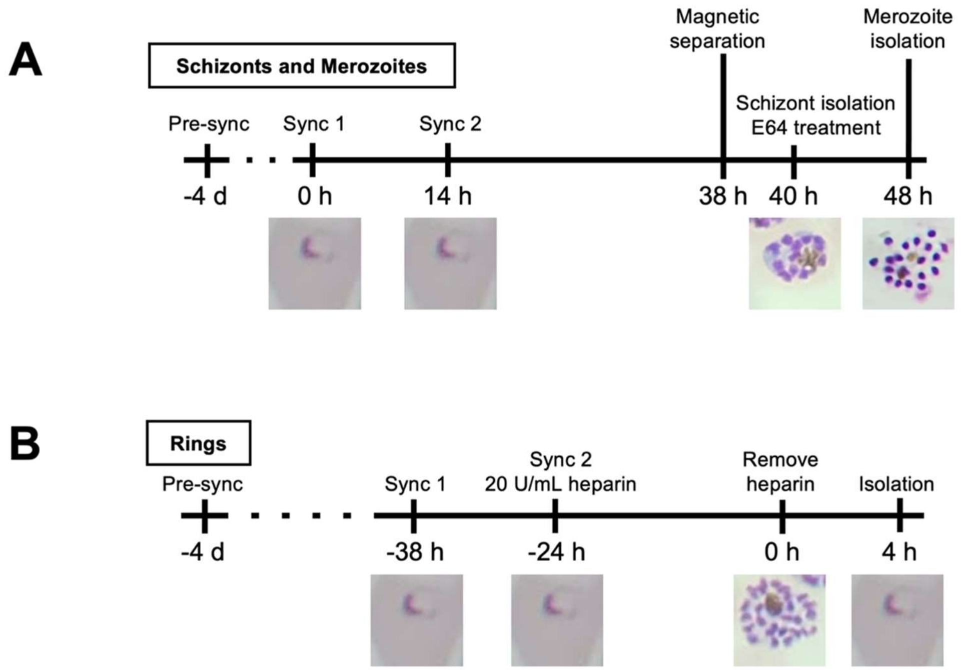

Figure S1. Isolation of early schizonts, merozoites, and early rings for RNA-seq and ChIP-seq. A) Isolation strategy for early schizonts (40 hpi) and merozoites for RNA-seq and ChIP-seq. **B)** Schematic of isolation protocol for early rings (4 hpi) for ChIP-seq. Representative images of Giemsa-stained parasites at each stage are shown.

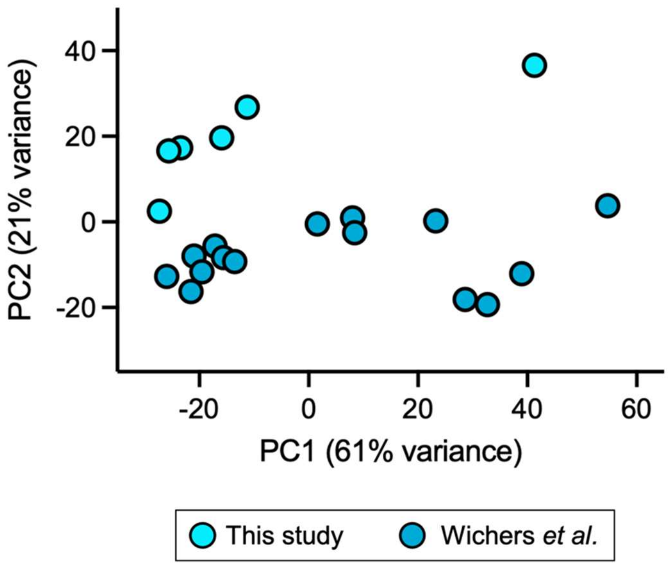

Figure S2. Principal component analysis of all RNA-seq samples. Samples are colored based on whether they were generated by Wichers *et al.* or as a part of this study. Amount of variance between samples accounted for by each component is shown on the x- and y- axes.

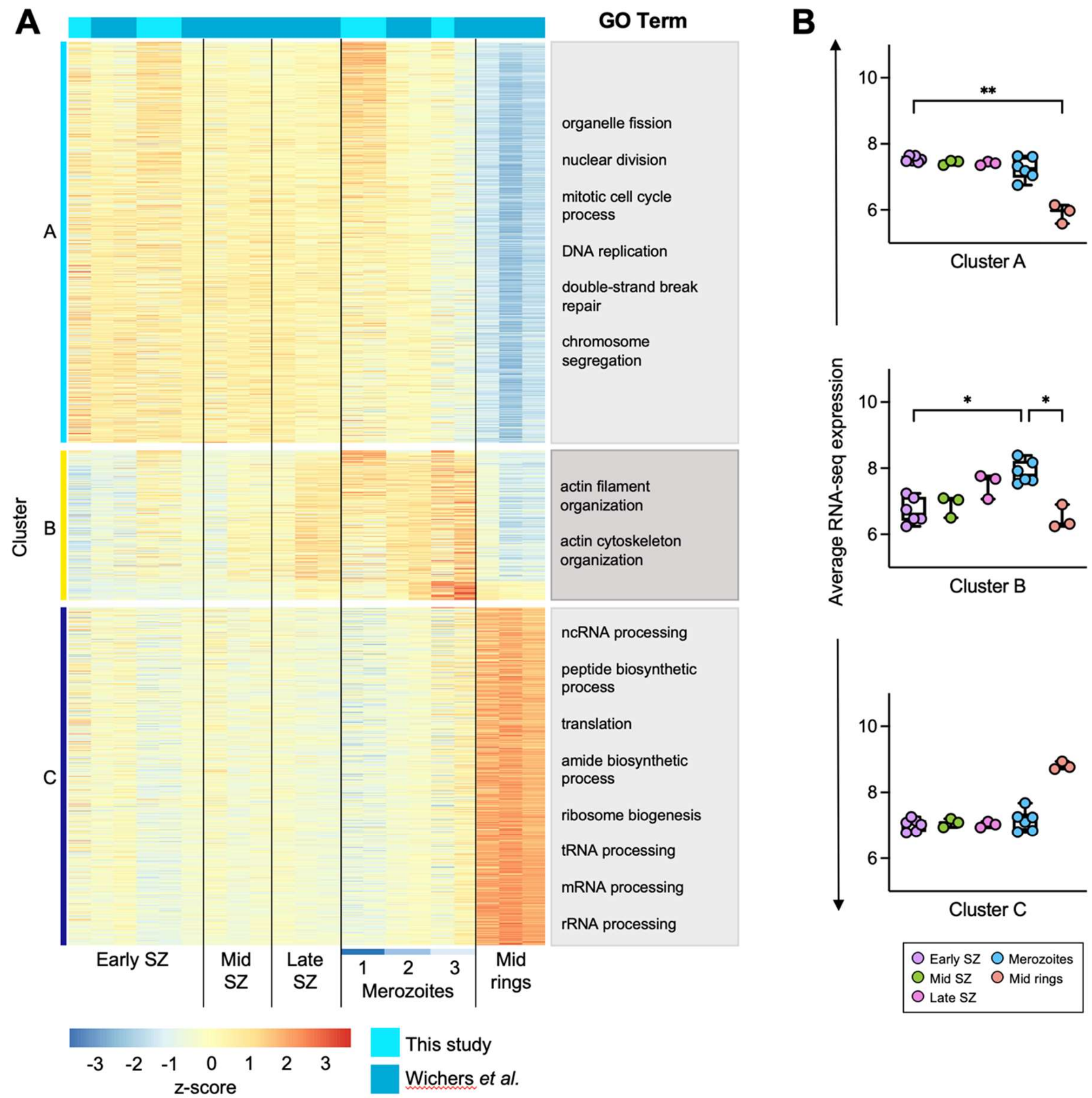

Figure S3. Genes differentially expressed between merozoites and rings. A) Heatmap depicting differentially expressed genes ($\log_2$(fold change) > 1.5 or < -1.5 and adjusted p-value < 0.1) between merozoites (n=6) and mid rings (8 hpi, n=3). Genes are divided into 3 clusters based on expression pattern by k-means clustering as indicated on the left of the heatmap. Merozoite subpopulations are indicated by the labels on the bottom of the heatmap. Enriched gene ontology (GO) terms in each cluster are displayed to the right of the heatmap. **B)** Boxplots of average gene expression of each cluster. Differences in gene expression were tested using a one-way Kruskal-Wallis test. P-values indicated in the graph are from Dunn's post hoc tests. *, p < 0.05; **, p < 0.01.

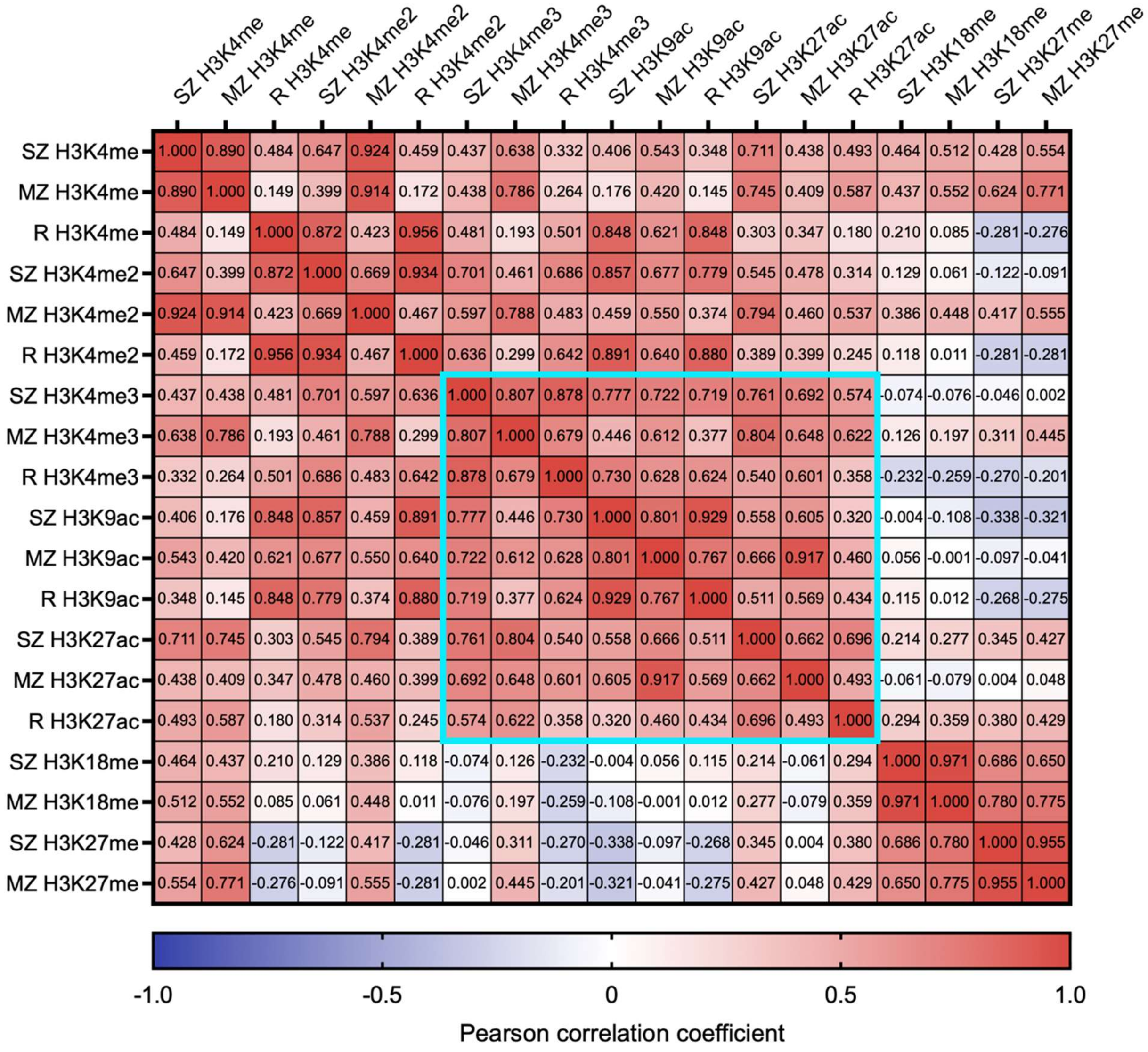

Figure S4. Correlations between all histone PTMs in early schizonts, merozoites, and early rings. Correlation between each mark in early schizonts (SZ), merozoites (M), or early rings (R) indicated on the x-axis and each mark in each stage indicated on the y-axis. Pearson correlation coefficients for each comparison are reported in each box. The blue box highlights correlations between H3K4me3, H3K9ac, and H3K27ac.

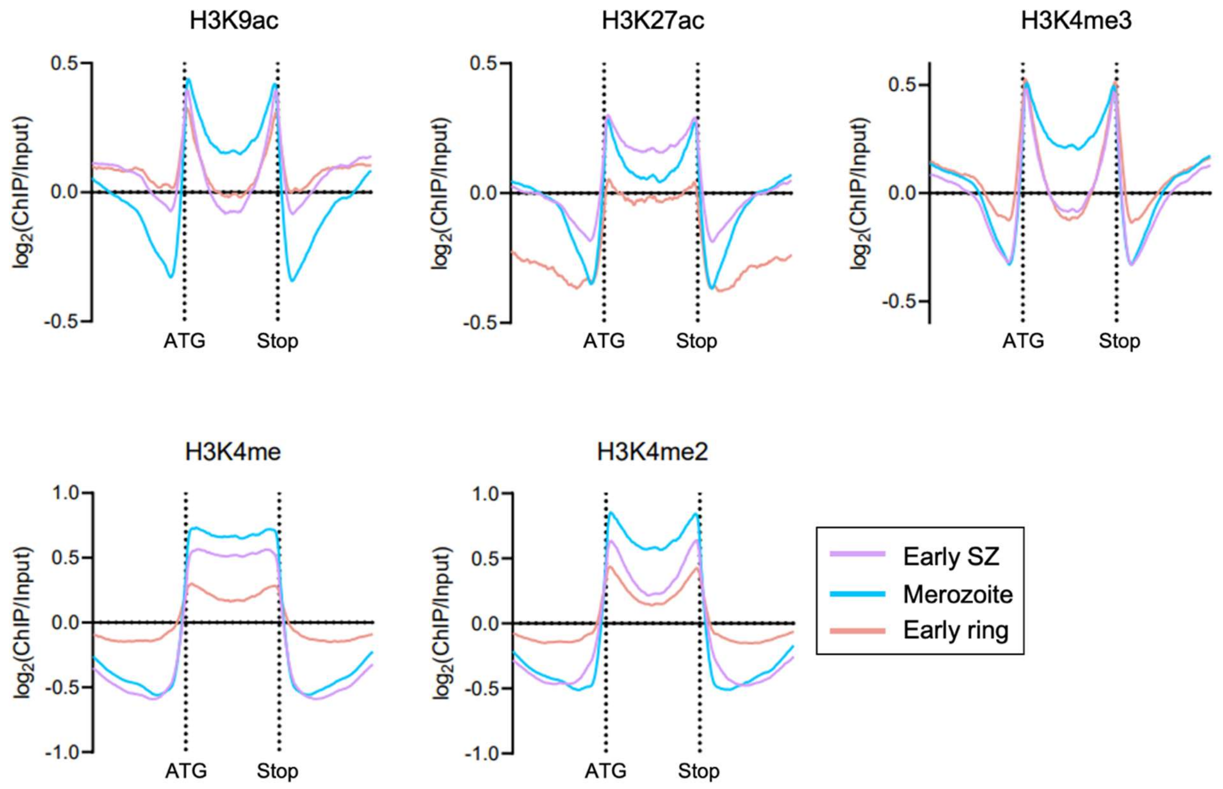

Figure S5. Global distribution of histone post-translational modifications around *P. falciparum* genes. Log_2(ChIP/input) enrichment of H3K9ac, H3K27ac, H3K4me3, H3K4me, and H3K4me2 across all genes (ATG to stop codon, n=5,602) for early schizont (40 hpi), merozoite, and early ring (4 hpi) genomes. A region 1.0 kb upstream of the ATG and downstream of the gene stop codon is also shown.

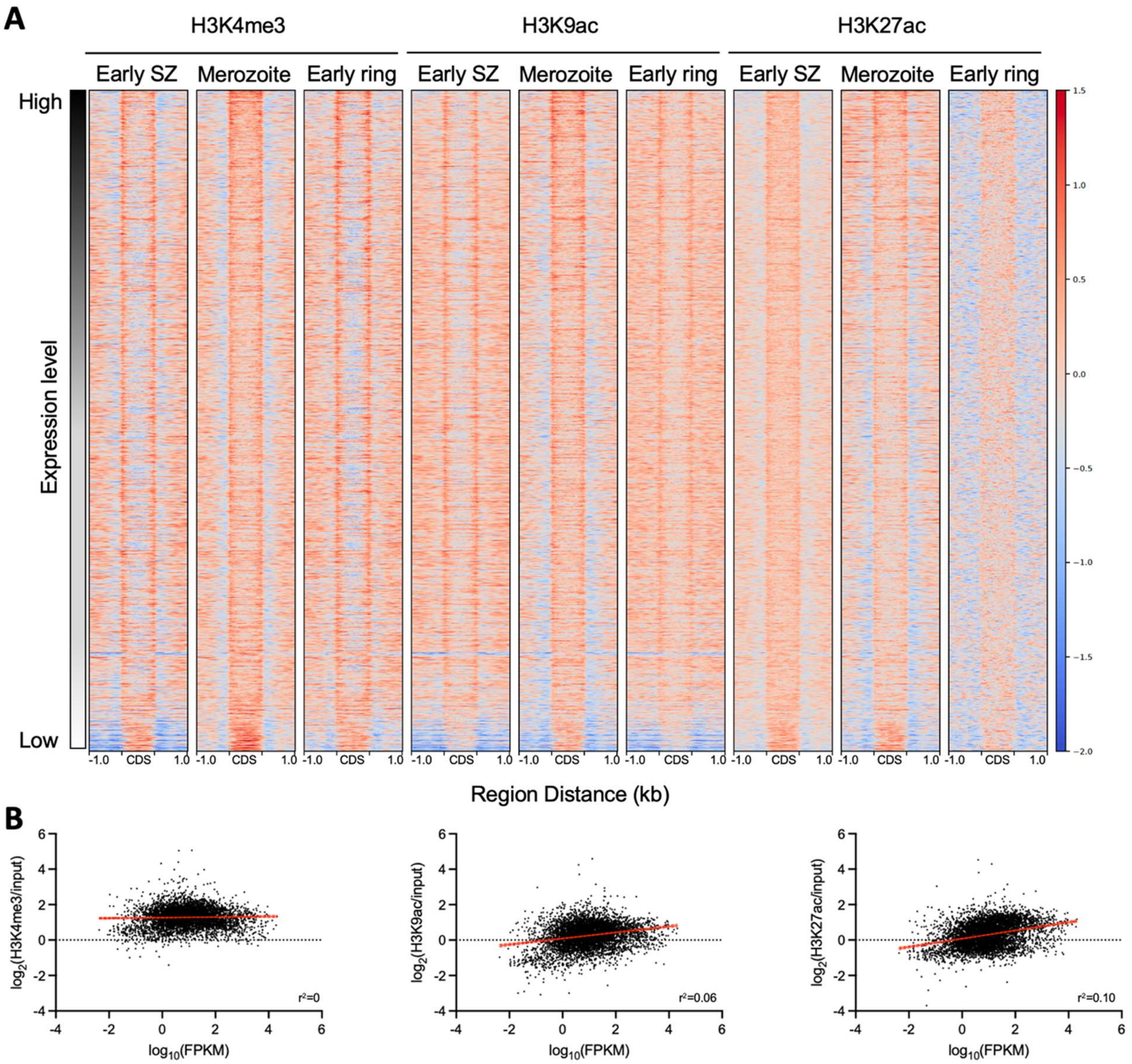

Figure S6. Relationship between H3K4me3, H3K9ac, and H3K27ac and gene expression. A) Heatmaps depicting H3K4me3, H3K9ac, or H3K27ac enrichment in the coding sequence (CDS). 1.0 kb up- and downstream of the CDS for all genes (n=5,602) in the genome is also depicted. Each row represents a gene. Columns are sorted based on gene expression in merozoite subpopulation 3 (**Figure 1C**). **B)** Gene expression in merozoite subpopulation 3 (log_{10}FPKM) plotted against H3K4me3, H3K9ac, or H3K27ac enrichment (log_2(ChIP/input)) in the promoter region (1,000 bp upstream of the gene ATG). Line of best fit as determined by linear regression analysis depicted by red line. The 95% confidence interval of the line of best fit is indicated by dashed red lines.

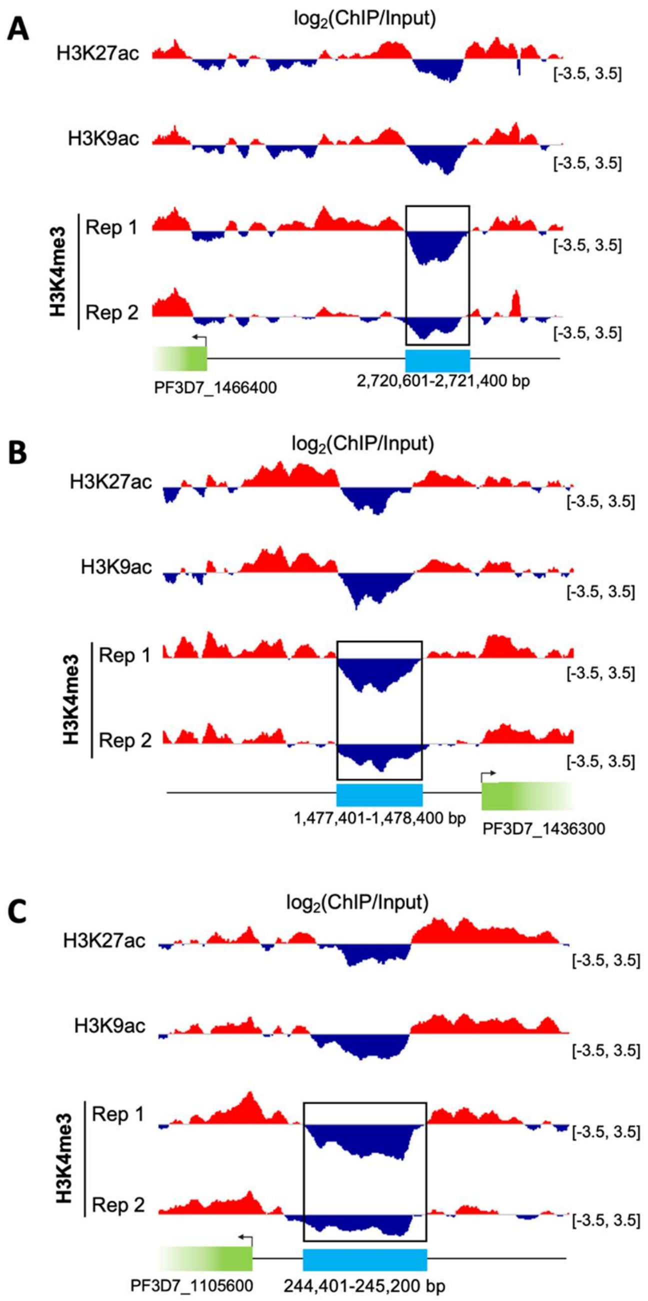

Figure S7. Merozoite H3K4me3 replicates. A-C) H3K27ac, H3K9ac, and H3K4me3 replicate ChIP tracks depicting H3K4me3 depletion in the intergenic region upstream of **B)** AP2-EXP (PF3D7_1466400), **B)** PTEX150 (PF3D7_1436300), and **C)** PTEX88 (PF3D7_1105600) in merozoites. Black rectangles indicate regions of H3K4me3 depletion. In the bottom track, the gene body is indicated by a green box, while the region of H3K4me3 depletion and corresponding coordinates are indicated by a blue box. Data range for ChIP-tracks is indicated in brackets to the right.

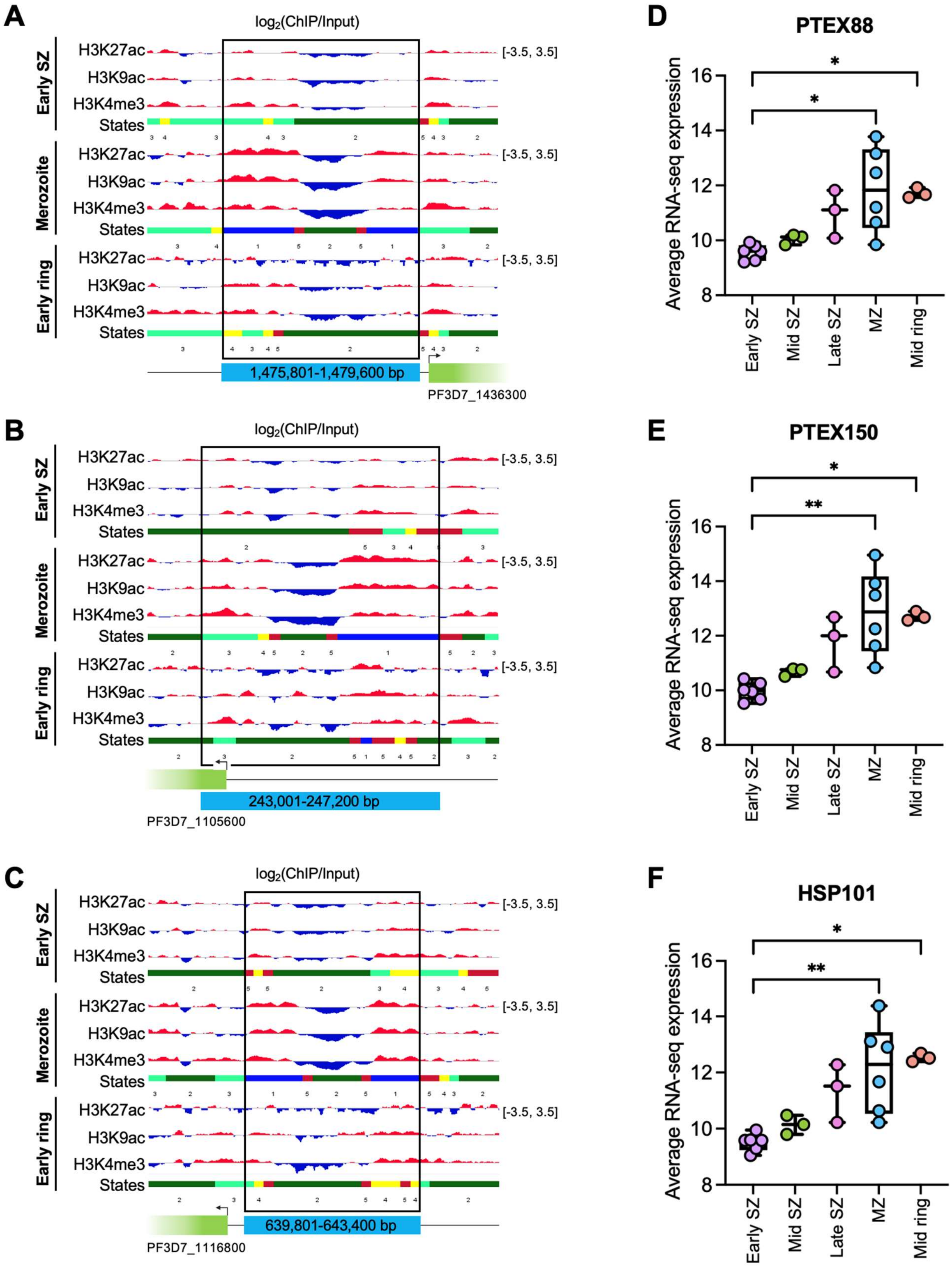
A
log2(ChIP/Input)
Early SZ
H3K27ac
H3K9ac
H3K4me3
States
Merozoite
H3K27ac
H3K9ac
H3K4me3
States
Early ring
H3K27ac
H3K9ac
H3K4me3
States
[-3.5, 3.5]
1,475,801-1,479,600 bp
PF3D7_1436300
B
log2(ChIP/Input)
Early SZ
Merozoite
Early ring
[-3.5, 3.5]
243,001-247,200 bp
PF3D7_1105600
C
log2(ChIP/Input)
Early SZ
Merozoite
Early ring
[-3.5, 3.5]
639,801-643,400 bp
PF3D7_1116800
D
PTEX88
Average RNA-seq expression
8
10
12
14
16
Early SZ
Mid SZ
Late SZ
MZ
Mid ring
*
*
E
PTEX150
Average RNA-seq expression
*
**
Early SZ
Mid SZ
Late SZ
MZ
Mid ring
F
HSP101
Average RNA-seq expression
*
**
Early SZ
Mid SZ
Late SZ
MZ
Mid ring

Figure S8. H3K4me3 depletion upstream of PTEX genes. A-C) H3K27ac, H3K9ac, and H3K4me3 log_2(ChIP/input) tracks in early schizonts (SZ), merozoites, and early rings for **A)** PTEX150 (PF3D7_1436300), **B)** PTEX88 (PF3D7_1105600), and **C)** HSP101 (PF3D7_1116800). Genes are indicated by green boxes while blue boxes represent the region of H3K4me3 depletion flanked by H3K9ac and H3K27ac enrichment upstream of genes. **D-F)** DESeq2 normalized gene expression in early schizonts (early SZ, 40 hpi), mid schizonts (mid SZ, 44 hpi), late schizonts (late SZ, 48 hpi), merozoites (MZ), and mid rings (mid ring, 8 hpi) of **D)** PTEX150, **E)** PTEX88, and **F)** HSP101. Differences in gene expression were tested for significance using a one-way Kruskal-Wallis test. P-values indicated in the graphs are from Dunn's post hoc tests. *, $p<0.05$; **, $p<0.01$.

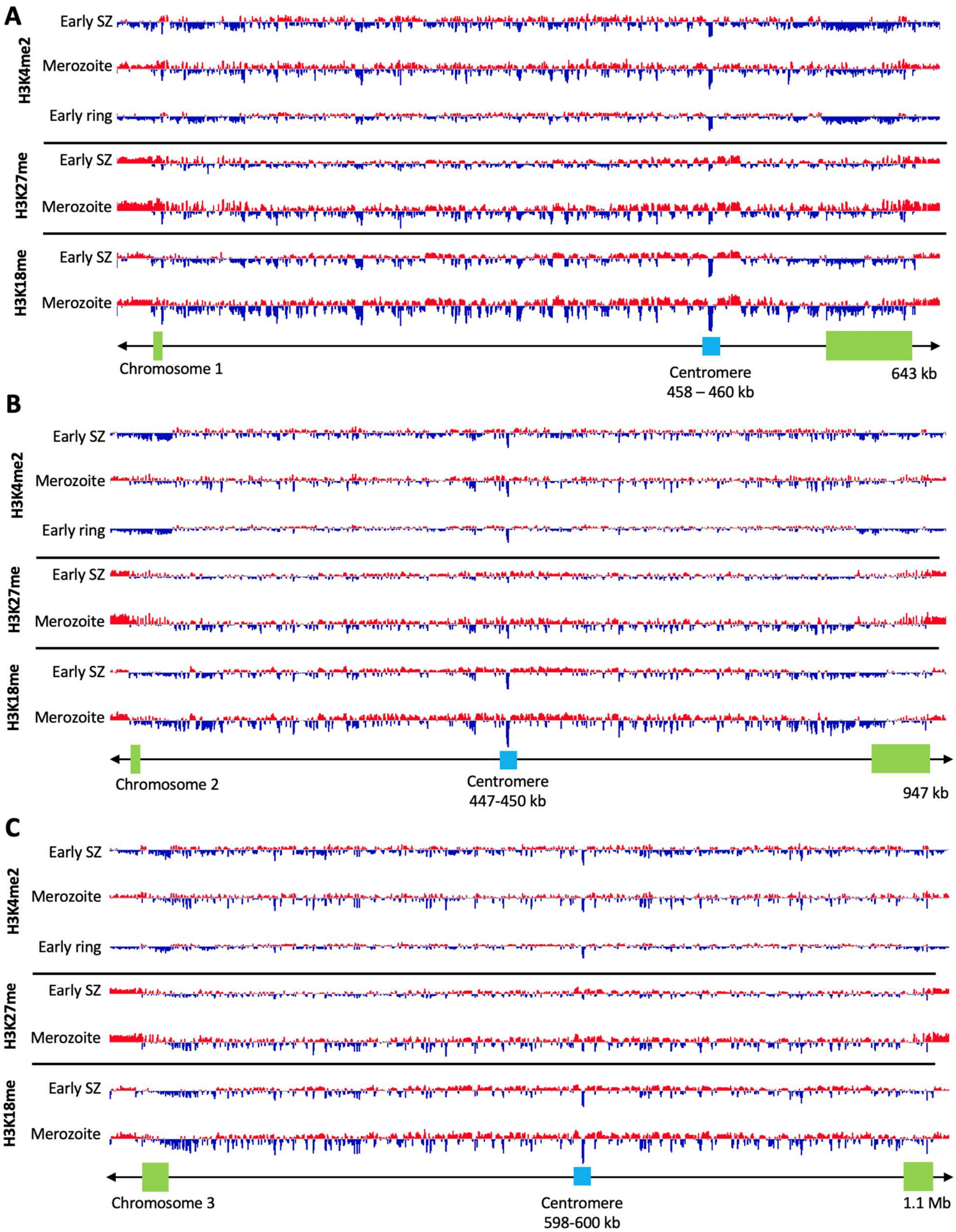

Figure S9 – part 1

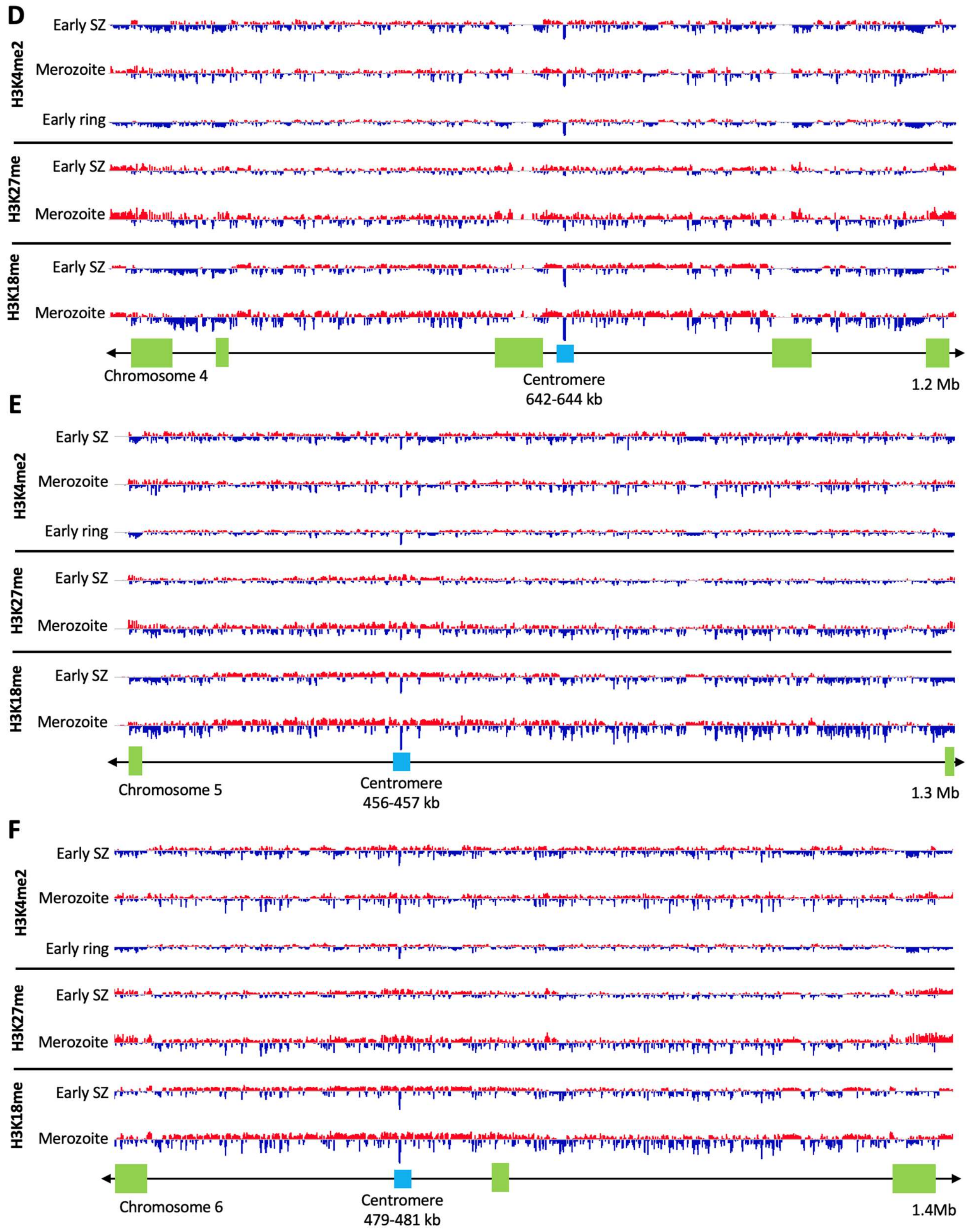

Figure S9 – part 2

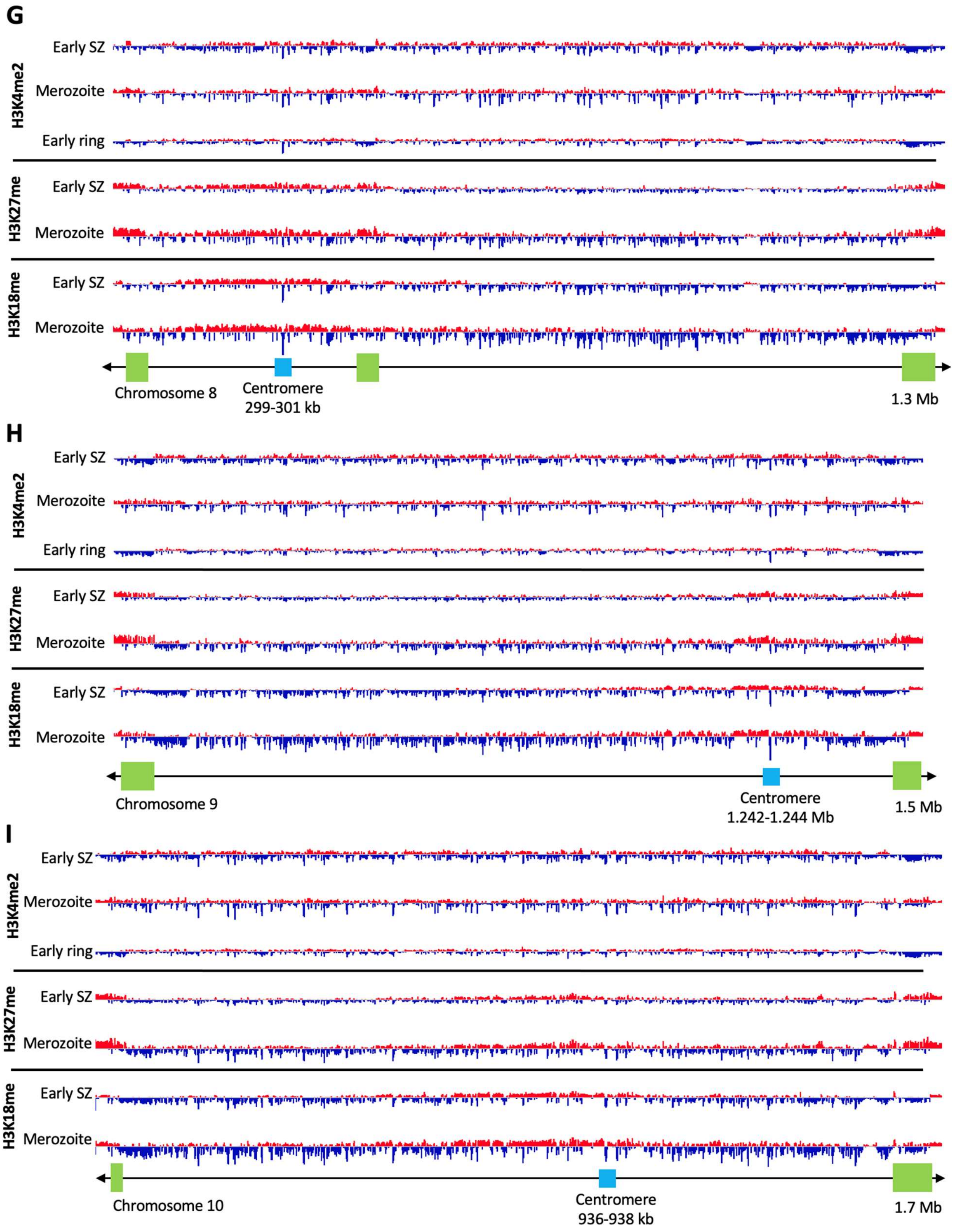

Figure S9 – part 3

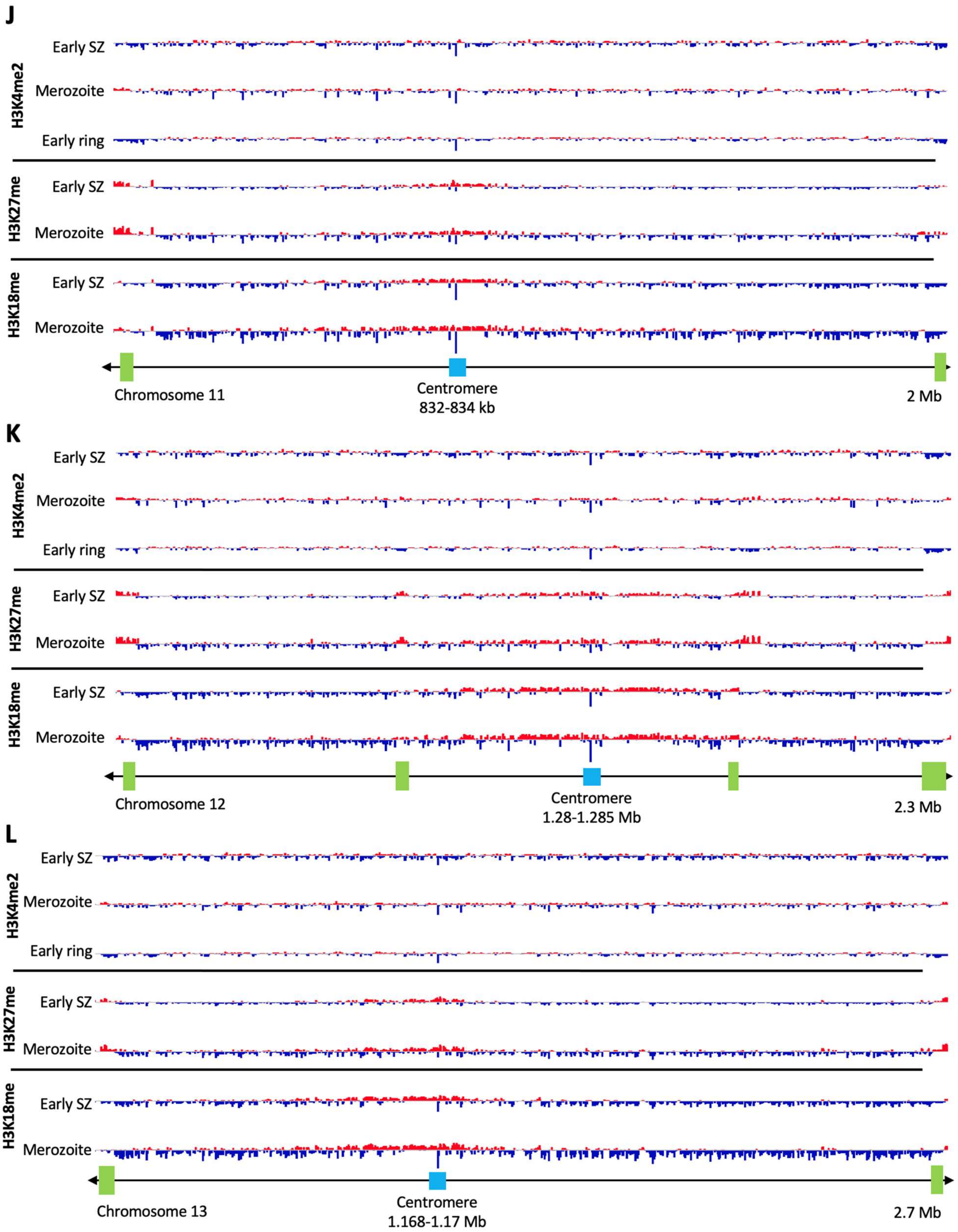

Figure S9 – part 4

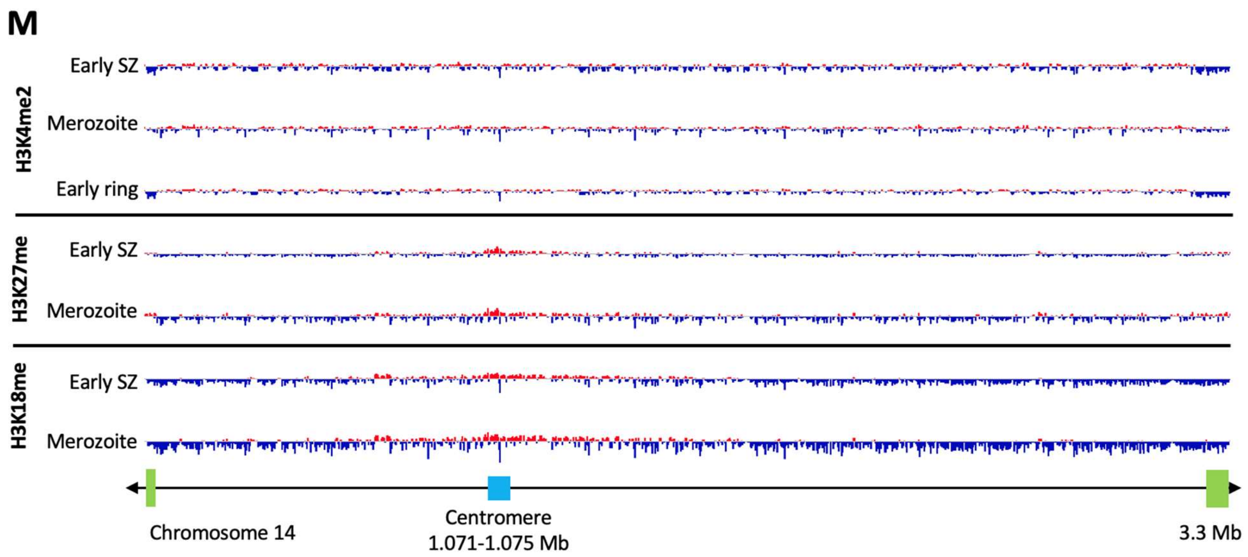

Figure S9. Distribution of methylation marks across all chromosomes. A-M) Log_2(ChIP/input) enrichment of H3K4me2, H3K27me, and H3K18me across each chromosome (except chromosome 7, which is shown in Figure 4) in early schizonts (SZ, 40 hpi), merozoites, and early rings (4 hpi, only for H3K4me2). Centromeres (blue boxes) and virulence gene clusters (green boxes) are indicated. Data range for ChIP tracks is -8 to 4.

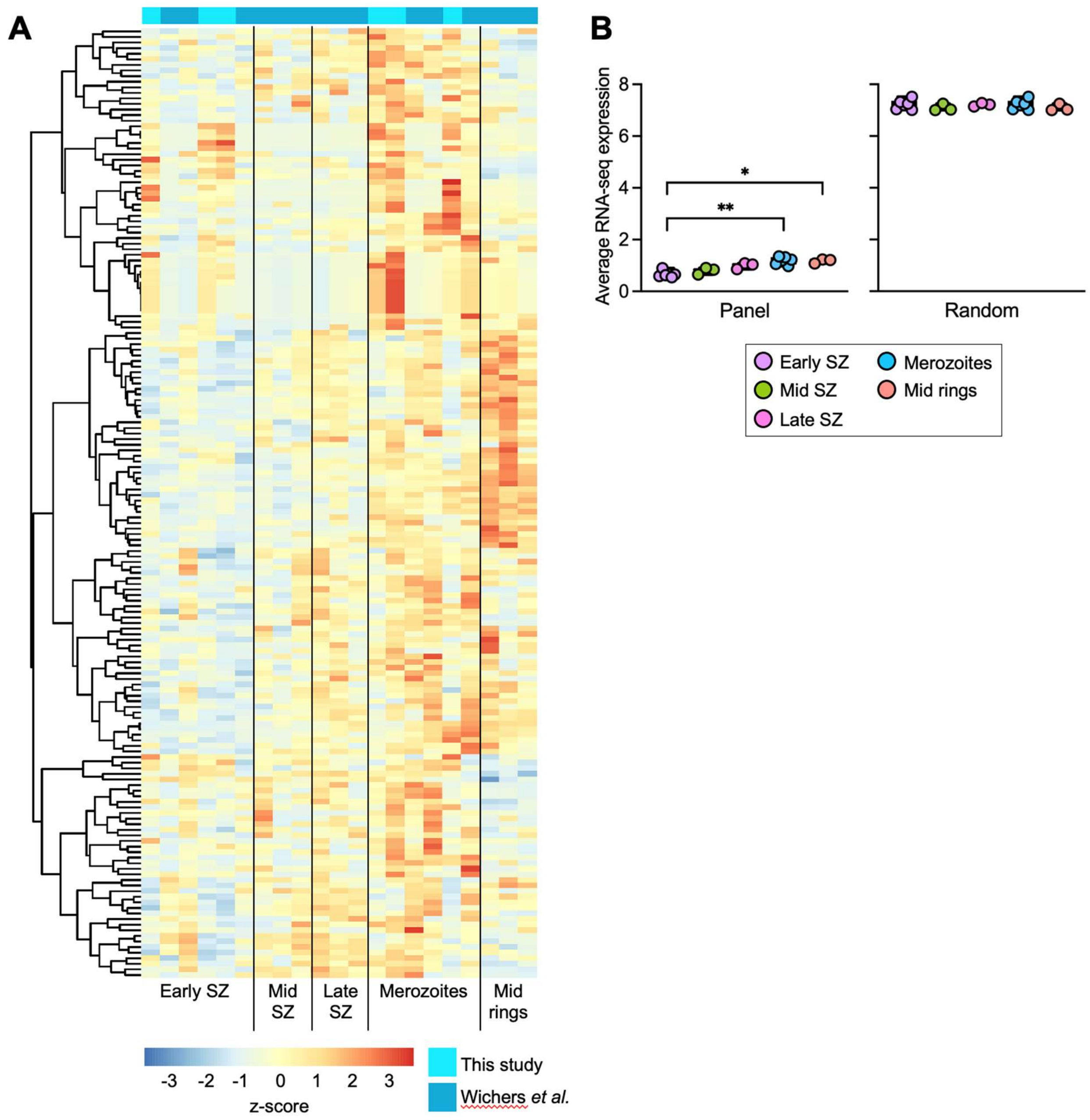

Figure S10. Expression of genes with enrichment of H3K4me2 in merozoites. A) Heatmap depicting DESeq2 normalized gene expression of genes with H3K4me2 enrichment in merozoites (n=170). **B)** Boxplots showing average expression of genes with H3K4me2 enrichment in merozoites compared to early schizonts and a set of randomly selected genes (n=99). Differences in gene expression were tested for significance using a one-way Kruskal-Wallis test. P-values indicated in the graphs are from Dunn's post hoc tests. *, $p<0.05$; **, $p<0.01$.

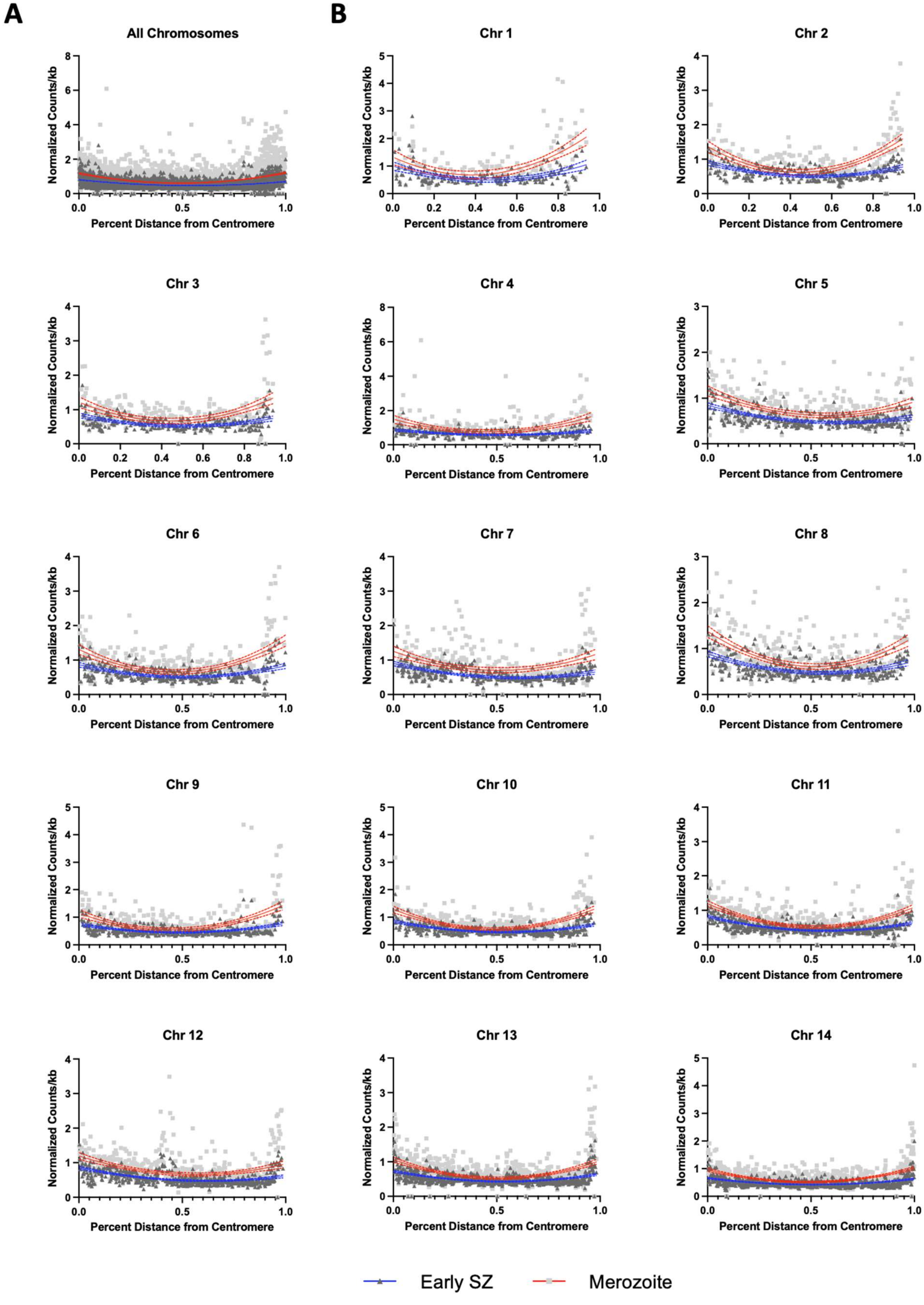

A
All Chromosomes
B
Chr 1
Chr 2
Chr 3
Chr 4
Chr 5
Chr 6
Chr 7
Chr 8
Chr 9
Chr 10
Chr 11
Chr 12
Chr 13
Chr 14
Normalized Counts/kb
Percent Distance from Centromere
Early SZ
Merozoite

Figure S11. Distribution of H3K18me across the chromosome. A-B) Distribution of H3K18me across **A)** all chromosomes and **B)** each chromosome individually. Enrichment shown as ChIP counts per gene length (kb) normalized to input values. Chromosomes depicted as percent distance from centromere with the centromere located at 0 and both telomeres located at 1. Line of best fit as calculated by nonlinear regression analysis indicated by solid lines The 95% confidence interval of the line of best fit is indicated by colored dashed lines.

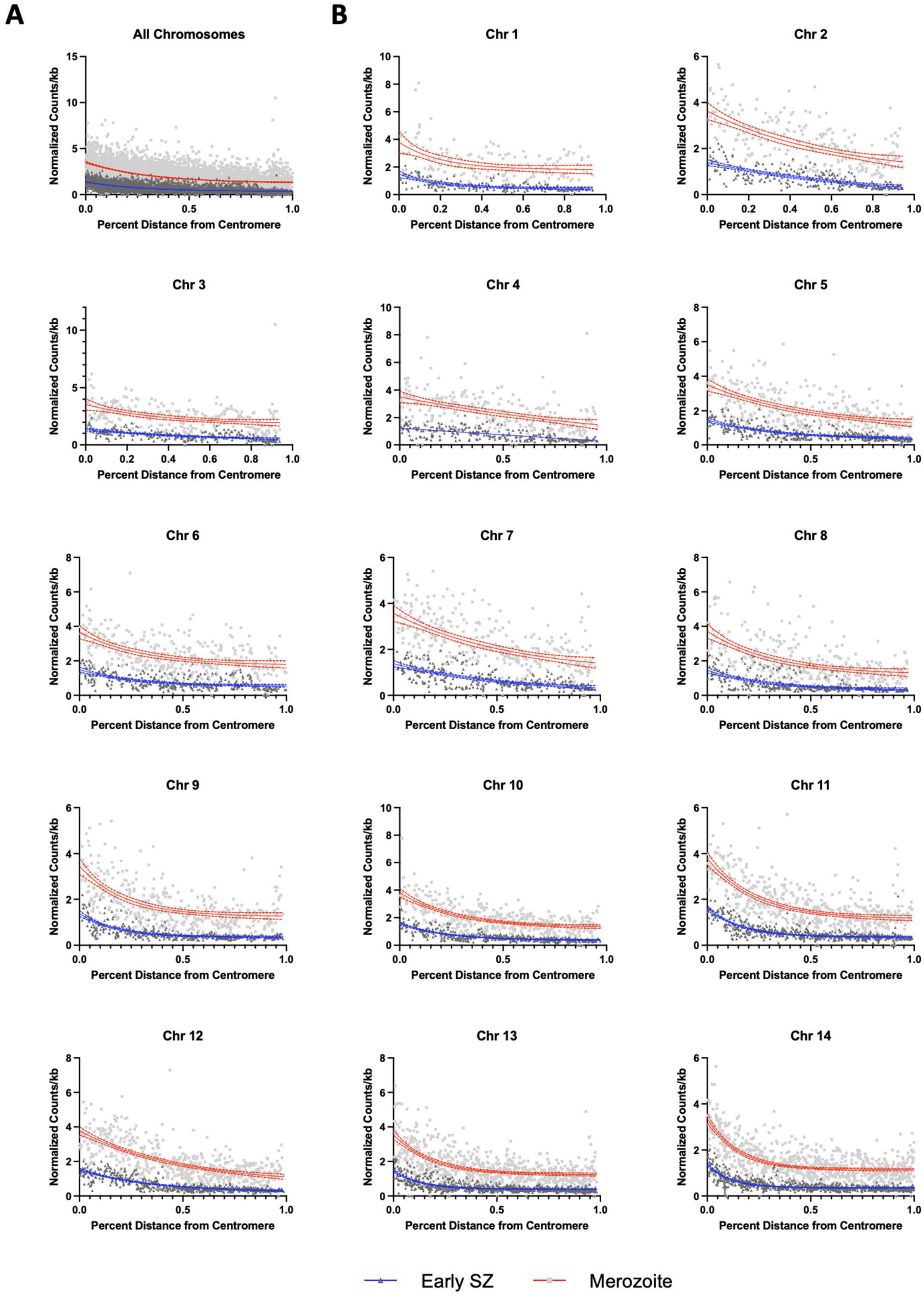

A
B
All Chromosomes
Chr 1
Chr 2
Chr 3
Chr 4
Chr 5
Chr 6
Chr 7
Chr 8
Chr 9
Chr 10
Chr 11
Chr 12
Chr 13
Chr 14
Normalized Counts/kb
Percent Distance from Centromere
Early SZ
Merozoite

Figure S12. Distribution of H3K27me across the chromosome. A-B) Distribution of H3K27me across **A)** all chromosomes and **B)** each chromosome individually. Enrichment shown as ChIP counts per gene length (kb) normalized to input values. Chromosomes depicted as proportional distance from centromere with the centromere located at 0 and both telomeres located at 1. Line of best fit as calculated by nonlinear regression analysis indicated by solid lines The 95% confidence interval of the line of best fit is indicated by colored dashed lines.

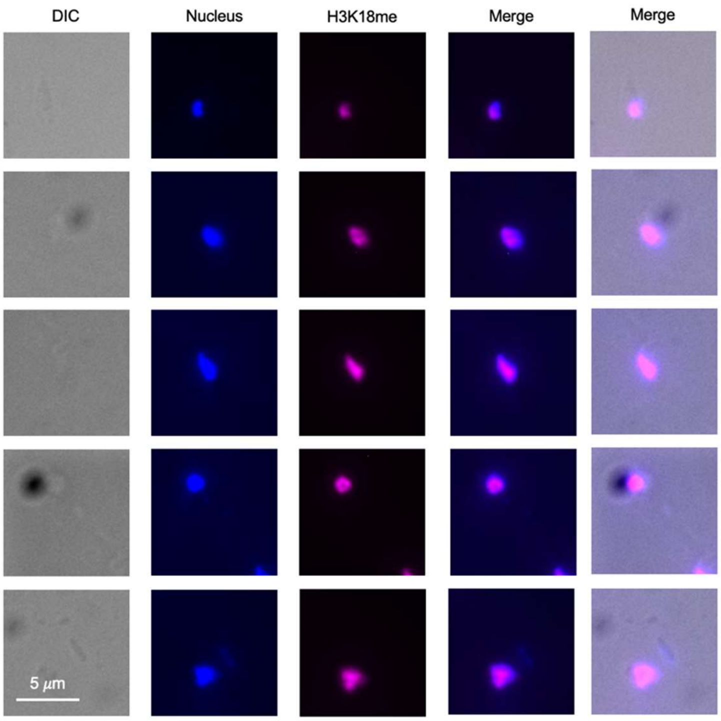

Figure S13. H3K18me immunofluorescence staining in rings.

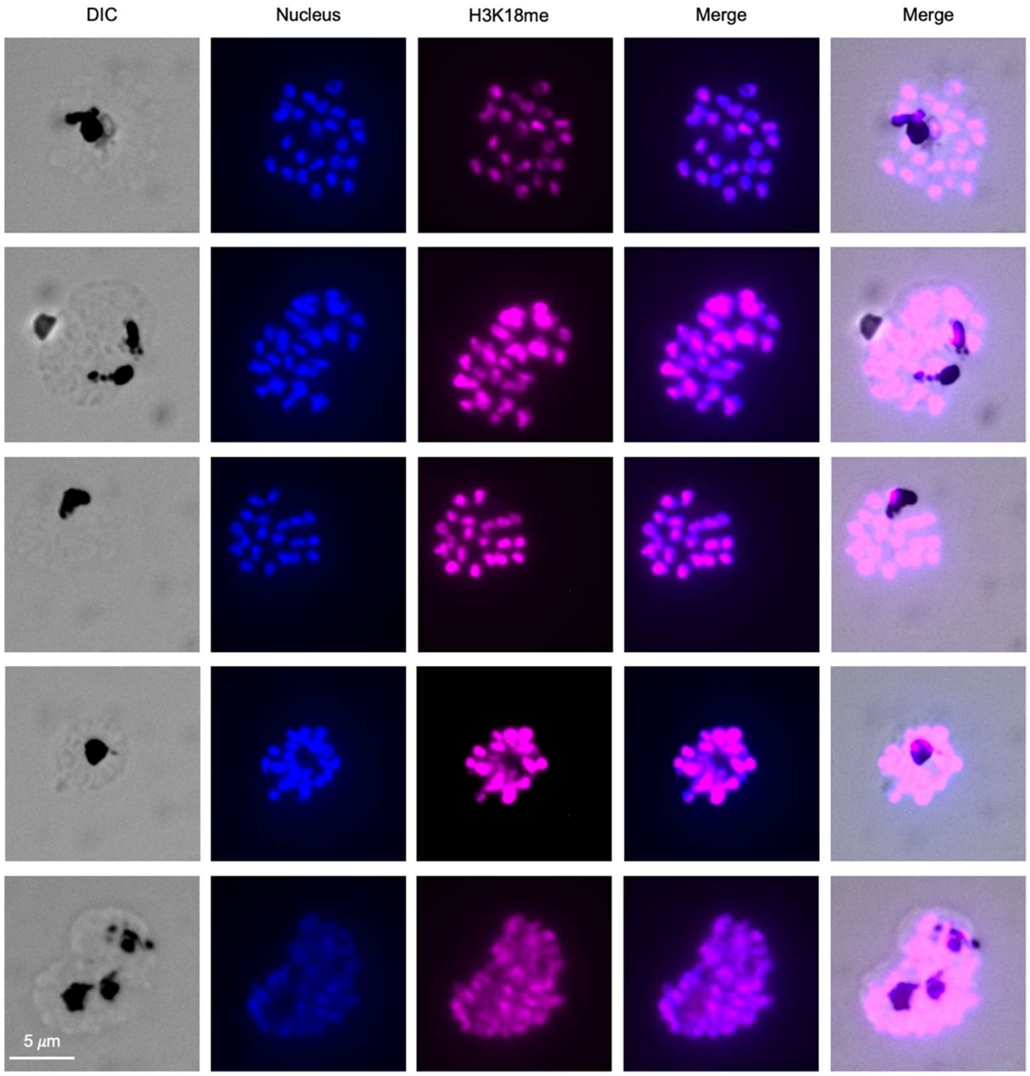

Figure S14. H3K18me immunofluorescence staining in schizonts.

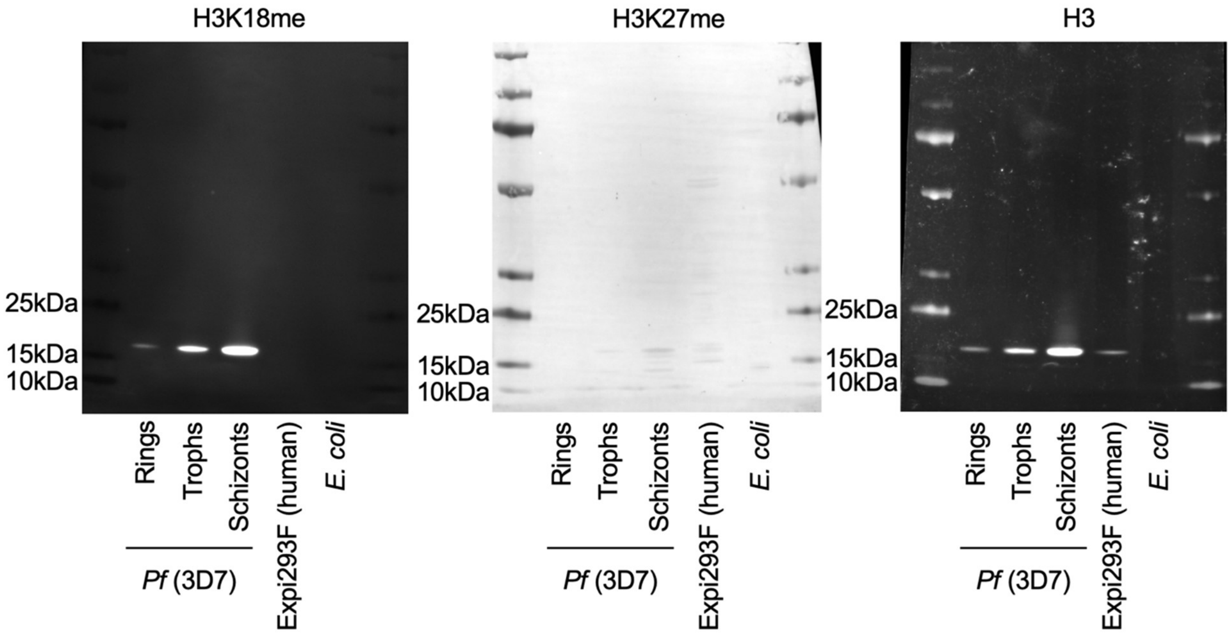

Figure S15: Western blot analysis of H3K18me and H3K27me during the IDC. Shown are the full blots of data presented in Figure 4H.

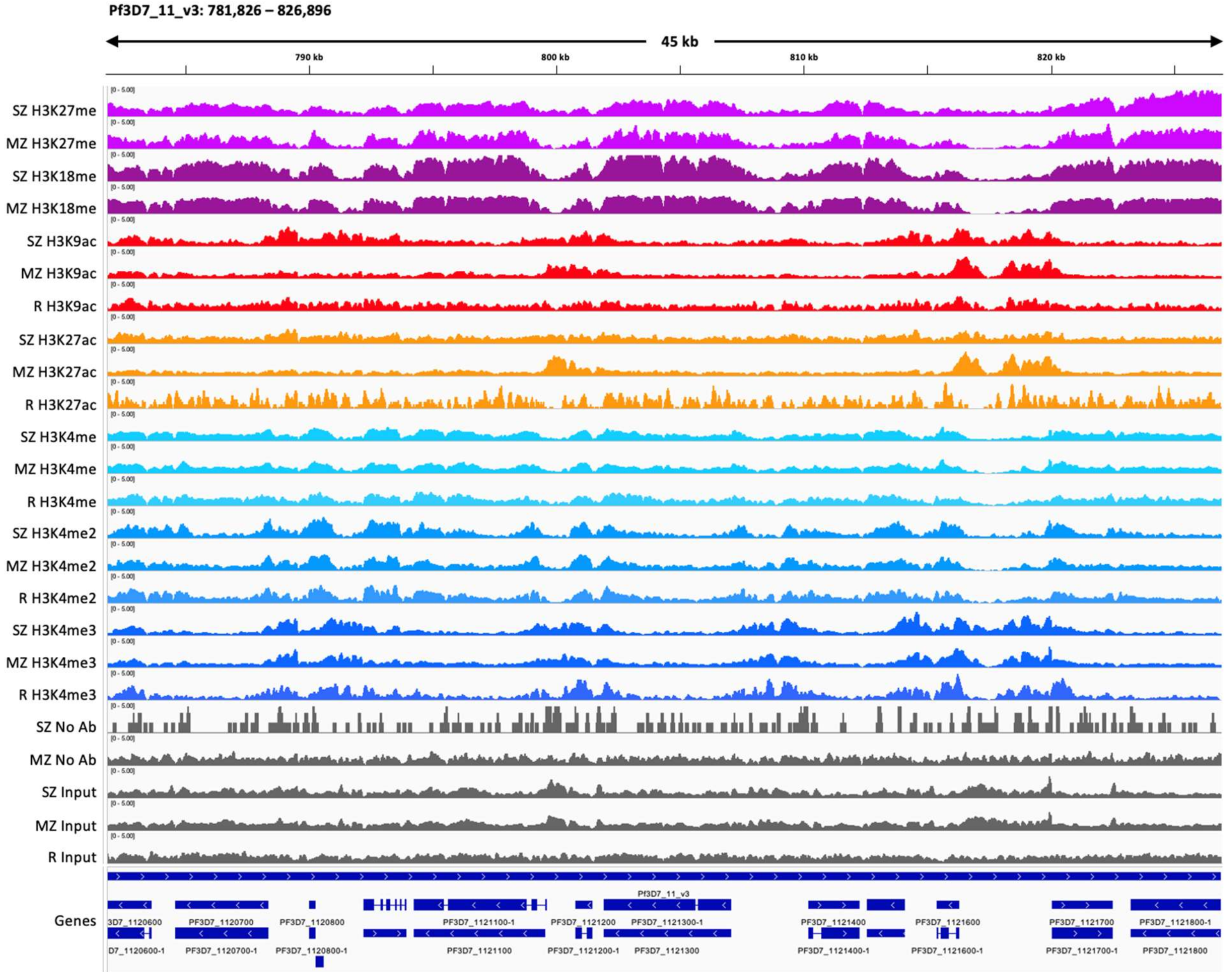

Figure S16. Unnormalized ChIP tracks for all marks assessed in this study. Shown are track for H3K4me, H3K4me2, H3K4me3, H3K9ac, H3K27ac, H3K27me, H3K18me, input, and No antibody control (No Ab) for schizonts (SZ), merozoites (MZ), and rings (R) for a representative region of chromosome 11. Data range of ChIP tracks is indicated in brackets above each track. Locations of genes are indicated at the bottom of the figure.
