# Supplementary material for: Histone modification analysis reveals common regulators of gene expression in liver and blood stage merozoites of Plasmodium parasites
Source: Epigenetics Chromatin. 2023 Jun 15;16:25. doi: 10.1186/s13072-023-00500-y (PMC10268464; doi:10.1186/s13072-023-00500-y)
Supplement: Supplementary file 3 — Additional file 3. Additional methods. [file 13072_2023_500_MOESM3_ESM.zip › Suppl_Methods/Magnetic purification of parasites - large column.docx]

**Magnetic Purification of Parasites – Large Column**

Materials:

| **Item** | **Preparation** | **Storage** |
| --- | --- | --- |
| SuperMACS magnet |  |  |
| MACS D Column |  |  |
| 60 mL syringe |  |  |
| Waste bins (2) |  |  |
| Deionized water |  |  |
| 100% ethanol |  | Flammable chemicals cabinet |
| 22 G needle |  |  |
| 1 mM E-64  Epoxysuccinyl-L-leucylamido(4-guanidino)butane |  | Aliquots in -20°C |
| Incomplete medium (~750 mL) | See Medium for *P. falciparum* Culture protocol | 4°C |
| 1.2 um/25 mm syringe filter |  |  |
| Complete medium | See Medium for *P. falciparum* Culture protocol | 37°C bead bath or 4°C |
| MACS LS magnetic column  HEMOZOIN REMOVAL ONLY |  |  |
| MACS magnet  HEMOZOIN REMOVAL ONLY |  |  |

Notes:

- DO NOT LET THE COLUMN RUN DRY DURING THE PROCEDURE
- Isolations should be started when most parasites are in the early schizont stage (4-6 nuclei visible)
- When isolating merozoites, a good yield is obtained when greater than 50% of parasites have formed membrane enclosed merozoites following E64 treatment

General Timeline for isolating merozoites:

Day 1, 5:00/5:30pm: Sorbitol synchronization

Day 2, 7am: Sorbitol synchronization

Day 3, 10:30am: Magnetic purification, outlined below – schizont collection

Day 3, 6:30 pm: Merozoite collection, outlined below

Protocol:

Preparation (New Column)

1. Remove yellow cap from the separation column. Save the cap
2. Attach 3-way stopcock to column at port A
3. Fill a 60 mL syringe with 70% ethanol and attach to port B
4. Turn 3-way stopcock to “fill” position
5. Move the gap of the SuperMACS to approximately 3 cm width using the side wheel. Place assembled column in MACS separator with 3-way stopcock in adjustment and secure with lever
6. Move the column into the magnetic field by turning the handle
7. Place a waste bin under the column to collect the flowthrough
8. Fill the column from the bottom with 70% ethanol from the syringe until the solution reaches the reservoir
9. Continue with protocol below

Preparation (Old Column)

1. Turn the 3-way stopcock to the “run” position and rinse the column by filling from the top with incomplete medium. Allow medium to run into the column until it reaches the top white filter, then add fresh medium. Rinse with 200 mL of incomplete medium. When complete turn stopcock to “closed” position
2. Fill a 60 mL syringe with 50 mL of incomplete parasite medium. *Remove the plunger and attach yellow cap from above. Fill the syringe, put the plunger in until the rubber seals the edge, turn the syringe around. Remove the yellow cap and push the plunger until all air is removed.*
3. Attach the filled syringe to port B. Leave the syringe attached except when refilling
4. Turn stopcock to “fill”
5. Fill the column completely with incomplete medium from the syringe
6. Turn the. Stopcock to “run” and let the medium run into the column until it reaches the white filter
7. Turn the stopcock to “closed”
8. Repeat this step until the syringe is emptied
9. Cut the tip off the tip of the plastic sheath of a 22G needle (flow restrictor) using pliers. Leave the plastic sheath in place and attach the flow restrictor to port C of the 3-way stopcock
10. Refill the syringe with 50 mL of incomplete medium

Isolation of iRBCs

1. Remove 10 mL of media from each flask if isolating from multiple flasks
2. Make a smear
3. Apply the culture onto the column with the flow restrictor attached
4. Turn the stopcock to “run and allow the cell suspension to penetrate the matrix of the column
5. Wash the column with incomplete media from the top until the flowthrough is barely red – usually about 100 mL
6. Turn the stopcock to “fill”
7. Fill the column with incomplete medium from the syringe
8. Turn the stopcock to “run” and let the medium run into the column until it reaches the top white filter
9. Turn the stopcock to “closed”
10. Repeat until all the media in the syringe is used
11. Wash the column with 100 mL of incomplete medium from the top

Elution of iRBCs

1. Move the column out of the magnetic field by turning the handle
2. Place a 50 mL collection tube under the column and prepare a second 50 mL tube
3. Elute iRBCs off the column by adding incomplete medium until the elution runs clear – usually about 60 mL
4. Centrifuge the tube for at 250 x g for 5 minutes at RT (acc=9, dec=1)
5. Remove the supernatant and resuspend the pellet in 3 mL of complete medium – this is the stopping point for isolating schizonts.
6. To isolate merozoites, transfer the parasites to a 25-cm^2^ flask
7. Add 30 uL E64 and place the flask upright in the incubator
8. Incubate for 8 hours at 37°C

Merozoite Isolation

1. Transfer the suspension of iRBCs to a 15 mL tube
2. Centrifuge at 1,900 x g for 8 minutes at RT (acc=9, dec=1)
3. Remove the supernatant and make a smear
4. Resuspend the parasite in 4 mL of incomplete medium
5. Filter the resuspended iRBCs through a 1.2 um/25mm syringe filter (note that these are not the filters used for sterilization). Collect the filtrate in a 15 mL tube
6. Centrifuge at 4,000 x g for 10 minutes at RT (acc=9, dec=1)
7. Remove the supernatant

Regeneration of the Column

1. Perform regeneration while the column is NOT in the magnetic field
2. Remove the flow restrictor
3. Place a waste bin under the column
4. Rinse the column from the top with 150 mL of deionized water
5. Perform one fill from the bottom with the syringe of deionized water
6. Rinse the column from the top with 150 mL of 100% ethanol
7. Store the column in 100% ethanol, making sure that the column is filled with ethanol to slightly above the top white filter

Waste:

- All waste can be collected in the waste container under the hood which contains 34 mL bleach per 500 mL waste. Waste should sit for at least 30 minutes following the last addition to be disposed of by pouring down the sink.
