# Supplementary material for: Histone modification analysis reveals common regulators of gene expression in liver and blood stage merozoites of Plasmodium parasites
Source: Epigenetics Chromatin. 2023 Jun 15;16:25. doi: 10.1186/s13072-023-00500-y (PMC10268464; doi:10.1186/s13072-023-00500-y)
Supplement: Supplementary file 3 — Additional file 3. Additional methods. [file 13072_2023_500_MOESM3_ESM.zip › Suppl_Methods/Magnetic purification of parasites - small column.docx]

**Magnetic Purification of Parasites – Small Column**

Materials:

| **Item** | **Preparation** | **Storage** |
| --- | --- | --- |
| MACS LS magnetic column |  |  |
| MACS magnet |  |  |
| Complete medium | See Medium for *P. falciparum* Culture protocol | 37°C bead bath or 4°C |
| 1 mM E-64  Epoxysuccinyl-L-leucylamido(4-guanidino)butane |  | Aliquots in -20°C |
| Incomplete medium | See Medium for *P. falciparum* Culture protocol | 4°C |
| 1.2 um/25 mm syringe filter |  |  |
| Deionized water |  |  |
| 100% ethanol |  | Flammable chemicals cabinet |

Notes:

- LS column can be reused 10 times
- Can isolate parasites from 3-4 flasks per column
- Isolations should be started when most parasites are in the early schizont stage (4-6 nuclei visible)
- When isolating merozoites, a good yield is obtained when greater than 50% of parasites have formed membrane enclosed merozoites following E64 treatment

General Timeline:

Day 1, 5:00/5:30pm: Sorbitol synchronization

Day 2, 7am: Sorbitol synchronization

Day 3, 10:30am: Magnetic purification, outlined below – schizont collection

Day 3, 6:30 pm: Merozoite collection, outlined below

Protocol:

Preparation

1. Attach an LS magnetic column to a MACS magnet
2. Place a 15 mL tube for waste collection under the column
3. Apply 5 mL of parasite medium to the column to equilibrate

Isolation of infected RBCs (iRBCs)

1. Pellet culture at 250 x g for 5 minutes at RT (acc=9, dec=1)
2. Remove the supernatant and make a smear
3. Resuspend the pellet in complete medium at 25% hematocrit. *For a 75-cm^2^ flask, add 3 mL of complete media.*
4. Place a clean 15 mL tube for flowthrough collection under the column
5. Add the resuspended culture to the column and let it drip through
6. Apply the flowthrough to the column once more. *Remember to place the flowthrough tube back under the column before applying the flowthrough.*
7. Add 0.75 mL of complete medium to the column and collect the flowthrough in the same tube
8. Place the waste tube under the column and wash the column with 2 mL of complete medium
9. Remove the column from the magnet and place in a 25-cm^2^ flask for the collection of iRBCs bound to the column
10. Apply 1 mL of complete medium to the column to elute bound iRBCs – this is the stopping point for isolating schizonts (after repeating the elution as described in step 12).
11. To isolate merozoites, add 10 uL of 1 mM E64 for each 1 mL of eluted iRBC suspension. Mix well. Place flask in the bead bath while continuing with the isolation protocol
12. Repeat the isolation of iRBCs using the RBC flowthrough 2 more times and collect all elutions in the same flask
13. After the last elution, place the flask upright in the incubator and incubate for 8 hours

Merozoite Isolation

1. Transfer the suspension of iRBCs to a 15 mL tube
2. Centrifuge at 1,900 x g for 8 minutes at RT (acc=9, dec=1)
3. Remove the supernatant and make a smear
4. Resuspend the parasite in 4 mL of incomplete medium
5. Filter the resuspended iRBCs through a 1.2 um/25mm syringe filter (note that these are not the filters used for sterilization). Collect the filtrate in a 15 mL tube
6. Centrifuge at 4,000 x g for 10 minutes at RT (acc=9, dec=1)
7. Remove the supernatant

Re-use of LS Columns

1. Place the column in an empty waste tube
2. Apply 3 mL of deionized water to the column and let it drip through
3. Apply 3 mL of 100% ethanol to the column and let it drip through
4. Use the plunger to remove the remaining ethanol from the column
5. Store the clean and dried column and the plunder in 2 separate 50 mL tubes at RT

Waste:

- All waste can be collected in the waste container under the hood which contains 34 mL bleach per 500 mL waste. Waste should sit for at least 30 minutes following the last addition to be disposed of by pouring down the sink.
