# Supplementary material for: Histone modification analysis reveals common regulators of gene expression in liver and blood stage merozoites of Plasmodium parasites
Source: Epigenetics Chromatin. 2023 Jun 15;16:25. doi: 10.1186/s13072-023-00500-y (PMC10268464; doi:10.1186/s13072-023-00500-y)
Supplement: Supplementary file 3 — Additional file 3. Additional methods. [file 13072_2023_500_MOESM3_ESM.zip › Suppl_Methods/Medium for parasite culture.docx]

**Medium for *P. falciparum* Culture**

Incomplete Medium

Per bottle:

- 500 mL RPMI (no glutamine, no phenol red)
- 2.5 mL gentamicin (10 mg/mL)
- 22.5 mL HEPES (1 M)
- 14 mL sodium bicarbonate (7.5%)
- 1.5 mL NaOH (1 M)
- 6 mL L-glutamine (200 mM) – do not reuse aliquots
- 9.52 mg hypoxanthine (6.53 mg/mL) –**MAKE FRESH**, dilute in NaOH, filter sterilize

Notes:

- ALL SOLUTIONS MUST BE STERILE – filtered with syringe and 0.22 um filter or 0.22 um bottle top filter
- Thaw L-glutamine in 37°C bead bath
- Can be stored at 4°C for 1 month
- When making 4 bottles, prepare hypoxanthine: Dissolve 47.6 mg in 7.5 mL NaOH, filter sterilize

Protocol:

1. Add to each 500mL bottle of RPMI:

2.5 mL gentamicin

22.5 mL HEPES

14 mL sodium bicarbonate

1.5 mL NaOH with 9.52 mg of hypoxanthine. *Per bottle: dissolve 19.05 mg hypoxanthine in 3 mL NaOH.*

6 mL L-glutamine. Do not re-use aliquots

1. Final volume is 546.5 mL. Remove 46.5 mL from each bottle and place in 50 mL tube. Store in 4°C, labeled “Incomplete media 500 mL.”

Complete Medium

Notes:

- Storage at 37°C for extended periods of time can damage complete medium. Aliquot into 50 mL tubes to warm up while storing large bottle at 4°C when culturing small numbers of parasites. In general, make aliquots if it takes longer than 1 week to use 500 mL of complete media. Can be stored at 4°C for 1 month.
- Thaw human serum at 4°C overnight or for ~1 hr at 37°C.
- Pre-warm incomplete media to 37°C to improve filtering.
- Each 0.22 um bottle top filter can be used to filter up to 1 L of complete medium.

Protocol:

1. Add 10% (v/v) heat-inactivated human serum (30 minutes at 56°C) human serum. *Add 50 mL human serum to one 500 mL bottle of incomplete media.*
2. Filter sterilize using a 0.22 um bottle top filter.

Chemical Information

| **Item** | **Product Number** | **[Stock]** | **[Incomplete Final]** | **[Complete Final]** | **Storage** |
| --- | --- | --- | --- | --- | --- |
| Gentamicin | Gibco  15710064 | 10 mg/mL | 45 µg/mL | 41 µg/mL | RT |
| HEPES | Fisher  BP299100 | 1 M | 40 mM | 37 mM | Chemical: RT  Solution: 4°C |
| Sodium Bicarbonate | Corning  25-035-CI | 7.5% (wt/vol), 75 g/L | 1.9 mg/mL | 1.7 mg/mL | Chemical: RT  Solution: 4°C |
| NaOH | Fisher  SS266-1 | 1 M | 2.7 mM | 2.4 mM | RT |
| Hypoxanthine | Alfa Aesar  A11481-06 | 6.35 mg/mL (when diluted in NaOH) | 17 µg/mL | 15 µg/mL | 4°C, in bag to reduce moisture |
| L-glutamine | Corning  25005CI | 200 mM | 2.1 mM | 1.9 mM | -20°C, aliquots |
| Human Serum | Valley Biomedical  HP1022 | N/A | N/A | 9.1% | -20, aliquots |
