# Supplementary material for: Histone modification analysis reveals common regulators of gene expression in liver and blood stage merozoites of Plasmodium parasites
Source: Epigenetics Chromatin. 2023 Jun 15;16:25. doi: 10.1186/s13072-023-00500-y (PMC10268464; doi:10.1186/s13072-023-00500-y)
Supplement: Supplementary file 3 — Additional file 3. Additional methods. [file 13072_2023_500_MOESM3_ESM.zip › Suppl_Methods/Parasite chromatin immunoprecipitation.docx]

**Parasite Chromatin Immunoprecipitation**

Materials:

| **Item** | **Preparation** | **Storage** |
| --- | --- | --- |
| 1X PBS |  | RT |
| 10X Protease inhibitors, **MAKE FRESH** | 1 tablet dissolved in 1 mL 1X PBS | 4°C |
| 10X Phosphatase inhibitors, **MAKE FRESH** | 1 tablet dissolved in 1 mL 1X PBS | 4°C |
| Ice |  | Ice machine |
| 10% NP-40/IGEPAL | 5 mL NP-40 + 45 mL water | RT |
| 26 G 5/8 needle |  | RT |
| 1 mL syringe |  | RT |
| Covaris microTUBE AFA fiber pre-slit snap-cap (6x16 mm) |  |  |
| 5 M NaCl | 14.6 g NaCl + 50 mL water | RT |
| 1 mg/mL BSA | Dilute 35% BSA 1:350 in water, 1.4 uL BAS per 500 uL water | 4°C |
| Nuclear extraction buffer, **MAKE FRESH** | See below |  |
| Shearing buffer | See below | RT |
| ChIP dilution buffer | See below | RT |
| High salt wash | See below | RT |
| Low salt wash | See below | RT |
| LiCl wash | See below | RT |
| TE buffer | See below | RT |
| Elution buffer | See below | RT |
| Protein A agarose/salmon sperm DNA | Vortex gently before use to resuspend agarose beads | 4°C |
| SparQ beads | Vortex gently before use to resuspend agarose beads, beads must be warmed to RT before use (30 min on bench) | 4°C |
| 0.5 M EDTA |  | RT |
| 1 M Tris pH 7.5 |  | RT |
| Proteinase K (800 U/mL) |  | -20°C |
| 80% ethanol, **MAKE FRESH** | 800 uL 100% ethanol + 200 uL water |  |
| Nuclease-free water |  | RT |

Buffers:

| **Nuclear Extraction Buffer** | **Amount (uL)** | **[Final]** |
| --- | --- | --- |
| 1M HEPES | 10 | 10 mM HEPES |
| 1M KCl | 10 | 10 mM KCl |
| 10 mM EDTA, pH 8 | 10 | 0.1 mM EDTA |
| 10 mM EGTA, pH 8 | 10 | 0.1 mM EGTA |
| 100 mM DTT | 10 | 1 mM DTT |
| 200 mM AEBSF | 2 | 0.5 mM AEBSF |
| 10X Protease Inhibitor | 100 | 1X |
| 10X Phosphatase Inhibitor | 100 | 1X |
| Water | 748 |  |
| Total | 1000 |  |

| **Shearing Buffer** | **Amount (uL)** | **[Final]** |
| --- | --- | --- |
| 10% SDS | 10 | 1% SDS |
| 10 mM EDTA, pH 8 | 100 | 1 mM EDTA |
| 1M Tris, pH 7.5 | 10 | 10 mM Tris |
| 10X Protease Inhibitor | 100 | 1X |
| 10X Phosphatase Inhibitor | 100 | 1X |
| Water | 680 |  |
| Total | 1000 |  |

| **ChIP Dilution Buffer** | **Amount (uL)** | **[Final]** |
| --- | --- | --- |
| 1M Tris, pH 8 | 300 | 30 mM |
| 0.5M EDTA | 60 | 3 mM |
| 10% SDS | 10 | 0.1% |
| NaCl | 0.175 g | 300 mM |
| Triton X-100 | 180 | 1.8% |
| Water | 9450 |  |
| Total | 10 mL |  |

| **ChIP Dilution Buffer with Inhibitors** | **Amount (uL)** | **[Final]** |
| --- | --- | --- |
| ChIP Dilution Buffer | 800 |  |
| 10X Protease Inhibitors | 100 | 1X |
| 10X Phosphatase Inhibitors | 100 | 1X |
| Total | 1000 |  |

| **High Salt Wash** | **Amount (uL)** | **[Final]** |
| --- | --- | --- |
| 10% SDS | 1000 | 1% SDS |
| 10% Triton X-100 | 1000 | 1% Triton |
| 0.5M EDTA | 40 | 2mM EDTA |
| 1M Tris pH 8 | 200 | 20mM Tris |
| 5M NaCl | 1000 | 500mM NaCl |
| H2O | 6760 |  |
| Total | 10000 |  |

| **Low Salt Wash** | **Amount (uL)** | **[Final]** |
| --- | --- | --- |
| 10% SDS | 1000 | 1% SDS |
| 10% Triton X-100 | 1000 | 1% Triton |
| 0.5M EDTA | 40 | 2mM EDTA |
| 1M Tris pH 8 | 200 | 20mM Tris |
| 1.5M NaCl | 1000 | 150mM NaCl |
| H2O | 6760 |  |
| Total | 10000 |  |

| **LiCl Wash** | **Amount (uL)** | **[Final]** |
| --- | --- | --- |
| 2.5M LiCl | 1000 | 0.25M LiCl |
| 10% NP-40 | 1000 | 1% NP-40 |
| 10% Na deoxycholate | 1000 | 1% Na deoxycholate |
| 0.5M EDTA | 20 | 1mM EDTA |
| 1M Tris pH 8 | 100 | 10mM Tris |
| H2O | 6880 |  |
| Total | 10000 |  |

| **TE** | **Amount (uL)** | **[Final]** |
| --- | --- | --- |
| 1M Tris-HCl pH 8 | 100 | 10mM Tris |
| 0.5M EDTA | 20 | 1mM EDTA |
| H2O | 9880 |  |
| Total | 10000 |  |

| **Elution** | **Amount (uL)** | **[Final]** |
| --- | --- | --- |
| 10% SDS | 100 | 1% SDS |
| 7.5% NaHCO3 | 112.4 | 0.1M NaHCO3 |
| H2O | 787.6 |  |
| Total | 1000 |  |

Notes:

- Use low binding tubes for all steps
- Buffers without protease and phosphatase inhibitors can be made in bulk and stored at RT

General timeline:

Day 1: Nuclear extraction, sonication, ChIP Day 1

Day 2: ChIP Day 2 (5.5 hours)

Day 3: ChIP Day 3

Protocol:

Nuclear Extraction and Sonication

1. Resuspend parasites in 1 mL of 1X PBS with protease inhibitors
2. Centrifuge at 2100 x g for 5 minutes at 4°C
3. Remove the supernatant and resuspend pellet in 1 mL of nuclear extraction buffer
4. Incubate on ice for 35 minutes
5. Add 25 uL of 10% NP-40/IGEPAL to obtain a final concentration of 0.25% IGEPAL
6. Homogenize solution by passing through a 26G 5/8 needle and 1 mL syringe 7x
7. Centrifuge at 2100 x g for 20 minutes at 4°C
8. Remove the supernatant and resuspend in up to 130 uL of shearing buffer and transfer to a Covaris microTUBE. *The total volume in the microTUBE can not exceed 130 uL, so adjust the amount of shearing buffer added based on the estimated pellet size.*
9. Shear chromatin with Covaris M220 sonicator using the settings below:

| Duty Cycle | 5% |
| --- | --- |
| Peak Incident Power | 75W |
| Cycles per Burst | 200 |
| Temp | 6°C |
| Time | 300 seconds |

*When sonicating multiple samples, leave samples on ice when not in the sonicator.*

1. Transfer the sonicated chromatin to a 1.5 mL tube
2. Wash the microTUBE with 80 uL of shearing buffer and transfer to the 1.5 mL tube. *Total volume should be 200 uL, adjust the amount of shearing buffer used in the wash to bring the total to 200.*
3. Centrifuge the sample at 16,800 x g for 10 minutes at 4°C
4. Transfer the supernatant to a new tube. *This is your sheared chromatin. If collecting an input sample, collect 30 uL from this supernatant and store in a new 1.5 mL tube to store at -70°C until library preparation. At this stage, samples can be stored at -70°C.*

ChIP Day 1

1. Wash protein A agarose/salmon sperm DNA 3x with ChIP dilution buffer without inhibitors by adding 500 uL of dilution buffer, mixing, centrifuging at 100 x g for 1 min at RT, then discarding the supernatant being careful not to disturb the agarose pellet
2. Dilute chromatin 1:1 in ChIP dilution buffer with inhibitors
3. Add 50 uL of protein A agarose per 1 mL of chromatin suspension to pre-clear the chromatin suspension with protein A agarose/salmon sperm DNA (50% slurry) for 1 hour at 4°C with agitation. *Place rotator on bottom shelf of 4°C fridge for sample agitation.*
4. Pellet the agarose by centrifuging at 100 x g for 1 minute at RT
5. Move the supernatant to a 1.5 mL tube being careful not to disturb the agarose pellet
6. Add antibody to the pre-cleared chromatin suspension. *When using one chromatin sample for multiple antibodies, divide the chromatin suspension into multiple 1.5 mL tubes. Generally, use 1-2 ug of antibody.*
7. Incubate overnight at 4°C with agitation.

ChIP Day 2

1. Wash 25 uL of protein A agarose/salmon sperm DNA with ChIP dilution buffer without inhibitors. Centrifuge at 100 x g for 1 min at RT to pellet the agarose
2. Remove the supernatant
3. Add 500 uL of 1 mg/mL BSA to the agarose pellet to block the beads
4. Incubate for 1 hour at 4°C with agitation
5. Wash the protein A agarose beads 3x with ChIP dilution buffer without inhibitors
6. Add 25 uL of washed and blocked agarose to the chromatin suspension from Day 1
7. Incubate for 1 hour at 4°C with agitation
8. Centrifuge at 100 x g for 1 min at RT
9. Remove the supernatant and transfer to a 1.5 mL tube. Store at 4°C, labeled “ChIP supernatant” until sure that the ChIP has been successful. *This contains all the unbound chromatin fragments while the chromatin fragments bound by antibody are now bound to the agarose beads.*
10. Wash the agarose beads with 1 mL of low salt wash buffer for 15 minutes at 4°C with agitation. Centrifuge at 100 x g for 1 minute at RT and remove the supernatant
11. Repeat the low salt wash
12. Wash the agarose beads with 1 mL of high salt wash buffer for 15 minutes at 4°C with agitation. Centrifuge at 100 x g for 1 minute at RT and remove the supernatant
13. Repeat the high salt wash
14. Wash the agarose beads with 1 mL of LiCl wash buffer for 15 minutes at 4°C with agitation. Centrifuge at 100 x g for 1 minute at RT and remove the supernatant
15. Repeat the LiCl wash
16. Wash the agarose beads with 1 mL of TE buffer for 15 minutes at RT with agitation. Centrifuge at 100 x g for 1 minute at RT and remove the supernatant
17. Repeat the TE wash
18. Add 125 uL elution buffer to the pelleted beads to elute the protein complex from the antibody
19. Incubate for 15 minutes at RT with agitation
20. Centrifuge the sample at 100 x g for 1 minute at RT
21. Transfer the supernatant to a new 1.5 mL tube
22. Repeat the elution and combine in the same 1.5 mL tube to obtain 250 uL of total elution
23. Add 27.5 uL of 5 M NaCl to the elution
24. Incubate at 45°C in the water bath overnight to reverse the crosslinking

ChIP Day 3

1. Add 7.5 uL of Rnase A to the ChIP sample
2. Incubate for 30 minutes at 37°C
3. Add:

0.5 M EDTA 5 uL

1 M Tris (pH 7.5) 10 uL

Proteinase K (800 U/mL) 1 uL

1. Incubate for 2 hours at 45°C
2. Purify DNA using the SparQ beads. Use 600 uL of beads (2 volumes). *Use low binding tubes and low binding pipette tips.*

- Add 600 uL of beads to the ChIP sample and mix by pipetting gently
- Incubate for 5 minutes at RT on the bench
- Incubate for 5 minutes on the magnet
- Remove the supernatant with the tube on the magnet being careful to not touch the beads
- Add 200 uL of 80% ethanol
- Incubate for 30 seconds
- Remove the supernatant
- Repeat the ethanol wash
- Spin the beads of 10 seconds
- Place the tube back on the magnet and remove the remaining ethanol
- Air dry the beads for 5 minutes
- Remove the beads from the magnet
- Add 30 uL of nuclease free water
- Incubate for 5 minutes at RT
- Place the tube on the magnet and incubate for 5 minutes
- Transfer the supernatant to a new low binding tube

1. Store the ChIP samples at the -20°C until library preparation

Waste:

- 26G 5/8 needle and 1 mL syringe: Sharps container
- All other waste can be disposed of in a benchtop waste container.
