# Supplementary material for: Histone modification analysis reveals common regulators of gene expression in liver and blood stage merozoites of Plasmodium parasites
Source: Epigenetics Chromatin. 2023 Jun 15;16:25. doi: 10.1186/s13072-023-00500-y (PMC10268464; doi:10.1186/s13072-023-00500-y)
Supplement: Supplementary file 3 — Additional file 3. Additional methods. [file 13072_2023_500_MOESM3_ESM.zip › Suppl_Methods/Parasite crosslinking for ChIP.docx]

**Parasite Crosslinking for ChIP**

Materials:

| **Item** | **Preparation** | **Storage** |
| --- | --- | --- |
| 1X PBS, sterile, **COLD** | Use pre-bottled PBS | 4°C |
| Ice |  | Ice machine |
| 16% paraformaldehyde (PFA) |  | New vials at RT  Aliquots at -20°C |
| 1.25 M glycine |  | RT |

Notes:

- Use parasites that have been isolated by saponin isolation or merozoite isolation
- Isolated parasites must be resuspended in 1 mL of PBS.
- Paraformaldehyde must be warmed to RT before use
- Do not reuse PFA aliquots from the -20°C.

Protocol:

1. Pre-warm 16% paraformaldehyde (PFA) to RT by incubating on the bench
2. Add 62.5 uL 16% PFA to isolated parasites suspended in 1 mL of 1X PBS
3. Incubate at 37°C for 10 minutes, mixing by inversion occasionally
4. Add 100 uL of 1.25 M glycine to obtain a final concentration of 0.125 M
5. Incubate at 37°C for 5 minutes, mixing by inversion occasionally
6. Centrifuge at 4,000 x g for 5 minutes at 4°C
7. Pipette off the supernatant. *Dispose of supernatant in PFA Waste for disposal by EH&S.*
8. Wash pellet with 1 mL cold, sterile 1X PBS
9. Centrifuge at 4,000 x g for 5 minutes at 4°C
10. Remove the supernatant by pipetting
11. Repeat the wash with 1 mL cold, sterile 1X PBS two times
12. Remove the supernatant by pipetting
13. Store crosslinked parasites at -70°C

Waste:

- PFA: Collect supernatant containing PFA in PFA waste container. When full place with other waste containers for disposal by EH&S.
- All other waste can be collected in the waste container under the hood which contains 34 mL bleach per 500 mL waste. Waste should sit for at least 30 minutes following the last addition to be disposed of by pouring down the sink. Alternatively, a liquid waste container (50 mL tube) can be used on the bench then transferred into the waste container under the hood.
