# Supplementary material for: Histone modification analysis reveals common regulators of gene expression in liver and blood stage merozoites of Plasmodium parasites
Source: Epigenetics Chromatin. 2023 Jun 15;16:25. doi: 10.1186/s13072-023-00500-y (PMC10268464; doi:10.1186/s13072-023-00500-y)
Supplement: Supplementary file 3 — Additional file 3. Additional methods. [file 13072_2023_500_MOESM3_ESM.zip › Suppl_Methods/Parasite culture.docx]

**Parasite Culture**

Materials:

| **Item** | **Preparation** | **Storage** |
| --- | --- | --- |
| Complete medium | See Medium for P. falciparum Culture protocol | 37°C bead bath or 4°C |
| Washed RBCs | See Washing Packed Erythrocytes (RBCs) for P. falciparum Culture protocol | 4°C  4 weeks |
| Giemsa stain | 37 mL deionized water + 1.5 mL Giemsa stain | Stock in flammable chemicals cabinet |
| Microscopy slides |  |  |

Notes:

- Cultures are maintained at 5% hematocrit (1 x 10^8^ cells per 1% hematocrit)
- A single ring will develop into a single schizont. A single schizont will produce multiple rings (generally 2-3 rings per schizont, re-invasion rates vary based on culture conditions)
- *P. falciparum* has a 48-hour life cycle
- Medium is changed every day (except Saturday)
- 25-cm^2^ flask: 0.8 mL washed RBCs (50% hematocrit) + 7.2 mL complete medium, total 8 mL
- 75-cm^2^ flask: 2.5 mL washed RBCs (50% hematocrit) + 22.5 mL complete medium, total 25 mL

Protocol:

Feeding and Counting Parasite Cultures

1. Remove flask from incubator being careful not to disturb the settled layer of RBCs at the bottom of the flask
2. Using a 10 mL serological pipette, remove medium from (20-23 mL for 75- cm^2^ or 5-7 mL for 25- cm^2^) the flask being careful not to aspirate RBCs. *If culture is mixed up, spin for 5 min (250 x g, RT, acc=9, dec=1) to pellet the RBCs then remove medium and take ~1 uL of the pellet to make a smear.*
3. Tilt the flask so the remaining medium collects at the bottom. Scratch the RBC layer with a 1 mL or 2 mL pipette. Dot the RBCs on the pipette tip on a microscopy slide. Smear the RBC drop with a second slide to generate a monolayer of RBCs
4. Immediately fix the slide in methanol. *Fix for at least 30 seconds, extended fixation times (over 1 hour) can impact the quality of the slide.*
5. Add a volume of complete medium equivalent to what was removed from the flask
6. Return flask to the incubator to maintain in 5% CO_2_ and 5% O_2_ at 37°C
7. Remove the slide from the methanol and allow to dry
8. Prepare the Giemsa stain. Remove water from staining chamber and rinse once with deionized water. Add 37.5 mL of deionized water to chamber. Add 1.5 mL of Giemsa stain to the chamber using a Pasteur pipette
9. Place the slide in the Giemsa stain for at least 30 minutes
10. Remove slide from the Giemsa stain and rinse with water. Dry the slide using the air nozzle at the bench
11. Place used Giemsa stain in waste bottle and fill chamber with deionized water
12. Assess parasitemia of culture by looking at slide with the 100X objective of the microscope. *Count the number of infected RBCs from 5 areas within the “feathered edge of the smear” that contain roughly 100 RBCs (RBCs should be in clusters with white gaps visible between groups, see image). Count rings and mature parasites (any parasite that is not a ring) separately. Take the average number of rings and the average number of mature parasites from the 5 sections counted to calculate the ring and mature parasitemia. Record these numbers.*
13. Dilute culture appropriately

Waste:

- Medium and culture: Waste container under the hood which contains 34 mL bleach per 500 mL waste. Waste should sit for at least 30 minutes following the last addition to be disposed of by pouring down the sink.
- Slides: Sharps container.
- Giemsa stain: Giemsa Waste container (labeled *Giemsa Waste, 0.4% (w/v) in Methanol*). Once full, the container can be placed with the other chemical waste bottles for disposal by EH&S.

Diluting Cultures

| **Flask Size/Dilution** | **Culture (mL)** | **Washed RBCs (mL)** | **Complete Medium (mL)** |
| --- | --- | --- | --- |
| 75-cm^2^/1:2 | 12.6 | 1.25 | 11.2 |
| 75-cm^2^/1:3 | 8.4 | 1.7 | 14.4 |
| 75-cm^2^/1:4 | 6.3 | 1.9 | 16.8 |
| 75-cm^2^/1:6 | 4.2 | 2.1 | 18.7 |
| 25-cm^2^/1:2 | 4 | 0.4 | 3.6 |
| 25-cm^2^/1:3 | 2.7 | 0.54 | 4.8 |
| 25-cm^2^/1:4 | 2 | 0.6 | 5.4 |
| 25-cm^2^/1:6 | 1.3 | 0.67 | 6 |
