# Supplementary material for: Histone modification analysis reveals common regulators of gene expression in liver and blood stage merozoites of Plasmodium parasites
Source: Epigenetics Chromatin. 2023 Jun 15;16:25. doi: 10.1186/s13072-023-00500-y (PMC10268464; doi:10.1186/s13072-023-00500-y)
Supplement: Supplementary file 3 — Additional file 3. Additional methods. [file 13072_2023_500_MOESM3_ESM.zip › Suppl_Methods/Parasite synchronization.docx]

**Parasite Synchronization**

Materials:

| **Item** | **Preparation** | **Storage** |
| --- | --- | --- |
| 5% (wt/vol) D-sorbitol | 25 g sorbitol in 500 mL milliQ water, filter sterilize with 0.22 um bottle top filter | 4°C  3 months |
| Complete medium | See Medium for *P. falciparum* Culture protocol | 37°C bead bath or 4°C |

Notes:

- Sorbitol must be warmed to 37°C before use
- Only sync cultures with high percentage of young rings (<10 hours). Older parasites are killed during the procedure.

Protocol:

1. Pre-warm sorbitol
2. Transfer the culture to a 50 mL tube. *Cultures from 2 flasks can be combined in a single 50 mL tube if syncing multiple tube with the same parasitemia.*
3. Centrifuge for 5 minutes at 250 x g at RT (acc=9, dec=1)
4. Remove the supernatant by pipetting
5. Estimate the pellet volume and resuspend in 10x that volume of 5% sorbitol. *For a 75-cm^2^ flask: pellet volume is 1.25 mL, resuspend in 12.5 mL sorbitol. For a 25-cm^2^ flask: pellet volume is 0.4 mL, resuspend in 4 mL sorbitol.*
6. Vortex vigorously for 30 seconds to disrupt old RBCs and mature parasites
7. Incubate for 8 minutes at 37°C
8. Vortex for 15 seconds
9. Centrifuge for 5 minutes at 250 x g at RT (acc=9, dec=1)
10. Remove the supernatant by pipetting
11. Wash the RBCs with 5x the original pellet volume of complete medium
12. Centrifuge for 5 minutes at 250 x g at RT (acc=9, dec=1)
13. Remove the supernatant by pipetting
14. Resuspend the pellet in the original culture volume of complete medium and transfer to a clean flask
15. Return the culture to the incubator

Waste:

- All waste can be collected in the waste container under the hood which contains 34 mL bleach per 500 mL waste. Waste should sit for at least 30 minutes following the last addition to be disposed of by pouring down the sink.
