# Supplementary material for: Histone modification analysis reveals common regulators of gene expression in liver and blood stage merozoites of Plasmodium parasites
Source: Epigenetics Chromatin. 2023 Jun 15;16:25. doi: 10.1186/s13072-023-00500-y (PMC10268464; doi:10.1186/s13072-023-00500-y)
Supplement: Supplementary file 3 — Additional file 3. Additional methods. [file 13072_2023_500_MOESM3_ESM.zip › Suppl_Methods/Saponin parasite isolation.docx]

**Saponin Parasite Isolation**

Materials:

| **Item** | **Preparation** | **Storage** |
| --- | --- | --- |
| 1.5% (wt/vol) saponin, **COLD** | 0.75 g saponin + 50 mL PBS, sterile filtered with syringe and 0.22 um filter | 4°C  6 months |
| MilliQ water, **COLD** | Sterile filter with 0.22 um bottle top filter | 4°C  1 year |
| 1X PBS, **COLD** | Use pre-bottled PBS | 4°C |
| Ice |  | Ice machine |

Notes:

- Saponin, water, and PBS must be ice cold

Protocol:

1. Pre-cool centrifuge to 4°C
2. Prepare 0.15% saponin and place on ice until use. *Dilute 1.5% stock solution in 10X cold, sterile milliQ water. Per flask isolated (assuming a 1.25 mL pellet of RBCs) dilute 1.4 mL 1.5% saponin in 8.6 mL cold water.*
3. Aspirate media from flask and make a smear to determine parasitemia at the time of isolation
4. Add 5 mL of cold 1X PBS to flask and resuspend settled RBCs
5. Transfer culture to a 50 mL tube. *When isolating parasites from multiple flasks, up to 4 flasks can be combine in a single 50 mL tube.*
6. Rinse the flask with 3 mL PBS and add this to the 50 mL tube
7. Fill the 50 mL to 45 mL with cold PBS
8. Centrifuge at 250 x g for 5 minutes at 4°C (acc=1, dec=1)
9. Remove the supernatant by pipetting and note the volume of the pellet
10. Add 5x the pellet volume of 0.15% saponin to the RBC pellet and mix by pipetting. *For a single, 75-cm^2^ culture flask estimate adding 6.25 mL saponin. Mixture should become clearer and dark red as RBCs are lysed.*
11. Incubate on ice for 10 minutes, mixing the tube by inverting every 3-4 minutes.
12. Centrifuge at 4,000 x g for 10 minutes at 4°C (acc=9, dec=1)
13. Pipette off the supernatant
14. If a noticeable number of RBCs remain in the pellet (pellet will be red in color), repeat the saponin treatment for 5 minutes.
15. Resuspend the pellet in 45 mL cold PBS
16. Centrifuge at 4,000 x g for 10 minutes at 4°C (acc=9, dec=1)
17. Pipette off the supernatant
18. Wash with 45 mL PBS until supernatant is clear after centrifugation
19. Remove supernatant by pipetting
20. Resuspend pellet in 1 mL cold PBS and transfer to a 1.5 mL tube
21. Centrifuge at 4,000 x g for 5 minutes at 4°C (using tabletop centrifuge)
22. Remove the supernatant by pipetting and continue to downstream protocol or application

Waste:

- All waste can be collected in the waste container under the hood which contains 34 mL bleach per 500 mL waste. Waste should sit for at least 30 minutes following the last addition to be disposed of by pouring down the sink.
