# Supplementary material for: Histone modification analysis reveals common regulators of gene expression in liver and blood stage merozoites of Plasmodium parasites
Source: Epigenetics Chromatin. 2023 Jun 15;16:25. doi: 10.1186/s13072-023-00500-y (PMC10268464; doi:10.1186/s13072-023-00500-y)
Supplement: Supplementary file 3 — Additional file 3. Additional methods. [file 13072_2023_500_MOESM3_ESM.zip › Suppl_Methods/Washing packed erythrocytes for parasite culture.docx]

**Washing Packed Erythrocytes (RBCs) for *P. falciparum* Culture**

Materials:

| **Item** | **Preparation** | **Storage** |
| --- | --- | --- |
| Incomplete medium, **COLD** | See Medium for *P. falciparum* Culture protocol | 4°C |
| Complete medium | See Medium for *P. falciparum* Culture protocol | 37°C bead bath or 4°C |
| 1 L sterile glass bottle |  |  |

Notes:

- Type AB positive ACD Packed Cells from Interstate Blood Bank Inc. (Memphis, TN). Can be store at 4°C for a few hours until washing.
- Amount of blood to wash depends on the amount of parasites being cultured. Generally, 6-8 tubes of blood will last 4 weeks.
- Keep one tube of old washed blood until you are sure the parasite grow well in the new blood.

Protocol:

1. Pre-cool centrifuge to 8°C
2. Clean scissors and draining tube of bag with packed erythrocytes using 70% ethanol
3. Transfer the packed erythrocytes into sterile glass bottle by cutting the draining tube and letting the blood drip into the bottle
4. Pipette 12.5 mL of packed erythrocytes into a 50 mL tube
5. Pipette 25 mL of packed erythrocytes into a 50 mL and store at 4°C as unwashed RBCs. *This can be washed later in case we run out of washed blood.*
6. Add 10 mL cold incomplete medium to the 50 mL tube with blood
7. Close lid tightly and invert 10x
8. Centrifuge at 500 x g for 8 minutes at 8°C (acc=9, dec=1)
9. Pipette off the supernatant with buffy coat
10. Add 10 mL cold incomplete medium
11. Close the lid tightly and invert 10x
12. Centrifuge at 500 x g for 8 minutes at 8°C (acc=9, dec=1)
13. Pipette off supernatant with buffy coat
14. Repeat wash with incomplete medium is there is still a noticeable buffy coat above the packed erythrocytes. If there is no visible buffy coat, continue to the next step.
15. Add an equal volume of complete parasite medium to the packed erythrocytes to make it 50% hematocrit
16. Store at 4°C

Waste:

- Packed Erythrocytes and old washed RBCs: Pour old washed RBCs into bottle containing left over packed erythrocytes. Add bleach to final concentration of about 0.525% hypochlorite (about a 15X dilution of bleach – check dilution table on flow cabinet for exact values). Let sit for 30 minutes under the flow cabinet (blood congeals if left for much longer and can be hard to clean). Pour down sink.
- Washes: Waste container under the hood which contains 34 mL bleach per 500 mL waste. Waste should sit for at least 30 minutes following the last addition to be disposed of by pouring down the sink.
